# Identification and functional characterization of new missense SNPs in the coding region of the *TP53* gene

**DOI:** 10.1038/s41418-020-00672-0

**Published:** 2020-11-30

**Authors:** Flora Doffe, Vincent Carbonnier, Manon Tissier, Bernard Leroy, Isabelle Martins, Johanna S. M. Mattsson, Patrick Micke, Sarka Pavlova, Sarka Pospisilova, Jana Smardova, Andreas C. Joerger, Klas G. Wiman, Guido Kroemer, Thierry Soussi

**Affiliations:** 1 Equipe Labellisée par la Ligue Contre le Cancer, Université Paris Descartes, Université Sorbonne Paris Cité, Université Paris Diderot, Sorbonne Université, INSERM U1138, Centre de Recherche des Cordeliers, Paris, France; 2 Department of Oncology–Pathology, Bioclinicum, Karolinska Institutet, Stockholm, Sweden; 3 Department of Life Science, Sorbonne Université, Paris, France; 4 Metabolomics and Cell Biology Platforms, Institut Gustave Roussy, Villejuif, France; 5 Department of Immunology, Genetics and Pathology, Uppsala University, Uppsala, Sweden; 6 Department of Internal Medicine—Hematology and Oncology, University Hospital Brno and Faculty of Medicine, Masaryk University, Brno, Czech Republic; 7 Central European Institute of Technology, Masaryk University, Brno, Czech Republic; 8 Faculty of Science, Department of Experimental Biology, Masaryk University, Brno, Czech Republic; 9 Institute of Pharmaceutical Chemistry, Johann Wolfgang Goethe University, Max-von-Laue-Str. 9, 60438 Frankfurt am Main, Germany; 10 Buchmann Institute for Molecular Life Sciences and Structural Genomics Consortium (SGC), Max-von-Laue-Str. 15, 60438 Frankfurt am Main, Germany; 11 Pôle de Biologie, Hôpital Européen Georges Pompidou, AP-HP, Paris, France; 12 Department of Women’s and Children’s Health, Karolinska University Hospital, Stockholm, Sweden; 13 Present Address: Department of Immunology, Genetics and Pathology, Uppsala University, Uppsala, Sweden; 14 Present Address: Cell Death and Drug Resistance in Lymphoproliferative Disorders Team, INSERM U1138, Centre de Recherche des Cordeliers, Paris, France

**Keywords:** Tumour-suppressor proteins, Genetics research

## Abstract

Infrequent and rare genetic variants in the human population vastly outnumber common ones. Although they may contribute significantly to the genetic basis of a disease, these seldom-encountered variants may also be miss-identified as pathogenic if no correct references are available. Somatic and germline *TP53* variants are associated with multiple neoplastic diseases, and thus have come to serve as a paradigm for genetic analyses in this setting. We searched 14 independent, globally distributed datasets and recovered *TP53* SNPs from 202,767 cancer-free individuals. In our analyses, 19 new missense *TP53* SNPs, including five novel variants specific to the Asian population, were recurrently identified in multiple datasets. Using a combination of in silico, functional, structural, and genetic approaches, we showed that none of these variants displayed loss of function compared to the normal *TP53* gene. In addition, classification using ACMG criteria suggested that they are all benign. Considered together, our data reveal that the *TP53* coding region shows far more polymorphism than previously thought and present high ethnic diversity. They furthermore underline the importance of correctly assessing novel variants in all variant-calling pipelines associated with genetic diagnoses for cancer.

## Introduction

The evaluation of germline and/or somatic *TP53* status is becoming mandatory in a number of clinical situations. In patients with Li–Fraumeni syndrome or families with hereditary breast and ovarian cancer syndrome, the early identification of germline *TP53* mutations has been shown to be highly beneficial for disease surveillance [1]. In chronic lymphocytic leukemia, acute myeloid leukemia, and myelodysplastic syndrome, somatic *TP53* variants are associated with poor prognosis, and furthermore, they are now investigated in routine clinical practice with the aim of identifying patients who would benefit from specific treatment [2, 3].

It is therefore essential that the identification and classification of *TP53* variants meet quality requirements for clinical diagnostics used in personalized medicine. However, the analysis of somatic or germline *TP53* variants faces a range of challenges. In tumors, the somatic origin of mutations can be inferred via comparison with matched normal DNA from the same patient. Unfortunately, this material is often missing, necessitating indirect assumptions made using databases that include the most frequent germline variants observed in the human population. For the analysis of germline variants, data are directly compared to population databases. In both cases, the accuracy of the results will depend on the characteristics of the population databases. Among those characteristics are not only the increasing number of rare variants found in the human population but also the ethnic diversity of this latter.

The advent of massively parallel sequencing (next-generation sequencing, or NGS) has shown that the human genome includes far more genetic variation than anticipated, with many variants detected at frequencies far below the formerly used 1% limit [4]. To illustrate, the most frequent build (151) of dbSNP includes 335 million variants. However, several studies have questioned the quality of the entries, among which both pathogenic and nonpathogenic *TP53* variants are included [5, 6]. In contrast, data from the 1000 Genomes Project have been highly curated, but the small number of individuals (2504) included in the project does not enable the full coverage of low-frequency variants. To circumvent this issue, the Exome Aggregation Consortium released the ExAC database, which compiles whole-exome sequencing data from 60,706 individuals [7]. ExAC includes not only data from the 1000 Genomes Project but also constitutional data from unrelated individuals sequenced as part of various disease-specific and population genetics studies. In 2016, ExAC became the Genome Aggregation Database (gnomAD) with an enlarged dataset providing information from 123,136 exome sequences and 15,496 whole-genome sequences from unrelated individuals (http://gnomad.broadinstitute.org/). Both ExAC and gnomAD have been widely used as a substitute for or a complement to dbSNP and are currently used in multiple analytical pipelines.

Two missense SNPs in the *TP53* gene have been identified and extensively characterized. SNP rs1042522 (p.P72R) is common in all world populations but the ancestral allele (Pro) shows a north-south gradient ranging between 0.2 and 0.7 [8]. The second SNP, rs1800371 (p.P47S), has been shown to be specific to the African population, albeit at a lower frequency (0.01–0.02) [9]. Both SNPs are included in ClinVar and considered benign according to American College of Medical Genetics and Genomics (ACMG) criteria.

In the present study, using a combination of in silico, in vitro, structural and genetic approaches, we addressed a two-tiered question: (i) is it possible to identify novel and infrequent *TP53* SNPs in the human population and (ii) what is the potential pathogenicity of these variants? Using aggregated and population-specific databases, in silico and functional studies, and clinical data, we identify 19 new exonic *TP53* SNPs including five variants specific to the Asian population.

## Results

### Identification of new *TP53* SNPs in the human population

**Fig. 1:**
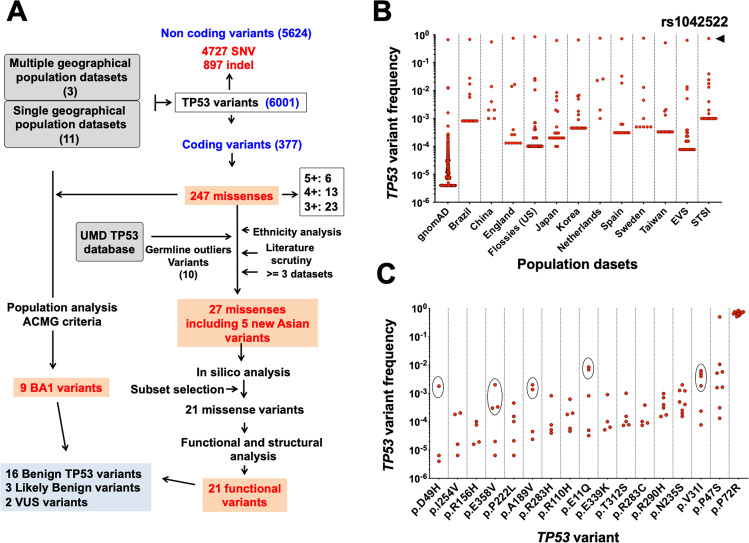
Identification of new recurrent *TP53* variants in the human population. **A** Flow chart of the strategy used to identify new germline *TP53* variants in the human population. Three general population datasets and 11 country-specific datasets, the latter not included in the former, were screened for *TP53* variants. The 388 missense *TP53* variants were analyzed for potential constitutional variants as described in the text. **B** Allele frequency distribution of exonic *TP53* variants in the 14 populations. rs1042522 (p.P72R) was found at high frequency, regardless of the dataset as shown at the top of the figure. **C** Allele frequency of *TP53* variants *TP53* variants found in the present study. For each variant found in four or more population datasets, the allele frequency for each dataset is shown. For five variants, the Asian populations have been circled to show the preferential origin of these variants. (A full vesrion of tis figure is available in Fig S1d).

To identify novel infrequent *TP53* SNPs in the human population, fourteen independent datasets compiled from individuals without neoplastic disease (202,767 individuals) were screened for variants located anywhere in the entire *TP53* gene (Fig. 1A and Table S1). Three datasets included aggregated data from multiple studies, whereas 11 comprised data from specific countries. These latter were globally distributed to increase the identification of population-specific variants. Furthermore, data overlap between these datasets was minimal, which avoided excessive redundancy in the analysis (“Materials and methods”).

We thus uncovered 6001 different *TP53* variants at frequencies ranging from 0.8 to 10^−6^, and, as expected, most of them were located in noncoding sequences of the *TP53* gene (5628 noncoding and 373 coding) (Table S2a and Fig. S1). For the present work, we focused our analysis on missense variants. Other variants such as intronic, synonymous, or specific *TP53* isoform-targeting variants are fully described in the Supplementary Information*.* The particular case of rs138729528, (p.R175C), a passenger mutation found at high frequency in human tumors and in the normal population is also discussed in the Supplementary Information. We identified 247 missense variants in the 14 datasets, with 4, 6, and 14 variants found in more than 5, 4, and 3 datasets, respectively (Figs. 1A,  S1b and c, and Table S2c). Their frequencies ranged from 0.8 to 4 × 10^−6^. The two most common benign *TP53* SNPs found in the human population, i.e., rs1042522 (p.P72R) and rs1800371 (p.P47S), are included in this list (found in 14 and 7 datasets, respectively) (Figs. 1B, C and S1b and c). The statuses of the remaining variants were unknown, but their recurrences in various independent datasets suggested that they could also be rare constitutional *TP53* SNPs.

**Fig. 2:**
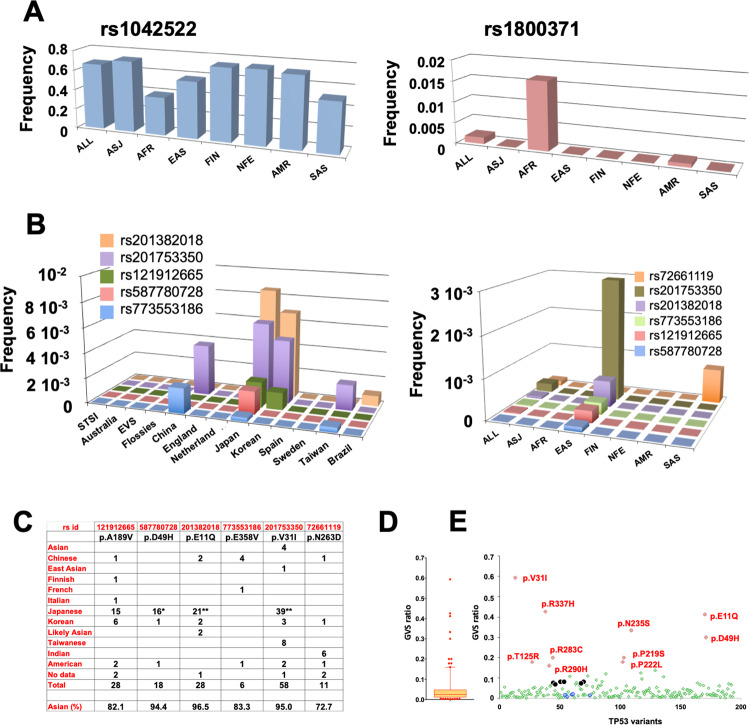
Identification of specific *TP53* variants in the Asian population. **A** Geographical distribution of rs1042522 (p.P72R) and rs1800371 (p.P47S) in the various subsets of gnomAD. **B** Distribution of six new potential constitutional *TP53* variants in the various subsets of gnomAD (left) and in the specific population datasets (right). ALL all populations, AFR African/African American, AMR admixed American, ASJ Ashkenazi Jewish, EAS East Asian, FIN, Finnish, NFE non-Finnish European, OTH other unassigned populations, SAS South Asian. **C** Ethnicity origin of the six variants described in the literature of the UMD_*TP53* database. * and **: including two or three different cell lines, respectively. **D, E** Germline-to-somatic (GVS) ratio of *TP53* variants in the UMD_*TP53* database. **D** A box-plot analysis of the GVS ratio shows that the vast majority of *TP53* variants had similar values. Ten outlier variants above the 95% confidence interval had high frequency as germline variants in the database and could be benign SNPs. *TP53* variants found below the 5% interval were never found as germline variants in the database; they corresponded to specific *TP53* variants associated with carcinogen exposure, such as p.R249S found in aflatoxin B1-associated hepatocellular carcinoma, and p.V157F found in tobacco-associated lung cancer. The box-and-whisker plot shows the interquartile range (box), median value (horizontal line inside the box), and full-range distribution (whiskers) for the GVS ratio. **E** Detailed analysis of the germline ratio. The ten outlier variants are shown in red. Values for six hot spot *TP53* variants are shown in black. This analysis was performed on 195 *TP53* variants with at least five occurrences as germline variants in the database.

The gnomAD dataset includes population data that enables subdivision into eight classes according to ancestry. rs1042522 (p.P72R) was found in all ethnic groups at a frequency ranging from 0.4 to 0.7 (Fig. 2A). Our analysis confirmed previous observations of a lower frequency for rs1042522 in the African population (0.4 in our analysis). Those observations have led some authors to hypothesize that the frequency of this polymorphism may be latitude-dependent [8]. In the present analysis, we also confirmed the quasi-exclusivity of rs1800371 (p.P47S) in the African population, as previously observed in other studies [10] (Figs. 2A and S2). Unexpectedly, our analysis uncovered five novel variants that were present in only the East Asian (EAS) population of gnomAD (rs201753350; (p.V31I), rs587780728; (p.D49H), rs201382018; (p.E11Q), rs121912665; (p.A189V), and rs773553186; (p.E358V)) (Fig. 2B). This specificity was confirmed by the analysis of datasets specific to Asian populations (Taiwan, Korea, China, and Japan) (Figs. 2B and S2). These five variants are also included in such cancer mutation databases as Cosmic, UMD, and IARC. Because normal DNA is usually not available, the constitutional origin of these variants may have been missed. The UMD_*TP53* database included 146 entries for these five variants published in 64 reports. For all of these latter, the ethnicity of all patients was carefully checked and shown to be highly predominantly Asian (139 out of 146 patients) (Fig. 2C and Table S3). Notably, in two American multicenter studies that included patients from various geographical regions, these variants were only identified in Asian patients. We also noticed that the nine cell lines expressing any one of these variants originated from Japanese patients (Table S3). Taken together, these results clearly showed that the five novel constitutional variants were indeed specific to the Asian population. Whether any of these infrequent variants originated from a single event, i.e., a founder mutation, is currently unknown. A sixth rare variant, rs72661119; (p.N263D), was identified specifically in the South Asian (SAS) population of gnomAD with multiple reports originating from India (Fig. 2C and Table S3).

The various *TP53* mutations databases include both somatic variants from multiple types of cancer and germline variants from a range of families or patient cohorts with Li–Fraumeni syndrome or hereditary breast and ovarian cancer syndrome. As discussed above, normal tissue was not available for many of the 80,000 tumors included in the UMD_*TP53* database; thus assessments of variant origin had often been done by comparison with dbSNP, which includes a limited number of *TP53* population variants. It is therefore likely that infrequent germline variants were mistakenly identified as somatic variants, leading to database contamination. We therefore evaluated the germline-to-somatic ratio for each *TP53* variant in the UMD_*TP53* database. As shown in Fig. 2D, the distribution of this ratio for the majority of *TP53* variants, including all *TP53* mutation hot spots, was quite low, ranging from 0.03 to 0.056. Ten outlier variants displayed a high ratio, suggesting that they may be undetected germline variants (Fig. 2E). Indeed, nine of these outlier variants are included in the 247 missense variants described above, with seven of them identified in three or more datasets. They also included three of the variants detected in the Asian population.

In a study published by our group, p.N235S was detected in a patient with lung cancer and defined as somatic [11]. However, when we reanalyzed this patient by sequencing normal DNA, we found that the variant was germline (Fig. S3a). Similarly, reanalysis of normal DNA from chronic lymphocytic leukemia patients confirmed the germline origin of this variant as well as variants p.R290H, p.R283C, and p.G360A (Fig. S3b–f).

Our analysis suggests that the coding region of the *TP53* gene includes far more ethnically distributed SNPs than was previously thought, and that some of them are mistakenly included as somatic variants in a number of databases. It is therefore essential to determine whether or not the p53 protein encoded by these SNPs is functional and how they must be classified according to ACMG criteria.

### Predictive and functional analysis of *TP53* variants; in silico analysis

**Fig. 3:**
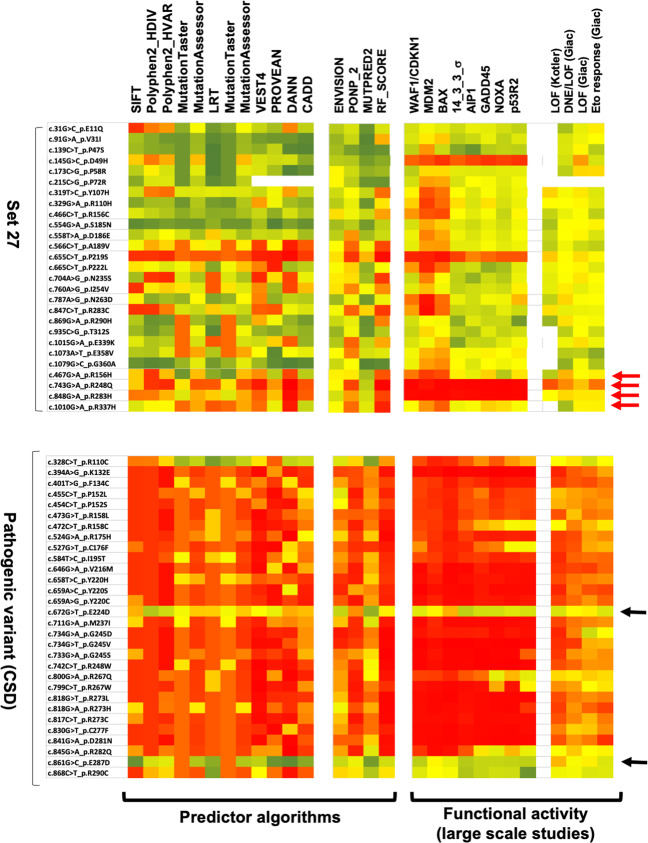
Predictive and functional profile of *TP53* variants. The heat map corresponds to either the loss of function (right panels) or the predictive impact (left panels) of each *TP53* variant ranging from 0 (red) to 1 (green), with the lowest score being the most deleterious. *TP53* variants from set 27 and from pathogenic *TP53* from the CSD are shown, respectively, in the upper and lower part of the figure. Functional activities defined experimentally by the three large-scale analyses performed by Kato et al. (transcriptional activity in yeast using eight different *TP53* response elements), Giacomelli et al. (dominant negative activity, growth arrest i.e., loss of function (LOF) or response to etoposide), and Kotler et al. (growth arrest in mammalian cells i.e., LOF) are shown in the right panels of the figure. Computational algorithms used to predict the deleteriousness of *TP53* activity are shown in the left panels of the figure. Scores from the 12 algorithms in the left panels were derived from the dbSNFP, whereas the four scores were obtained from each predictor. A full description of the scorings used in the figure can be found in Table S4. Red arrows indicate pathogenic CSD *TP53* variants included in gnomAD; black arrows indicate CSD *TP53* variants known to impair *TP53* splicing.

Computational algorithms to predict the functional effect of missense variants were performed on 27 SNPs (set 27) found in at least three datasets, and on the outlying germline variants identified in the analysis of the UMD*_TP53* database described in the previous section (Table S2c). First, we used the prediction scores from dbNSFP, a database that compiles scores from several popular algorithms along with a conservation score and other related information for every potential single nucleotide variant in the human genome [12] (“Material and methods”). Most of these algorithms are based on sequence homology and/or the physical properties of amino acids (Table S4). They are widely used for a global estimation of the deleteriousness of missense variants but they are not specific to any protein. We also used new in silico mutation prediction tools such as Revel or Envision that have been trained on known pathogenic and neutral nsSNVs. Among the 27 analyzed *TP53* variants, 4 were shown to be included in the cancer shared dataset (CSD), including the hot spot variant p.R248Q (Fig. 3). Although the majority of individuals included in the various datasets were selected for nonneoplastic disease, it is known that the frequency of de novo germline *TP53* mutation in the human population is high, ranging from 1/5000 to 1/20,000 [13, 14]. It is therefore not surprising that among the 138,632 individuals in gnomAD as well as those in other population datasets, some may have been carrying such de novo mutations, although without cancer at the time of inclusion [15]. The rank scores of *TP53* variants found in set 27 were always associated with a low pathogenic score, in contrast to the scores of the CSD-associated variants (Fig. 3). We note too that except for a few variants, there was considerable heterogeneity among the employed predictive tools.

A unique benefit *TP53* has over other genes is the availability of quantitative functional data for all missense variants. In a pioneer study, Kato et al. engineered 2100 *TP53* variants and assessed their transactivation activity in a yeast assay using eight different *TP53* response elements. Their approach yielded a quantitative score for each variant [16]. Our analysis of the residual activity of the variants showed clearly that most *TP53* variants, with the exception of those included in the CSD, were still functional (Fig. 3).

Two recent studies looked at large libraries to explore the loss of function of *TP53* variants in mammalian cells and extend the functional analysis to a range of properties including growth arrest, apoptosis, dominant negative effect, or response to etoposide stress [17, 18]. In both studies, each *TP53* variant was given a score indicating its potency to alter *TP53* function. These novel scores agreed well with the transcriptional data collected in the yeast studies and showed clearly that there was good correlation between yeast and mammalian data (Fig. 3). Ranking data from the work of Kotler et al. showed that the 11 variants in the central DNA-binding domain (DBD) did not cause loss of activity (Fig. S4) [17].

This analysis using functional data from large-scale, saturating mutagenesis showed that most of these variants were likely to be functional, in contrast to their CSD-associated counterparts. Furthermore, as expected, using *TP53*-functional criteria improved the classification of the different variants compared to predictive algorithms.

### Functional analysis

**Fig. 4:**
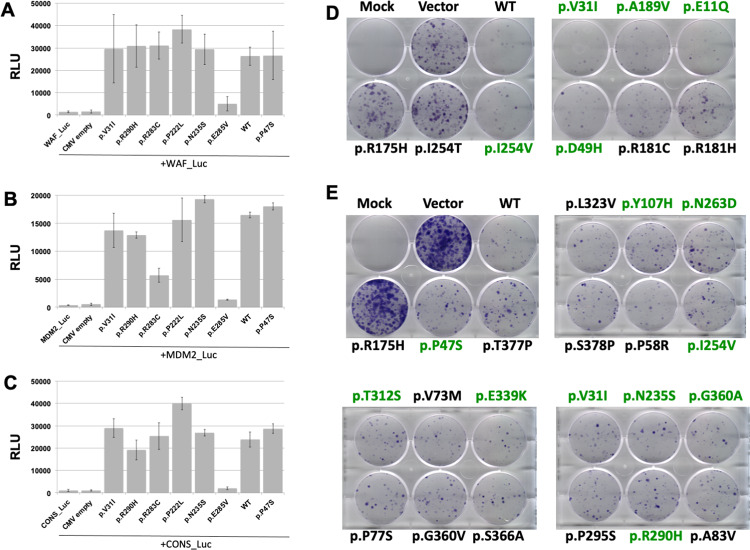
*TP53* variants found in the human population do not display loss of activity. Transcriptional activity analysis: *WAF1* (**A**), *MDM2* (**B**), and CONS (**C**) promoters upstream of the luciferase reporter were transiently transfected in H1299 cells with a range of *TP53* variants. Luciferase activity in the cell lysates was determined at 24 h after transfection (see also Fig. S6 for other *TP53* variants). Colony growth arrest: H1299 cells were transfected with various *TP53* variants and selected for 2 weeks in a selective medium with G418 before staining with crystal violet. Mock: non-transfected cells. *TP53* variants with impaired activities (p.R175H and p.I254T) were used as controls. Two independent experiments are shown in this figure (**D, E**). Variants from set 21 are shown in green. Other experiments and quantitative analyses of colony numbers are available in Fig. S8.

Although our population and prediction analyses suggested strongly that these newly discovered variants were functional, we nonetheless chose a subset of 21 *TP53* variants (set 21) for a detailed functional analysis (Tables 1 and S1). Transcriptional activities were tested using three different assays. FASAY, a yeast assay, initially described by Iggo et al. has been widely used in the clinic for rapid screening of *TP53* status and was furthermore used by Kato et al. to define *TP53* activity as described in the previous section [19]. FASAY enables the detection of variants with particular temperature sensitivities (Fig. S5). In our work, all tested variants were shown to be fully active at three temperatures (25, 30, and 35 °C) using the WAF1 promoter [20]. Cancer hot spot variants p.I254T (temperature-sensitive) and p.R175H (totally defective) were used as controls (Tables 1 and S5). *TP53* variants were also expressed in the p53-null cell line H1299 to determine their function based on transactivation of a *TP53*-dependent luciferase reporter with various *TP53* response elements. These variants had profiles indistinguishable from that of wild-type *TP53* (Figs. 4A–C, S6 and Tables 1, S5).

**Table 1:** Functional analysis and classification of 21 *TP53* variants found in the human population^a^.

*TP53*_variant	Functional analysis^b^	Growth suppression (Kotler et al.)	DNE/LOF (Giacomelli et al.)	LOF (Giacomelli et al.)	Population classification (ACMG)	Population classification (ACMG)^c^	ClinVar data^d^
c.31G>C_E11Q	Functional	No data	Functional	Functional	BA1 (Japan; Korea); BS1(EAS)	Benign (BA1; BS3; BP4)	LB (2) VUS (4)
c.91G>A_V31I	Functional	No data	Functional	Functional	BA1 (China; Japan; Korea; Taiwan; gnomAD_EAS)	Benign (BA1; BS3; BP4)	B (1) LB (4) LP (1) VUS (3) Star (1)
c.139C>T_P47S	Functional	No data	Functional	Functional	BA1 (Brazil; Flossies; GnomAD; Kaviar; EVS; gnomAD_AFR)	Benign (BA1; BS3; BP4)	B (10) VUS (1) Star (1)
c.145G>C_D49H	Functional	No data	Functional	Functional	BA1 (Japan)	Benign (BA1; BS3; BP4)	VUS
c.173C>G_P58R	Functional	No data	Functional	Functional	BS1 (gnomAD_AFR; Flossies; EVS)	Benign (BA1; BS3; BP4)	LB (6) VUS (1)
c.215C>G_P72R	Functional	No data	Functional	Functional	BA1 (all datasets)	Benign (BA1; BS3; BP4)	B Star (3)
c.319T>C_Y107H	Functional	Functional	Functional	Functional	BA1 (gnomAD_AFR); BS1 (Flossies)	Benign (BA1; BS3; BP4)	LB (3) VUS (4)
c.329G>A_R110H	Functional	Functional	Functional	Functional	–	Likely benign (BS3; BP4)	VUS
c.466C>T_R156C	Functional	Functional	Functional	Functional	–	Likely benign (BS3; BP4)	VUS
c.554G>A_S185N	Functional	Functional	Functional	Functional	–	Likely benign (BS3; BP4)	VUS
c.566C>T_A189V	Functional	Functional	Functional	Functional	BA1 (China)	Benign (BA1; BS3; BP4)	VUS
c.665C>T_P222L	Functional	Functional	Functional	Functional	–	VUS (BS3)	VUS
c.704A>G_N235S	Functional	Functional	Functional	Functional	BA1 (Australia); BS1 (FIN; Finland; NFE)	Benign (BA1;BS1;BP4)	B (1)LB (5) VUS (3) Star (1)
c.760A>G_I254V	Functional	Functional	Functional	Functional	–	VUS (PP3; BS3)	VUS
c.787A>G_N263D	Functional	Functional	Functional	Functional	SAS	Benign (BS1; BS3)	LB (1) VUS (4)
c.847C>T_R283C	Functional	Functional	Functional	Functional	EVS	Benign (BS1; BS3)	LB (1) LP (1) VUS (12)
c.869G>A_R290H	Functional	Functional	Functional	Functional	BS1 (Finland; Flossies; FIN)	Benign (BS1; BS3)	B (1) LB (1) VUS (10) Star (1)
c.935C>G_T312S	Functional	No data	Functional	Functional	BS1 (gnomAD_AFR)	Benign (BS1; BS3)	B (1)LB (1) VUS (4) Star (1)
c.1015G>A_E339K	Functional	No data	Functional	Functional	BS1 (gnomAD_EAS)	Benign (BS1; BS3)	LB (2) VUS (3)
c.1073A>T_E358V	Functional	No data	Functional	Functional	BA1 (China)	Benign (BA1; BS3; BP4)	LB (1) VUS (2) Star (2)
c.1079G>C_G360A	Functional	No data	Functional	Functional	BS1 (gnomAD_LAT; gnomAD_AFR)	Benign (BS1; BS3)	B LB
c.524G>A_R175H	Defective	Defective	Defective	Defective	–	Pathogenic (PS3; PS4; PM1; PP3)	P
c.761T>C_I254T	Defective	Defective	Defective	Defective	–	Pathogenic (PS3; PS4; PM1; PP3)	No data
c.1025G>C_R342P	Defective	Functional	Functional	Defective	–	Pathogenic (PS3; PS4; PM1; PP3)	LP P
c.428T>C_V143A	Defective	Defective	Defective	Defective	–	Pathogenic (PS3; PS4; PM1; PP3)	No data

*DNE* dominant negative effect, *LOF* loss of function.

^a^A complete version of this table with detailed description of all data is available in Table S5.

^b^Functional analysis includes transcriptional activity, apoptosis, and cell proliferation assays performed both in human cells or in yeast in the present study.

^c^For each classification, identity of the dataset is indicated in brackets. gnomAD data were split into eight geographical datasets (EAS, AFR, SAS, FIN, LAT, EAS, ASJ, and OTH) leading to a total of 22 datasets.

^d^Assessed June 2020.

To confirm the validity of this analysis on *TP53* regulation, the expression levels of endogenous *TP53* target genes were also analyzed by qPCR after transfection of several variants into H1299 cells (Fig. S7a, b and Table S5). Genes involved in several *TP53* pathways such as cellular response to DNA damage (*WAF1, ZMAT3, MDM2*), DNA repair (*DDB2*), metabolism (*TIGAR*), apoptosis (*FAS, APAF1*), or reactive oxygen species regulation (*SESN1*) were fully activated compared with pathogenic *TP53 variants*.

Two other functional assays, colony-formation assay and apoptosis, were also performed to assess the biological function of these variants (Figs. 4D, E and S8, S9a–c, and Table S5). Except for CSD *TP53* variants used as a negative control, no loss of activity was observed for the various SNPs. The collective results of our analysis showed that these latter were indistinguishable from wild-type *TP53*.

### Location of *TP53* variants and predicted impact

**Fig. 5:**
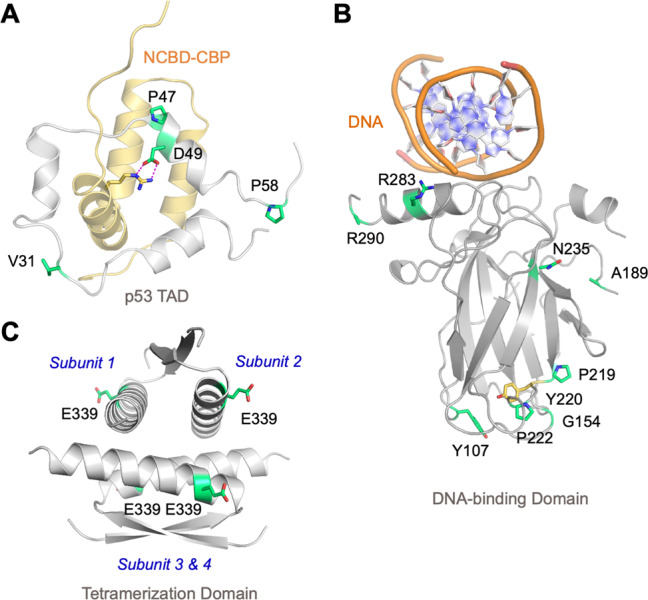
Location of *TP53* SNPs in the protein structure. **A** Solution structure of p53 TAD in complex with the nuclear receptor coactivator binding domain of CREB binding protein (NCBD-CBP) (PDB entry 2L14). **B** Crystal structure of the DBD bound to target DNA (PDB entry 3KZ8, chain A). **C** Crystal structure of the tetramerization domain (PDB entry 1C26). p53 domain structures in **A**–**C** are shown as cartoons in gray, with the SNP sites highlighted as green stick models. NCBD-CBP is shown as a light-brown cartoon, and the side chain of the arginine involved in an intermolecular salt bridge with p53 is shown as a stick model.

We mapped the identified *TP53* variants within the structure of p53. Quite a few SNPs were located in the intrinsically disordered N-terminal transactivation domain (TAD) and proline-rich region. They did not modify the molecular recognition feature for binding of the negative regulators MDM2/X or the TAZ1 domain of transcriptional coactivator p300. Interestingly, Asp49 within the nascent helix of the p53 TAD forms an intermolecular salt bridge with an arginine in complex with the nuclear receptor coactivator binding domain of the CREB binding protein (Fig. 5A), which has been suggested to contribute to binding specificity [21]. This specific salt-bridge partner was lost in p.D49H variants. The p.P72R variant reduced slightly the rigidity of the proline-rich region, which may serve as a stiff linker for projecting the TADs away from the core of the p53-DNA complex for optimal interaction with coactivators [22]. Whether this variant has an effect on cancer predisposition remains controversial/inconclusive [23].

All eight DBD variants affected residues on or close to the protein surface (Figs. 5B and S10a–f). Four of them, p.Y107H, p.G154S, p.P219L, and p.P222L, clustered on a surface patch, distant from the DNA-binding surface (and other functional interfaces), around the site of the cancer hot spot mutation p.Y220C. This latter creates an extended surface crevice and is highly destabilizing; it lowers the melting temperature of the DBD by about 8 °C, causing it to rapidly unfold and aggregate at body temperature [24]. Structural models of the variant DBDs indicated minor local structural rearrangements at the mutation sites. Stability estimates using the HoTMuSiC server predicted only relatively small changes of <2 °C in the melting temperature of the DBD for all DBD SNP variants, except for p.G154S and p.P219S, which were predicted to be destabilized by 3–4 °C compared with the wild type (Table S6). This remains significantly below the stability loss observed for the most deleterious structural cancer mutations, such as p.Y220C or the classical temperature-sensitive *TP53* variant p.V143A. Interestingly, Tyr107 is conserved in mammalian p53, but a histidine is found in many fish species and in the more thermodynamically stable sister proteins p63/p73. In the structure of the p.Y107H variant, the imidazole side chain of His107 potentially forms a hydrogen bond with the backbone oxygen of Ser149 (Fig. S10a).

The stability predictions were consistent with the observation of full activity at body temperature for all the variants. Although small changes in stability may have no functional impact, they may predispose carriers of the respective SNP to functional inactivation by other mildly-destabilizing mutations that would otherwise be harmless. Comparisons of the predictions with experimentally determined melting temperatures for a panel of cancer hot spot variants showed that they generally agreed well qualitatively, but that there were also notable exceptions. For example, in the case of the thermolabile p.R282W and zinc-binding deficient p.R175H cancer variants, the program failed to predict the drastic stability loss observed experimentally (Table S6). It will thus be of interest to determine the stability of the DBD SNP variants experimentally, to fully assess their impact on cancer risk.

The side chain of Ala189 in the extended L2 loop faces inwards, and the model of the p.A189V variant displayed altered hydrophobic packing against Arg196 and Tyr205 (Fig. S10c). The side chain of Asn235 in beta-strand S8 formed hydrogen bonds with two backbone oxygens (Lys139 and Tyr234) and the carboxylate group of Glu198 (Fig. S10d). This polar interaction network was lost in the p.N235S variant but may have been compensated for, at least in part, by alternative, potentially water-mediated hydrogen bonds formed by the serine hydroxyl. In the mouse structure, there is a lysine at this position, and a serine is found in various fish species.

The other two DBD variants, p.R283C and p.R290H, were located on the C-terminal helix that binds to the major groove of the p53 response elements (Fig. 5B). Arg283 sits close to the protein-DNA interface, but in most p53-DNA complexes, it does not interact directly with the phosphate backbone [25, 26], so no significant effect on DNA-binding affinity and specificity is expected. The closest distance between the guanidinium group and the phosphate backbone of DNA in Protein Data Bank (PDB) entry 3KZA, for example, is more than 6 Å. In some fish species and the paralogs p63/p73, a lysine is found at this position. The Arg290 side chain does not interact with the rest of the DBD and is generally disordered in DBD crystal structures, as for example, in PDB entries 2XWR [27] and 3KZ8 [28]. This residue is not conserved across vertebrate p53; a histidine is found at this position in beluga whale and feline p53.

Variant p.T312S was located in the adjacent flexible linker between the DBD and the tetramerization domain. Only one variant, p.E339K, was located in the tetramerization domain itself. Glu339 sits on the surface of the tetramer, facing the solvent, and is not involved in any inter-subunit contacts stabilizing the tetramer (Fig. 5C). Therefore, no major effects on the oligomerization equilibrium of p53 were expected for the lysine variant. This variant is, for example, also found in hamsters.

The remaining three variants affected the C-terminal regulatory region, an intrinsically disordered promiscuous binding module that can adopt different structural motifs, depending on posttranslational modification patterns and binding partners [22, 29]. Interestingly, one of those variants, p.S366A, removed a C-terminal phosphorylation site. Phosphorylation of Ser366 (and other serines in this region) by CHK1 and CHK2 modulates p53 C-terminal acetylation and the transactivation of p53 target genes [30].

### ACMG classification of *TP53* variants

**Fig. 6:**
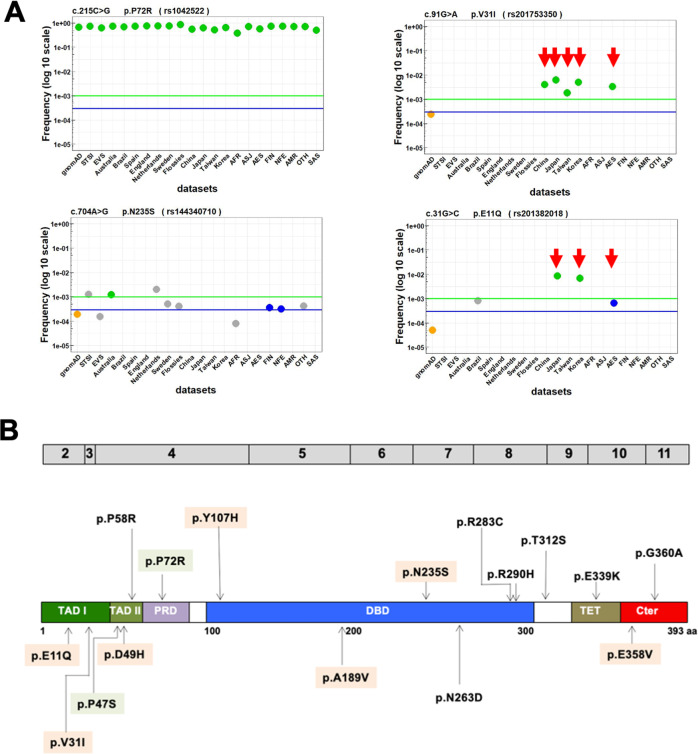
New *TP53* SNPs are spread out in the human population. **A** For the 14 population datasets used in this study as well as for the eight population-specific subsets of gnomAD, the frequency of each *TP53* SNP is shown as a colored dot: green: BA1 variants (AF ≥ 0.001 and AC ≥ 5); blue: BS1 variants (AF ≥ 0.0003 and AC ≥ 5); orange: variants with an AC ≥ 5 but falling short of the BA1 or BS1 allele frequency limit of 0.001 (green line) or 0.0003 (blue line); gray: variants with low AF and AC. A specific clustering in the Asian population can be distinguished for variants rs201753350 (p.V31I) and rs201382018 (p.E11Q) (red arrows). An extended analysis for all *TP53* variants is shown in Fig. S11. **B** Distribution of novel benign *TP53* variants analyzed in this study in the *TP53* protein. Green: benign *TP53* variants as defined previously. Orange: benign *TP53* variants identified in this study. Variants specific to an ethnic group are shown below the *TP53* protein. TAD I transactivation domain I, TAD II transactivation domain II, PRD proline-rich domain, DBD DNA-binding domain, TET oligomerization domain, Cter carboxy-terminal region. Only variants found in four or more different *TP53* datasets are shown. *TP53* exons 2–11 are shown in gray above the protein.

Variant classification according to guidelines proposed by the American College of Medical Genetics and Genomics (ACMG) and the Association for Molecular Pathology are very useful for clinicians assessing genetic analyses [31]. However, the classification of *TP53* variants according to these guidelines remains highly controversial. In the wake of heated debates, several ACMG-derived criteria have been suggested (Table S5) [32, 33]. Furthermore, *TP53* variant classification according to the ClinVar platform shows that only a single *TP53* variant (p.P72R) has been classified benign with three stars, whereas the entries for other variants have remained controversial (Table S5). This debate is partially due to the fact that the ACMG criteria were initially defined for Mendelian disorders in a specific clinical context. Also, they include many parameters that are not suitable for cancer. Furthermore, the prediction software, as well as population criteria used for an analysis, can influence the various scores. As *TP53* variants may be somatic or germline, the ACMG guidelines must be adapted to ensure accurate classification. Allele frequency in the human population is a strong criterion to define a variant as benign. A minor allele frequency (MAF) threshold of 0.001 and minimal allele count (AC) of five in the population are currently used to define the stand-alone BA1 criterion, which is sufficient to classify a variant as benign. In the present study, using the three aggregated datasets, only rs1042522 (p.P72R) could be ranked as BA1 (Table 1 and Fig. 6A). One of the main issues with an aggregated database including multiple ethnic populations is the dilution of the signals for variants specific to an ethnic group. Furthermore, these databases are skewed toward European ancestry (more than 50% for gnomAD), an aspect that can bias analyses. Using the aforementioned criteria, we analyzed the *TP53* variants using the eight population subsets from gnomAD and identified only two new BA1 variants, i.e., p.V31I in the Asian population (AF = 0.003 and AC = 65) and p.Y107H in the African population (AF = 0.00116 and AC = 23). The analysis of the 11 national datasets was far more informative, leading finally to the identification of nine *TP53* variants that should be defined as benign using ACMG criteria (Tables 1, S5 and Figs. 6A,  S11). Among those nine variants were the five EAS-specific SNP variants described above. Similarly, the analysis for the strong BS1 criterion (MAF ≥ 0.0003 and AC ≥ 5) with the whole-gnomAD database did not uncover new variants, whereas that of the various datasets led to BS1 classification for eight variants (Tables 1, S5 and Figs. 6A,  S11). BS1 classification requires a supporting criterion, such as BS3 defined by the loss of activity using “well established in vitro or in vivo assays”. Combining these criteria based on population frequency and multiple functional studies, including the present work, led to the classification of 16 of the set 21 variants as benign (Tables 1, S5 and Figs. 6A,  S11). For the remaining six *TP53* variants, other ACMG criteria were too heterogeneous for a clear-cut classification. We thus defined them as variants of uncertain significance (VUS).

## Discussion

Until recently, human genetic variation studies were largely restricted to constitutional variants occurring at frequencies above roughly 1–5%. That threshold was due to not only technological and cost limitations but also our own lack of knowledge of human genome diversity. Consequently, most medical genetic analyses were developed based on information derived from these common variants. Today, high-throughput DNA sequencing technologies enable the analysis of thousands of samples and the identification of infrequent SNPs. Recent explosive population growth has led to a great number of rare SNPs occurring at frequencies below 0.5% [34, 35]. These low-frequency variants are rich in protein-coding sequences that could predispose to disease. For genes with high clinical impact, it is therefore essential to (i) identify all relevant variants present in the human population and (ii) define their potential pathogenicity.

The *TP53* gene is a paradigm for such analyses. Indeed, the accurate diagnosis of somatic mutations in it is mandatory in multiple clinical settings, for example in chronic lymphocytic leukemia, where it is used to improve patient stratification and optimize therapeutic decisions [2]. Furthermore, the diagnosis of germline *TP53* mutations for genetic counseling is proving pertinent in such pathologies as hereditary breast and ovarian cancer syndrome and pediatric cancer [1, 36].

Recently, the ACMG—Association for Molecular Pathology provided a systematic method for the interpretation of sequence variants for genetic disorders [31]. Unfortunately, a high fraction of variants is relegated to uncertain significance (VUS), often due to the rarity and conflictual nature of results in existing databases and the primary literature. Several recent publications sought to improve the classification of *TP53* variants but their results were heterogeneous and thus a source of discord.

Historically, two exonic missense *TP53* SNPs have been identified and defined as benign constitutional variants [37]. Somatic *TP53* gene mutations have been described in more than 100,000 patients. However, in most of these cases, matched normal DNA was not available and, thus, the somatic origin of the mutation was determined by consulting reference databases. It is therefore highly likely that some of those variants were infrequent constitutional variants mistakenly identified as somatic mutations.

In the present study, using a combination of in silico, in vitro, structural, and genetic approaches, we addressed a two-tiered question: (i) is it possible to identify novel and infrequent *TP53* SNPs in the human population and (ii) what is the potential pathogenicity of these variants?

The use of multiple independent population databases has proven very helpful for the selection of novel potential SNPs. Although gnomAD is an outstanding resource for population analyses, the addition of novel datasets of individuals from different ethnic groups has led to a more accurate selection of potential SNPs [38].

In silico predictive software enables the assessment of the effects of amino acid substitutions on protein structure or function with, in principle, no imperative need for functional studies. However, in silico and functional approaches each have their own strengths and weaknesses. Depending on the algorithm, the performance of in silico approaches will vary based on the specific gene and protein being considered. As all algorithms focus on protein function based on the homology of sequences and/or the physical properties of amino acids, confusion has arisen between deleteriousness and pathogenicity, which should be considered as two different features. Great caution has been advised for the use of these algorithms, including for *TP53* [39–41]. Concerning this latter, mutations in the mdm2 binding domain, localized in the amino-terminus of the protein, are predicted to be deleterious and consequently classified as pathogenic. In fact, these variants are known to impair cell growth and ultimately lead to cell death, which is the opposite of what would be expected for cell transformation. This aspect is supported by the total absence of cancer-associated mutation in the two highly conserved codons 19 and 23 of this *TP53* protein domain [42].

One major advantage for *TP53* is the availability of three unbiased large-scale functional studies for all potent *TP53* missense variants [16–18]. A correlation analysis showed excellent agreement between the three datasets despite their different readouts [43]. Although predicted as “nonfunctional” by several algorithms, variants such as p.N235S or p.E11Q do not display loss of activity. This confusion has led to a controversial classification of several *TP53* variants. p.N235S has been defined as pathogenic, likely pathogenic or VUS in different studies [32, 44–46]. It remains defined as pathogenic in the COSMIC database (version 92) despite the fact that it is considered as likely benign in Clinvar since spring 2020 and was found to be thus in the present study as well. For *TP53*, we strongly advocate for the use of functional studies to assess *TP53* variant deleteriousness.

The consequences of *TP53* mutation on protein function and their repercussions on the various pathways regulated by *TP53* are highly heterogeneous. Although a few variants can be considered as amorph with a total loss of function, the complex structure of the *TP53* protein is known to give rise to variants with multiple consequences with hypomorph, neomorph or antimorph properties [47–49]. *TP53* regulates transcription through specific binding to highly degenerated response elements in promoters or introns of target genes [50]. The affinity of *TP53* for these various biological sites is variable, and several *TP53* variants can display only partial loss of their DNA-binding activity, allowing the mutant protein to bind only to a subset of *TP53* response elements. This feature is linked to differential transactivation activities that may depend on p53 protein levels, as well as target sequences [51, 52]. This heterogeneity for both wild-type and mutant *TP53* is reflected by our transcriptional analysis performed on endogenous genes (Fig. S7). Genes activated by wild-type *TP53* have been extensively analyzed and scrutinized in normal and tumor cells. However, recent studies have shown that genes repressed by *TP53* are also key players in the tumor-suppressive effect of *TP53*. Within this latter aspect is the downregulation of cell-cycle genes via the p53–WAF1–DREAM–E2F/CHR pathway (p53–DREAM pathway). Upon p53 induction, p21^WAF1/CIP1^ activates the transcriptional repressor DREAM, leading to the shutdown of more than 200 cell-cycle genes [53, 54]. Derepression of these genes has been shown to be of better prognostic value than *TP53* mutations [42]. Whether or not the loss of function of *TP53* variants toward the WAF1 promoter and the DREAM pathway is essential for promoting tumorigenicity remains to be elucidated. As of this writing, there is no “gold standard” assay to define a *TP53*, suppressive effect, but the observation of defective transcription in the large majority of *TP53* variants found in human cancer indicates that this activity is a key target. Although none of the variants in the present study displayed any obvious functional defects, we cannot exclude that any one of them may be partially defective for a particular function. Nonetheless, their high frequency in the human population and low frequency in cancer mutation databases suggest that any deleterious clinical impacts will be very low.

Using different functional assays, we showed that a subset of 21 germline *TP53* variants found recurrently in multiple population datasets from different ethnic groups was indistinguishable from wild-type *TP53*. Through the analysis of multiple national datasets, we were able to definitively validate nine of the 21 variants as BA1, i.e., the ACMG stand-alone criterion for benignity. Seven other variants met the ACMG criterion BS1 + BS3, also indicating benignity. Thus, we identified 16 variants that may only be defined as natural polymorphisms. Among those variants, we identified for the first time five variants that were specific to the Asian population (present only at very low frequency in other populations). Our analysis of the UMD_*TP53* database confirmed the Asian origin of the five variants and furthermore showed that they were mistakenly defined as somatic pathogenic mutations. We also identified another *TP53* variant specific to the Indian population. Our analysis emphasizes the need to exercise great caution when using population datasets to check patient cohorts. Matching the ethnicity of the patient or the cohort to the reference datasets appears essential for increasing the accuracy of the analysis [55]. Also, supplementing these datasets with data from normal populations and from all geographical areas is necessary to minimize variant misclassification. For example, the Asian variant rs201753350 (p.V31I), classified as benign (BA1) in our analysis using multiple criteria, had been classified as pathogenic in a family with gastric cancer in the Japanese population [56]. Such “false positive” results could have detrimental consequences for patients and families, who may end up experiencing anxiety, undergoing unnecessary examinations, etc.

We cannot exclude that a number of the variants described in the present study may be associated with specific hereditary traits associated or not with cancer, or with other genetic characteristics. To illustrate this point, variant rs1042522 (p.P72R) does not impair *TP53* activity but whether it is associated with an increased risk of cancer remains highly controversial [23]. Similarly, variant rs1800371 (p.P47S), restricted to the African population, has been shown to be associated with an increased risk of breast cancer [57].

Most of the variants described in set 21 are described as pathogenic or likely pathogenic in multiple databases and pipelines for the assessment of pathogenicity. To improve the accuracy of *TP53* mutation diagnostics, they should be updated with the present data. The 2020 release of the UMD_*TP53* database as well as the web application Seshat have been updated with these novel data.

This analysis was made possible by the numerous functional and structural analyses available for *TP53*. Unfortunately, the quantity of research for other cancer genes is much more limited. Nevertheless, the availability of large, independent datasets representing ethnic populations should be sufficient to identify novel benign variants and emphasize genetic heterogeneity. For an accurate assessment of somatic and germline variants, human genome reference databases used in analytical pipelines must include accurate ethnicity information to ensure that genetic analyses provide the most pertinent information possible for patients.

## Materials and methods

Details on the cell lines, western blot, luciferase assay, plasmid construction, and flow cytometry analysis for the experimental validation of *TP53* variants identified in this study are provided in the Supplementary Information.

### Population datasets

*TP53* variants from a range of population databases were downloaded from their respective portals (Table S1). The origin of each dataset was carefully checked to avoid redundancies. Because a part of the 1000 Genomes Project was included in gnomAD, the data of the former were not used in the present study. To ensure accurate comparisons between the various datasets, data from all databases were obtained using the genomic coordinates of the entire *TP53* gene as defined by RefSeq. In a first step, minimal genomic data, such as genomic coordinates and genetic events, were extracted from each dataset to define the correct annotation according to HGVS recommendations. In a second step, variant annotation was validated by using the Name Checker tool developed by Mutalyzer (https://mutalyzer.nl/). This latter handles all types of *TP53* gene variations, such as substitutions, insertions, duplications, deletions, and more complex insertion/deletions [58]. The current version (2.0.26) of Mutalyzer uses the stable NCBI sequence NG_017013.2 as reference for *TP53*. This is an essential issue for avoiding problems due to the use of multiple genome references (NCBI Build 36.1/hg18, Genome Reference Consortium GRCh37/hg19 or Genome Reference Consortium GRCh38/hg38) by the various NGS pipelines as well as noncompliant nomenclature. To minimize database contamination by potential carriers of germline *TP53* variants, the non-cancer version of gnomAD was used in the present analysis. Taken together, exonic *TP53* variants issued from gnomAD correspond to 56% of the variants issued from the 14 cohorts.

Cancer-associated *TP53* variants were downloaded from the UMD_*TP53* database (Released 2017), which includes 80,402 mutations (6870 different *TP53* variants) identified in tumors, in cell lines (somatic mutations) or in patients with hereditary cancers (germline mutations) [37]. This release includes mutations identified by conventional sequencing or by NGS (Table S1).

The sequence nomenclature used for *TP53* variants in this work is in accordance with the Human Genome Variation Society’s guidelines using the NM_000546.5 transcript sequence and the full-length protein NP_000537.3 [59].

We previously defined and validated a specific set of pathogenic *TP53* variants called the “CSD” [15]. Briefly, *TP53* variants were extracted from four different nonoverlapping datasets on various types of cancer. The Sanger dataset includes 4299 variants extracted from the latest version of the UMD_*TP53* mutation database excluding all NGS studies (Leroy et al. [37]). The other three sets of *TP53* variants were compiled from large independent cancer sequencing projects: TCGA [60], ICGC [61], and MSK-IMPACT [62]. Each of these studies described more than 2000 different *TP53* variants occurring at various frequencies in the coding regions or splice sites (+2/−2) of *TP53*. We created the CSD by combining these four datasets to define a core of 471 recurrent *TP53* variants found at least once in each database [15]. The four datasets resulted from independent studies using different patients and different methodologies. We showed that shared variants were true recurrent pathogenic variants [15].

### Functional analysis from large-scale mutagenesis data

The UMD_*TP53* database includes three sets of functional data for *TP53* variants. The first set was described in detail in a previous report [16, 63]. Briefly, haploid yeast transformants containing 2314 *TP53* variants and a green fluorescent protein reporter plasmid were constructed. *TP53* activity was tested by measuring the fluorescent intensity of green fluorescent protein that is controlled by eight different promoter sequences regulated by the p53 protein after 3 days of growth at 37 °C. The second set of functional data integrated in the UMD_*TP53* database corresponds to the analysis of 5300 *TP53* variants performed by Kotler et al. [17]. The growth activity of all variants localized in the DBD of *TP53* was assessed in H1299 cells. The third set corresponds to the study by Giacomelli et al. [18]. In this study, dominant negative activities, losses of function, and responses to etoposide were analyzed in mammalian cells for 8258 *TP53* variants (Table S4). Functional scores were extracted from these 12 datasets associated with the three studies and normalized for all the analyses of *TP53* variants.

### Selection of *TP53* variants for in silico and in vitro analysis

A subset of 27 *TP53* variants (set 27) were chosen for in silico analyses based on their occurrence in multiple datasets (more than 2). We also added the five Asian-specific variants and eight of the ten outlier germline variants found in the UMD_*TP53* database. The variant p.T125R, found at high frequency as a germline variant in the database, was excluded because missense and synonymous mutations in this codon close to a splice site are known to impair *TP53* splicing [64, 65]. The pathogenic variant p.R337H (known as the Brazilian mutation) associated with adrenocortical carcinoma was also excluded [66].

Set 21, used for in vitro analyses, is derived from set 27 using more stringent criteria. It includes variants found in multiple datasets (more than 4) at frequencies higher than 0.0005. Asian and outlier germline variants were retained but variants p.R248Q, p.R283H, and p.R156H found, respectively, in five, four, and four datasets were not included as they are well-known pathogenic variants included in the CSD. Set 21 thus included 19 novel *TP53* variants as well as rs1042522 (p.P72R) and rs1800371 (p.P47S).

### Molecular modeling and stability predictions

Models of p53 DBD mutants were generated using the SWISS-MODEL server [67], with PDB entry 2XWR as a template [27]. Differences in the melting temperatures of the mutant proteins compared with the wild-type protein were predicted using the HoTMuSiC server [68] based on PDB entry 2XWR and a wild-type DBD melting temperature of 46 °C.

### Variant effect prediction

dbNSFP v3.5 was downloaded from the Jpopgen website (https://sites.google.com/site/jpopgen/dbNSFP). This database compiles prediction or conservation scores from multiple prediction algorithms [12]. Prediction scores from each algorithm were available as normalized data from 0 (less deleterious) to 1 (most deleterious).

Five other predictors not included in dbNSFP were also used: Revel [69], PON-P2 [70], MutPred2 [71], Mut_Pred [72], and Envision [73]. Brief descriptions of these predictors are provided in Table S4.

### Literature survey and ethnicity analysis

The 2017 release of the UMD_*TP53* database includes data from 4600 publications. A manual survey of the database was performed for the five *TP53* variants suspected to be constitutional variants specific to the Asian population. Publications describing any one of these variants were retrieved for analysis. In most cases, the hospitals and/or institutions used for patient recruitment were available.

## Supplementary information

Supplementary information

Supplementary Figures and Tables

Table S2

Table S3

Table S5
