# Supplementary material for: Identification and functional characterization of new missense SNPs in the coding region of the TP53 gene
Source: Cell Death Differ. 2020 Nov 30;28(5):1477–92. doi: 10.1038/s41418-020-00672-0 (PMC8166836; doi:10.1038/s41418-020-00672-0)
Supplement: Supplementary file 1 — Supplementary information [file 41418_2020_672_MOESM1_ESM.pdf]

Supplementary Information

Identification and functional characterization of new missense SNPs in the coding region of the *TP53* gene

Flora Doffe, Vincent Carbonnier, Manon Tissier, Bernard Leroy, Isabelle Martins, Johanna S.M. Mattsson, Patrick Micke, Sarka Pavlova, Sarka Pospisilova, Jana Smardova, Andreas C. Joerger, Klas G. Wiman, Guido Kroemer and Thierry Soussi

Corresponding author: Thierry Soussi
Email: thierry.soussi@sorbonne-universite.fr

This PDF file includes:

Supplementary methods
- Cells
- Plasmid construction
- FASAY
- Cell growth suppression
- Luciferase assay
- Real time PCR assay
- Flow cytometry
- Molecular modelling and stability predictions
- Variant effect prediction

Extended analysis
- Non-coding TP53 variants
- Synonymous TP53 variants
- TP53 variants in exons 9_beta and 9_gamma
- p.R175C, a frequent passenger mutation in human cancer

References

Supplementary Methods

Cells

H1299 cells were obtained from the ATCC and maintained in Dulbecco's modified Eagle's medium with glucose (4.5 g/l), L-glutamine, and sodium pyruvate. The medium was supplemented with 10% heat-inactivated fetal calf serum and antibiotics. Cells were grown at 37 °C in a humidified 5% CO_2 atmosphere. This cell line is devoid of endogenous *TP53* due to a large deletion of the gene.

Plasmid construction

All *TP53* variants were expressed using the mammalian expression vector pcDNA3.1 neo. cDNA *TP53* variants were synthesized by GenScript (Piscataway, NJ) and verified by sequencing. Luciferase reporter vectors have been previously described and include either the natural promoters of these genes (WAF1 and HDM2) or a consensus response element CONS [1, 2].

FASAY

FASAY was performed as previously described [3, 4] with small modifications. PCR was performed using 1 µl cDNA, primers P3 (5'-CCT TGC CGT CCC AAG CAA TGG ATG AT-3') and P4 (5'-ACC CTT TTT GGA CTT CAG GTG GCT GGA GT-3'), and Phusion DNA polymerase (New England BioLabs). YPH-p21yeast cells containing the p53-specific RE derived from the regulatory regions of the WAF1 gene [5] were co-transformed with the PCR product, linearized pSS16 plasmid and salmon sperm DNA as a carrier (Life Technologies, Inc., Carlsbad, CA, USA) using the lithium acetate procedure (Ishioka et al. 1993). Transformed yeast cells were plated on minimal medium lacking leucine and containing adenine (5 µg/ml), followed by incubation at 25, 30 and 35 ˚C for four days. Relative transactivation by *TP53* was assessed semi-quantitatively according to the color of the resulting yeast colonies using an empirically-established seven-step color scale [4].

Cell growth suppression

Colony formation assay was performed as previously described [1]. H1299 cells were plated into six-well plates and transfected on the following day with Lipofectamine 2000 (Life Science). Twenty-four hours after transfection, the cells were dissociated and plated at a density of 5,103 cells per well in six-well plates in selective media with G418 at a concentration of 1 mg/ml. Cells were then stained with crystal violet after 14 to 16 days.

Luciferase assay

H1299 cells were plated in 96-well plates (2,000 cells per well). After 48 hours, the cells were transfected using Lipofectamine 2000 (Life Science). Seventy-five ng of reporter gene

and 5 ng of p53 plasmid were used for each well. The luciferase activity was tested 24-48 hours after transfection. Each assay was performed in triplicate and all *TP53* variants were tested at least three times with MDM2, WAF1 or a synthetic promoter (CONS) in separate experiments [2].

Real time PCR assay

TaqMan Low Density Arrays (384 well, Applied Biosystems) were custom designed to comprise genes involved in the various pathways of *TP53*. H1299 cells were plated in 6-well plates (150,000 cells per well). After 24 hours, the cells were transfected using lipofectamine 2000 (Life Science) with 1 µg of *TP53* expression vector. An empty vector (pcDNAneo) was used in all experiments as a negative control. Total RNA was isolated 24 hours post transfection using the Rneasy Mini kit (Qiagen) according to the manufacturer's protocol. Nine hundred nanograms of RNA were converted to cDNA using Superscript III First-Strand Synthesis SuperMix for qRT-PCR (Invitrogen, Carlsbad, CA, USA). Two microliters of cDNA were mixed with TaqMan Universal PCR Master Mix (p/n 4,304,437, Applied Biosystems (ABI) Foster City, CA, USA) and RNase-free water to obtain a final concentration of 2 ng/µl. The cDNA mix was loaded into each of the eight loading ports of a Taqman low density array (ABI Foster City, CA, USA). The array was sealed and centrifuged for two minutes at 1200 rpm and thereafter loaded on a 7900HT qPCR machine (ABI, Singapore) with ABI software SDS v2.4 installed. PCR cycling was standard TLDA array cycling. For ΔΔCq calculations, ABI software RQmgr 1.2.1 followed by DataAssist v3.0 were used. GAPDH was used as the reference gene.

Flow cytometry

H1299 cells were plated into six-well plates and transfected on the following day with Lipofectamine 2000 (Life Science) with GFP and TP53 expression vectors (ratio 2/3-1/3). Twenty to forty-eight hours after transfection, the cells were washed twice with cold PBS and suspended in 1× binding buffer supplemented with APC Annexin V (BD Biosciences) and 4',6-diaminidino-2-phenylindole (DAPI, Molecular Probes) (5 µg/mL) according to the manufacturer's instructions. Samples were then analyzed using a MACSQuant10 cytometer (Miltenyi Biotec) and the data statistically evaluated using Flowjo v10.2 software.

Immunoblotting

Immunoblotting was performed following standard procedures. Cells were harvested and the obtained pellet was resuspended in RIPA buffer (89900; Thermo Fisher Scientific) supplemented with phosphatase and protease inhibitors (R0278; Sigma-Aldrich). Protein content quantification was done using the DCTM Protein Assay kit (5000112; Bio-Rad, Hercules, CA, USA). Protein were separated on NuPAGE Novex Bis-Tris 4–12% pre-cast gels (Invitrogen-Life Technologies, Carlsbad, CA, USA) and transferred to Immobilon polyvinylidene difluoride membranes (Merck-Millipore, Darmstadt, Germany). Unspecific binding was reduced by incubating the membranes for 1 hour in 0.05% Tween 20 (v/v in TBS) supplemented with 5% w/v bovine serum albumin (Euromedex, Souffelweyersheim,

France). Thereafter, proteins were probed with antibodies specific for TP53 (DO7) or GFP. Primary antibodies were revealed with species-specific immunoglobulin G conjugated to horseradish peroxidase (Southern Biotech, Birmingham, AL, USA), followed by chemiluminescence analysis with the SuperSignal West Pico reagent by means of an ImageQuant 4000 (GE Healthcare, Little Chalfont, UK).

Molecular modelling and stability predictions

Models of p53 DBD mutants were generated using the SWISS-MODEL server [6], with PDB entry 2XWR as a template [7]. Differences in the melting temperatures of the mutant proteins compared to the wild-type protein were predicted using the HoTMuSiC server [8] based on PDB entry 2XWR and a wild-type DBD melting temperature of 46 ºC

Variant effect prediction

Many *in silico* predictive scores used in the literature, such as SIFT, PolyPhen or PROVEAN, predict whether an amino acid substitution **affects protein function** based on sequence homology and/or the physical properties of amino acids. The predictive score of these algorithms are still widely used in clinical pipelines to assess the consequence of cancer-associated variants. Very unfortunately, **protein function** is misused for **pathogenicity** based on the false assumption that a loss of function in a protein will always be associated with disease. As recommended by the ACMG, these tools should only be used as a criterion among others that define variant pathogenicity (criteria PP3/BP4).

Extended analysis

Intronic and non-coding TP53 variants

We identified 2,909 intronic TP53 variants in the examined datasets **(Supplementary Table. S3a).** Most of the variants were found in only a small number of them, but 127 were recurrently identified in five or more datasets **(Fig. 1)**.

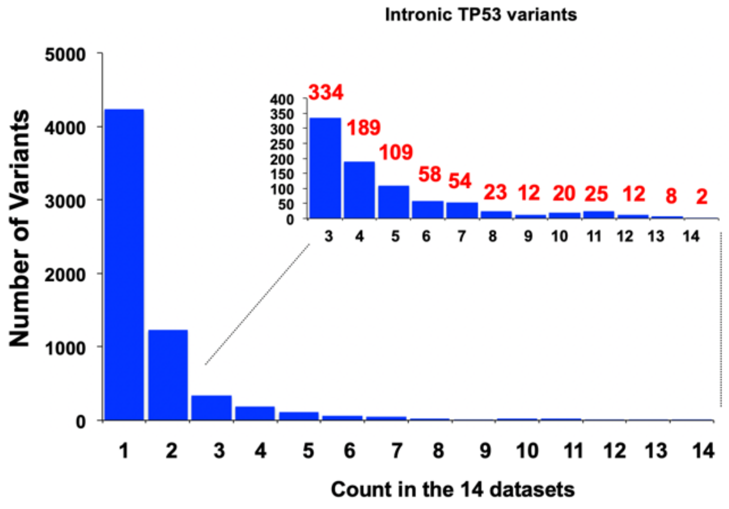

Fig. 1: Occurrence of intronic *TP53* variants in the 14 datasets
As several studies (e.g. FLOSSIES) include only exonic data, the occurrences for a few SNPs are underestimated.

Of the 127 recurrent variants, 37 (29%) were found with both a frequency higher than 0.1 and a high allele count, classifying them as BS1 (benign) according to ACMG criteria **(Fig. 2a)**. Although many variants occurred infrequently and therefore could not be classified according to the ACMG rules, their recurrent observation in independent datasets was highly suggestive of population variants (SI Appendix, Fig. S13B). As for exonic variants, we found that several of them were either specific to the Asian population or specifically absent from it **(Fig. 2b)**.

We identified 220 *TP53* variants in the 5' or 3' UTR **(Supplementary Table. S3a).** Variant rs78378222 was of particular interest as it was located in the polyadenylation signal sequence of TP53. The A-to-C transition changed AATAAA to AATACA, impairing TP53 3′-end processing and thereby decreasing TP53 expression levels. Several studies have shown that rs78378222 increases susceptibility to several cancers [9, 10]. This variant was found neither in the two Asian population subsets of gnomAD nor in the four Asian population datasets **(Fig. 2b)**. Our observation could explain some of the disparity in the various GWAS analyses observed with rs78378222 [10].

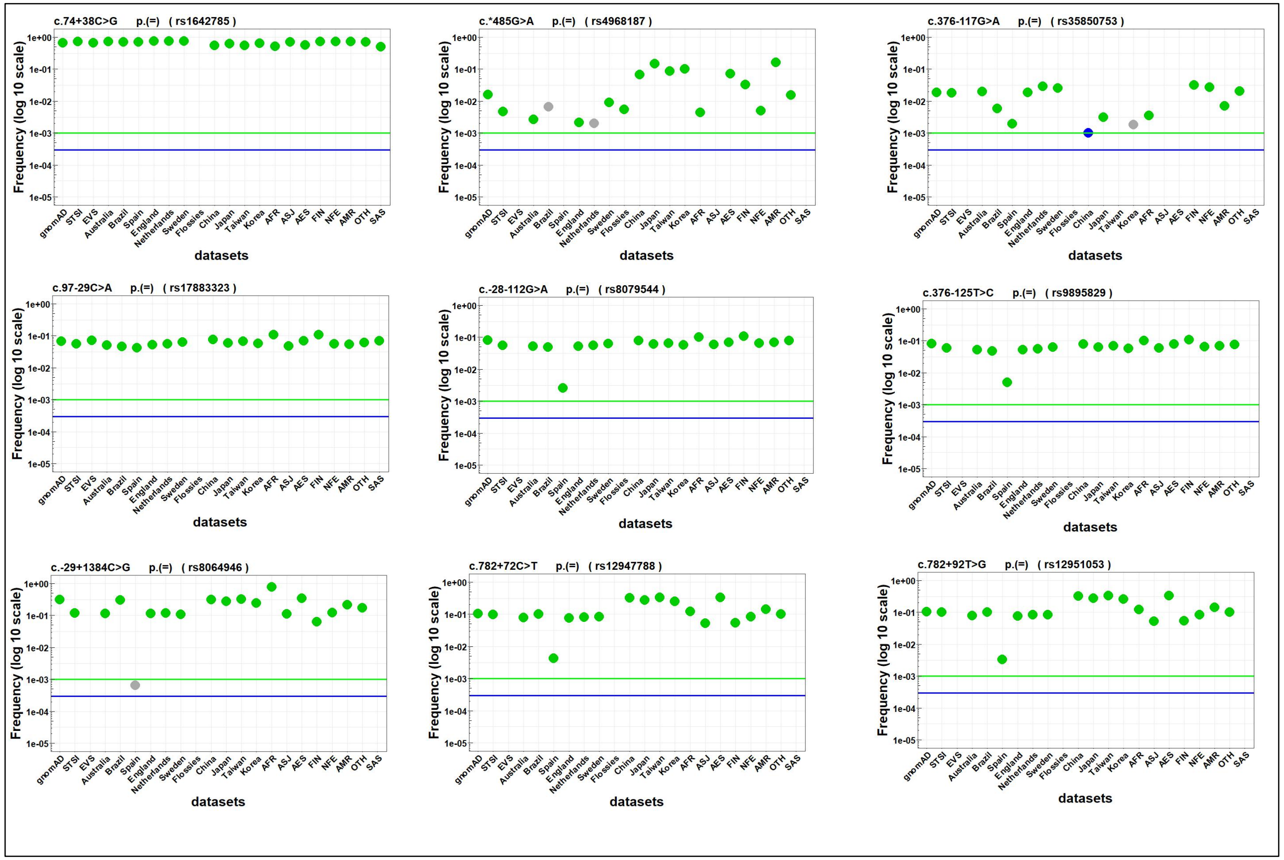

Fig. 2 (part 1) Allele frequency distribution of intronic *TP53* variants in the included datasets and classification according to ACMG criteria

For the 15 population datasets used in this study and the eight population-specific subsets of gnomAD, the frequency of each cp*TP53* variant is shown as a colored dot: green: BA1 variants (AF ≥0.001 and AC ≥5); blue: BS1 variants (AF ≥0.0003 and AC ≥5); orange: variants with an allele count ≥5 but falling short of the BA1 or BS1 allele frequency limits of respectively 0.001 (green line) or 0.0003 (blue line); grey: variants with low AF and AC.

a: Frequent intronic *TP53* variants.

b. *TP53* variants specific to the Asian or non-Asian populations.Arrows indicate the six subsets of the human population that include the Asian population (red: variants absent from the Asian population; blue: variants specific for the Asian population). Variant rs78378222, associated with *TP53* RNA instability, is indicated with a red border.

c: Low-frequency but recurrent *TP53* variants

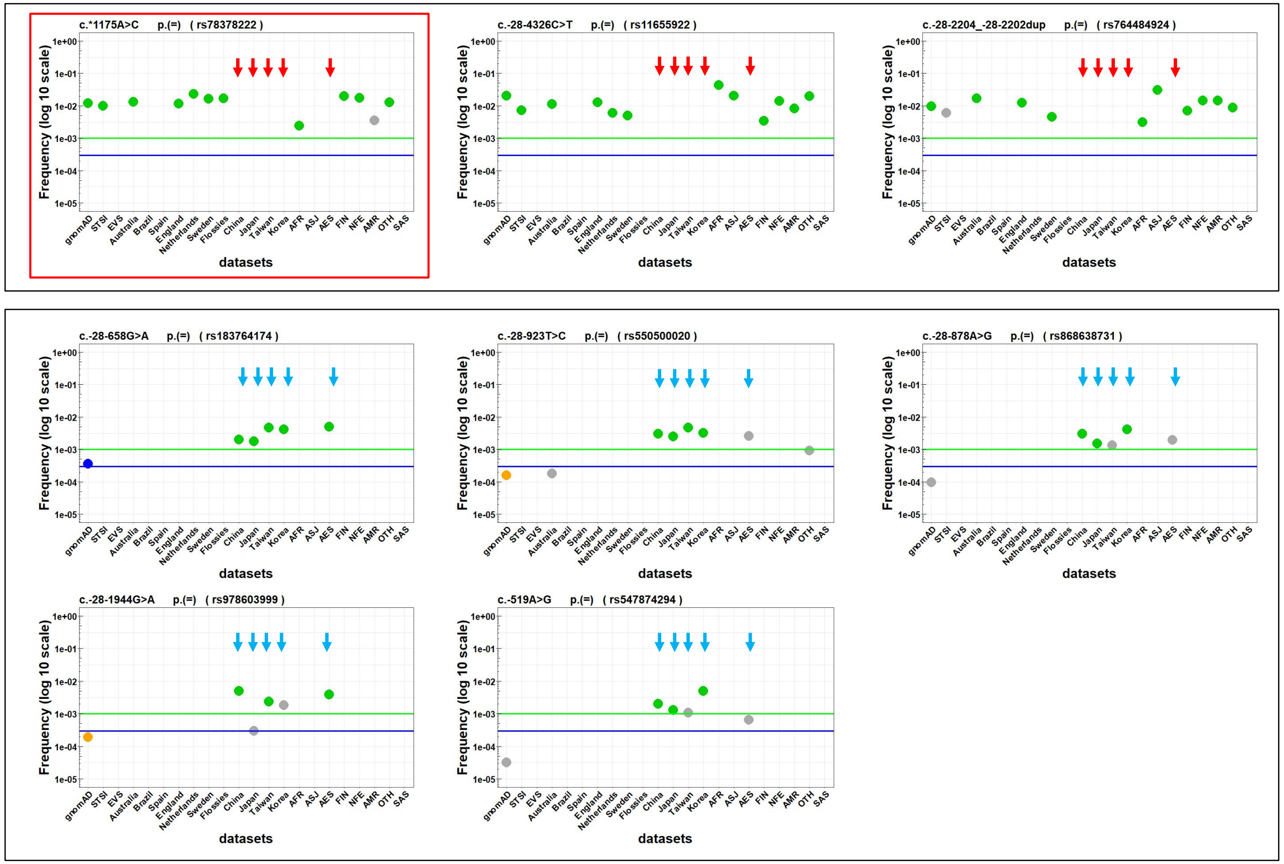

Fig. 2 (part 2) Allele frequency distribution of intronic *TP53* variants in the included datasets and classification according to ACMG criteria

See legend of Fig. 2 (part 1) for further details.

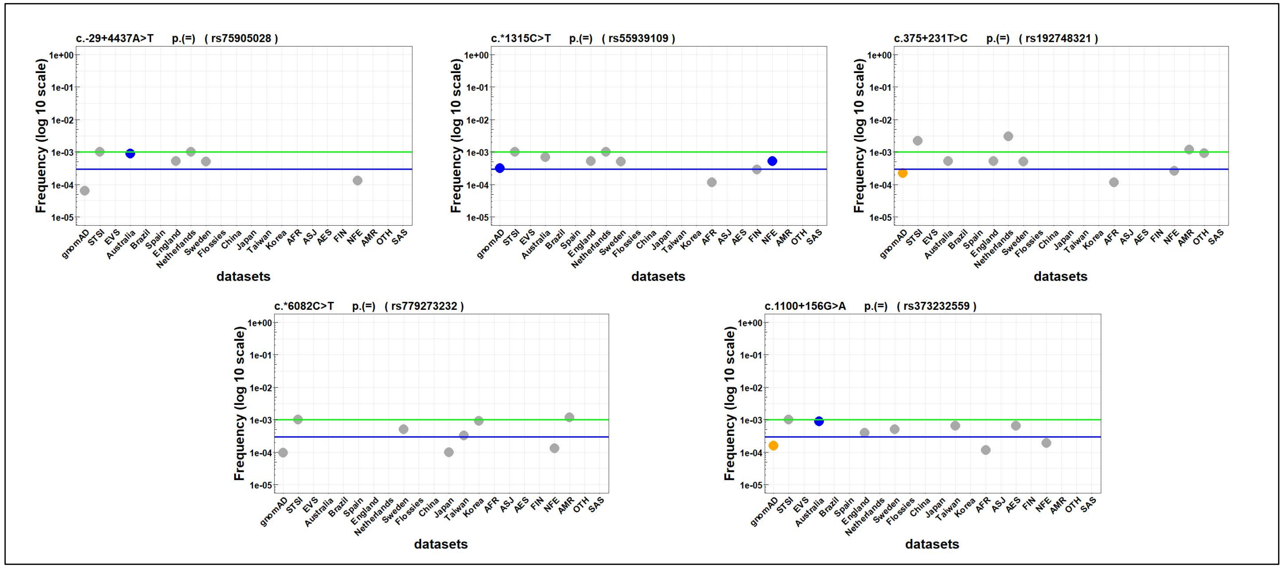

Fig. 2 (part 3) Allele frequency distribution of intronic *TP53* variants in the included datasets and classification according to ACMG criteria

See legend of Fig. 2 (part 1) for further details.

.

TP53 synonymous variants

Among the exonic variants in the *TP53* coding region, we identified 112 synonymous variants, 19 of which were present in at least three different and independent datasets **(Supplementary Table S3a and Fig. 3a and b)**. Two of these variants, rs1800370 (p.P36=) (frequency 1.27 10^{-2} in gnomAD) and rs1800372 (p.R213=) (frequency 1.23 10^{-2} in gnomAD), identified respectively in 14 and 11 datasets, are well known non-pathogenic TP53 constitutional variants classified as benign by ClinVar with a 2-star ranking **(Fig. 4)**. Both variants can be classified as BS1 using ACMG classification.

The remaining variants are found at much lower frequencies (2.5×10^{-4} to 7×10^{-6}), but with recurrence in at least three different datasets, suggesting that they were low-frequency constitutional polymorphic variants. Their frequencies in the various population databases do not permit any classification according to ACMG criteria.

TP53 SNP rs1800369 (p.D21=) (frequency 5.3×10^{-4} in gnomAD), identified in 1990 and usually considered as a rare benign SNP, was identified in only one dataset [11]. Information from ClinVar for this variant is quite discordant. In 2015, it was classified as likely benign with two stars, but it is now labelled as "Conflicting interpretations of pathogenicity" with only a single star.

The remaining synonymous variants found in only one or two datasets occur at much lower frequencies (5×10^{-6} to 2×10^{-4}).

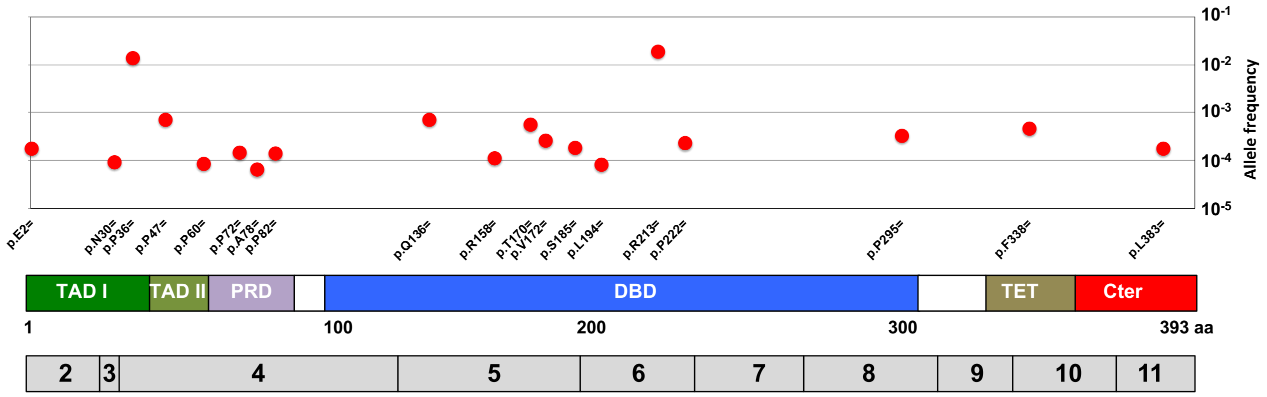

Fig. 3a: Distribution and frequency of synonymous variants analyzed in this study along the TP53 protein.
TAD: transactivation domain I; TAD II: transactivation domain II; PRD: proline-rich domain; DBD: DNA-binding domain; TET: oligomerization domain; Cter: carboxy-terminal region. *TP53* exons 2 to 11 are shown in grey below the protein.

gnomAD _RSID	cDNA_variant	Protein_Variant	Population_Occurence	gnomAD	Australia	Brazil	China	England	Flossies	Japan	Korea	Netherlands	Spain	Sweden	Taiwan	EVS	STSI
rs1800370	c.108G>A	p.P36=	14	0.012768	0.01564	0.01724	0.014	0.01415	0.0233	0.0045	0.00636	0.023	0.0186	0.013	0.00133	0.0113	0.01333
rs1800372	c.639A>G	p.R213=	11	0.012286	0.0174	0.02792		0.01653	0.0269	0.0003		0.026	0.03286	0.0045		0.0137	0.01778
rs150293825	c.1014C>T	p.F338=	7	0.000259	0.00035	0.00082		0.00026	0.0002							0.0002	0.001
rs72661118	c.666G>T	p.P222=	6	4.25E-05	0.00035			0.00026	0.00041	0.0001						0.0002	
rs201741778	c.141G>A	p.P47=	5	7.43E-05			0.001				0.00182		0.00031		0.00033		
rs372397095	c.246G>A	p.P82=	5	1.42E-05	0.00018				0.0001				0.00031			8E-05	
rs757544615	c.510G>A	p.T170=	4	9.9E-05		0.00082							0.00031				0.001
rs143458271	c.6G>A	p.E2=	4	8.87E-05	0.00018				0.0002							0.0002	
rs758781593	c.408A>G	p.Q136=	4	1.06E-05			0.002				0.00046				0.00033		
rs370216745	c.582T>C	p.L194=	4	7.95E-06				0.00013	0.0001							8E-05	
rs370992294	c.90C>T	p.N30=	4	7.12E-06	0.00018				0.0001							8E-05	
rs56275308	c.216C>T	p.P72=	3	8.16E-05					0.0002							0.0002	
rs139200646	c.474C>T	p.R158=	3	5.31E-05					0.0002							8E-05	
rs200073907	c.885T>C	p.P295=	3	4.6E-05		0.00082			0.0001								
rs373710656	c.1149C>T	p.L383=	3	3.58E-05					0.00041							8E-05	
rs749289195	c.180A>C	p.P60=	3	1.77E-05				0.00013	0.0001								
rs375099397	c.234A>G	p.A78=	3	1.6E-05					0.0001							8E-05	
rs749309577	c.516T>G	p.V172=	3	1.19E-05						0.0003	0.00046						
rs367560109	c.555C>T	p.S185=	3	1.06E-05							0.00046					8E-05	

Fig. 3b: Frequency of synonymous *TP53* variants in the range of datasets. (Only variants occurring in more than three datasets are shown).

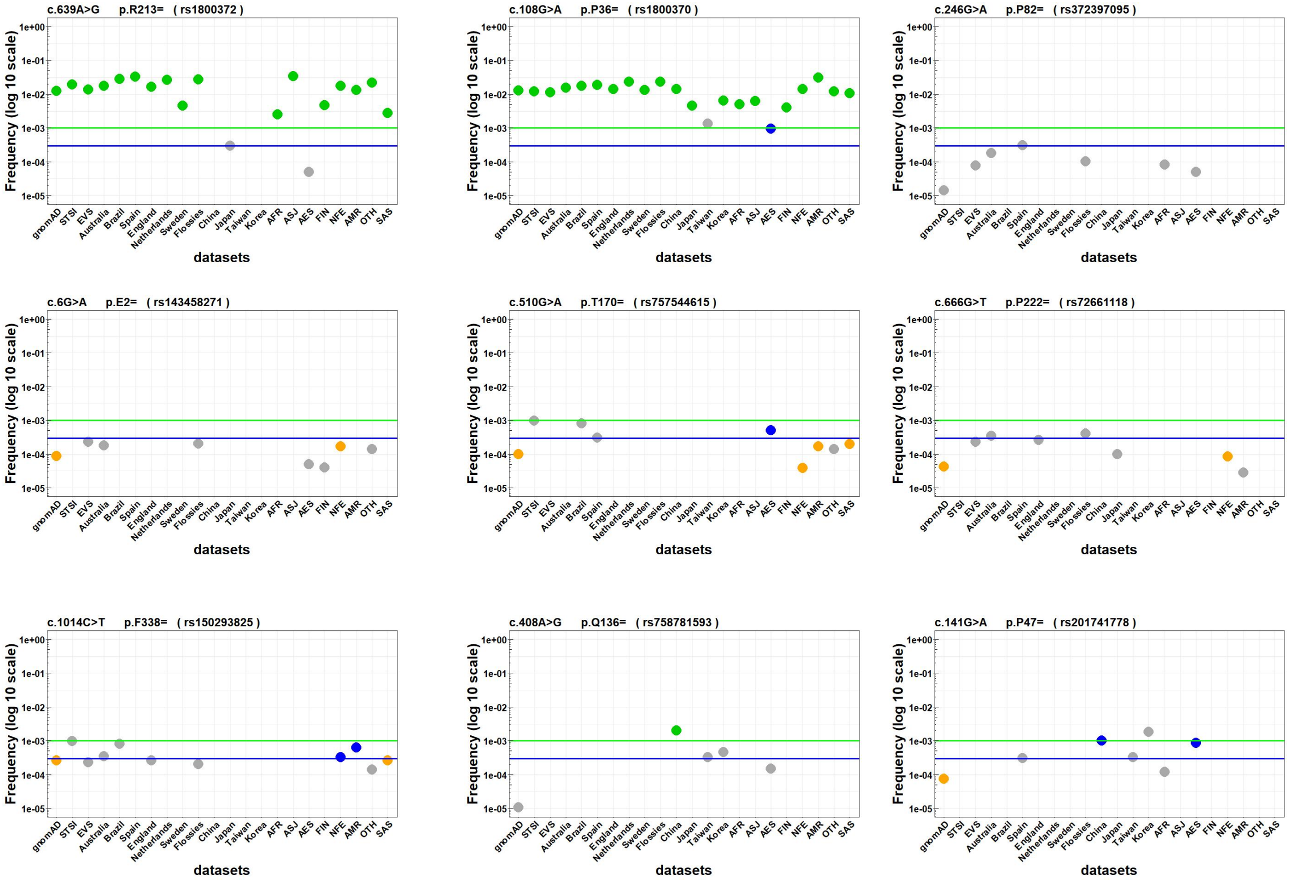

Fig. 4: Allele frequency distribution of synonymous *TP53* variants in the included datasets and classification according to ACMG criteria

For the 15 population datasets used in this study as well as the eight population-specific subsets of gnomAD, the frequency of each synonymous cp*TP53* variant is shown as a colored dot: green: BA1 variant (AF ≥0.001 and AC ≥5); blue: BS1 variants (AF ≥0.0003 and AC ≥5); orange: variants with an AC ≥5 but falling short of the BA1 or BS1 allele frequency limits of respectively 0.001 (green line) or 0.0003 (blue line); grey: variants with low AF and AC.

TP53 variants in exons 9_beta and 9_gamma

TP53 exons beta and gamma are localized in intron 9 and lead to the alternative expression of the minor splice forms beta and gamma with a different carboxy-terminus that lacks most of the tetramerization domain (**Fig. 5**). This location in intron 9 has not been extensively analyzed or included in exome sequencing strategies. It is therefore likely that potential variants in this region are underrepresented.

Nonetheless, eight and ten variants were detected in exon 9 beta and gamma respectively, but only three and five were found in three or more datasets (**Table 1**).
The missense variant c.993+223T>G (p.C341G for TP53 exon beta) is found only in the Asian population whereas the synonymous variant c.993+228A>G (p.Ter342Ter exon beta) is specific for the non-Finnish European population **(Table 1).**

The functional significance of these 18 variants is currently unknown.

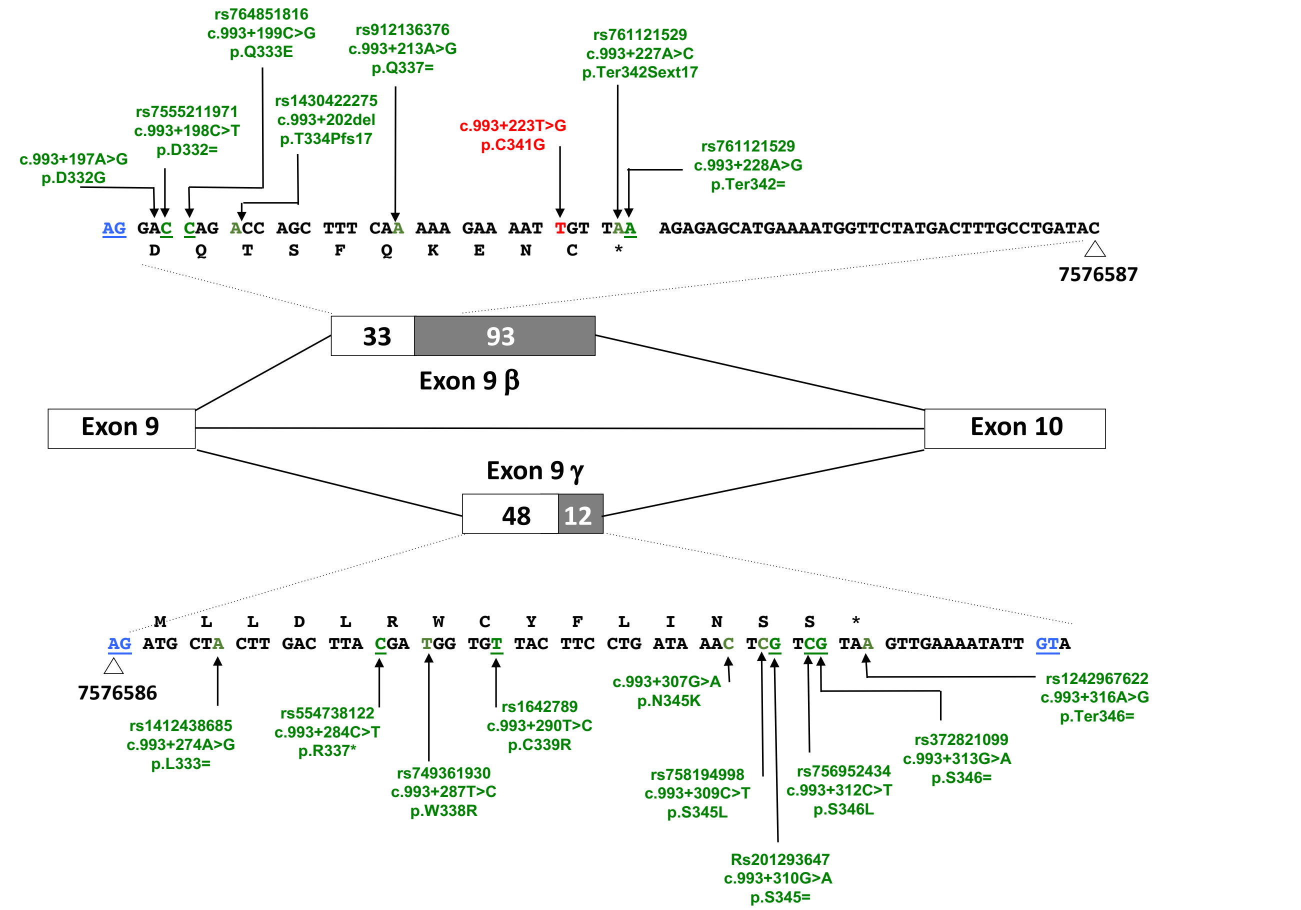

Fig. 5: TP53 variants detected in exon 9 beta and exon 9 gamma
Alternative splicing events in TP53 intron 9 lead to the expression of beta and gamma isoforms. Exons 9 beta and 9 gamma have different splice acceptor sites but share the same donor site (blue AG and GT sequences, respectively). Due to the marked overlap between the two exons, it is possible that variants localized in the translated region of exon 9 gamma impair exon 9 beta splicing. The main splicing event occurs between exons 9 and 10. The variant found specifically in the Asian population is shown in red.

Table 1: TP53 variant found in exon 9 beta and gamma in gnomAD v 3.0

rsID	cDNA_variant	TP53_beta	TP53_gamma	AC/AF ALL	AC_AFR	AC_LAT	AC_EAS	FIN	NFE	OTH	SAS
NA	c.993+197A>G	p.Asp332Gly	NR	1/(0.0000069)	0	0	0	0	1	0	0
NA	c.993+198C>T	p.Asp332Asp	NR	1/(0.0000069)	0	0	1	0	0	0	0
rs764851816	c.993+199C>G	p.Gln333Glu	NR	2/(0.0000139)	0	0	2	0	0	0	0
rs1430422275	c.993+203del	p.Thr334ProfsTer17	NR	1/(0.0000069)	0	0	0	0	1	0	0
rs912136376	c.993+213A>G	p.Gln337Gln	NR	1/(0.0000069)	0	0	0	0	1	0	0
NA	c.993+223T>G*	p.Cys341Gly	NR	14/(0.0000978)	0	0	0	0	0	0	14
rs764562217	c.993+227A>C	›.Ter342SerextTer1[illegible]	NR	5/(0.0000349)	0	0	0	0	5	0	0
rs761121529	c.993+228A>G	p.Ter342Ter	NR	10/(0.0000698)	0	0	0	0	10	0	0
rs1412438685	c.993+274A>G	NR	p.Leu333Leu	1/(0.0000069)	0	0	0	0	1	0	0
rs554738122	c.993+284C>T	NR	p.Arg337Ter	4/(0.0000280)	3	0	0	0	1	0	0
rs749361930	c.993+287T>C	NR	p.Trp338Arg	1/(0.0000069)	0	0	1	0	0	0	0
rs1642789	c.993+290T>C	NR	p.Cys339Arg	1/(0.0000069)	0	1	0	0	0	0	0
NA	c.993+307C>G	NR	p.Asn344Lys	2/(0.0000139)	0	0	0	0	2	0	0
rs758194998	c.993+309C>T	NR	p.Ser345Leu	1/(0.0000070)	0	0	0	0	1	0	0
NA	c.993+310G>A**	NR	p.Ser345Ser	10/(0.0000701)	4	1	0	0	5	0	0
rs756952434	c.993+312C>T	NR	p.Ser346Leu	5/(0.0000351)	0	2	0	0	3	0	0
rs372821099	c.993+313G>A***	NR	p.Ser346Ser	8/(0.0000561)	2	0	1	0	3	0	2
rs1242967622	c.993+316A>G	NR	p.Ter347Ter	1/(0.0000070)	1	0	0	0	0	0	0

This analysis was performed with gnomAD v3.0, which includes the full genome sequences of 71,702 unrelated individuals.

* In gnomAD V2.1, AC=67 with AC_SAS=66
** Also identified in four population datasets (Australia, England, Spain and EVS)
*** Also identified in four population datasets (Australia, England, Japan and EVS)

ALL: all populations; AFR: African/African American; EAS: East Asian; FIN: Finnish; NFE: Non-Finnish European; OTH: other unassigned populations; SAS: South Asian.

AC: allele count; AF: allele frequency

The unusual case of *TP53* variant c.523C>T;p.R175C

Variant p.R175C is very particular. It has been described as a somatic variant in multiple studies, can be found in all cancer genome databases and is considered pathogenic or likely pathogenic by most predictive algorithms. That status has been tied to the fact that variant p.R175H at this evolutionarily-conserved codon is a major hot spot found in human cancer and highly deleterious both *in vitro* and *in vivo* [1]. The three major mutation hot spots in the human *TP53* gene (codon 175, CGC; 248, CGG; and 273, CGC) contain a CpG dinucleotide known to be methylated *in vivo* (**Fig. 6**).

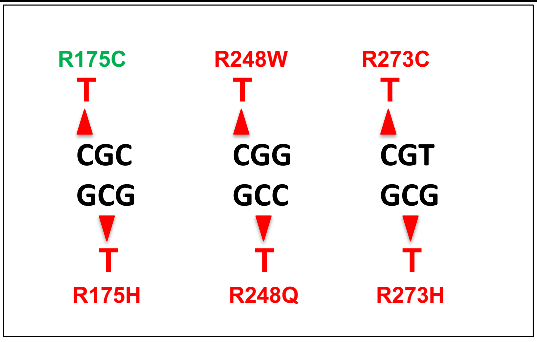

Fig. 6: *TP53* variants and CpG sites
a: Potential methylation-mediated deamination of 5-methylcytosine leading to C>T transition at a hot-spot codon of the TP53 gene. The five TP53 variants in red have been shown to be non-functional in multiple assays and are defined as TP53 hot spot variants.

Most mutations at these three positions are GC>AT transitions resulting from an endogenous mutational process initiated by spontaneous deamination of 5-methylcytosine. This pattern of mutation corresponds to signature 1 as defined by Alexandrov *et al*. and can be found in every type of cancer [12]. It also corresponds to the main mutational mechanism found for germline variants in many genetic diseases [13]. Examination of the frequency of transition at the two hot-spot codons 248 and 273 in the UMD_TP53 database showed a roughly equal frequency of C>T and G>A transitions, which was not surprising, given that the deamination of C residues in both strands of a symmetrical CpG dinucleotide will occur and be repaired at a similar rate. Furthermore, the four variants resulting from these events will lead to fully oncogenic *TP53* (**Fig. 1**). In contrast, codon 175 showed a marked disequilibrium in the distribution of mutations, with 3952 G>A transitions (p.R175H) and only 84 C>T transitions (p.R175C) (**Fig. 7**).

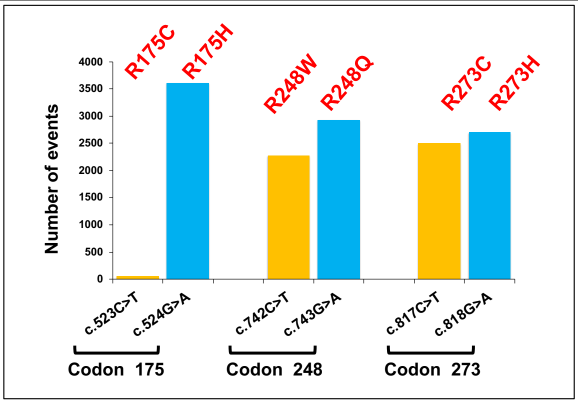

Fig. 7: Distribution of C>T and G>A transitions at codons 175, 248 and 273 of the TP53 gene showing the disequilibrium of mutation at codon 175.
Data were taken from the UMD_TP53 database. Results are similar with *TP53* variants from the TCGA database.

Furthermore, 37% of tumors expressing p.R175C also expressed a second *TP53* variant, compared with 8% of tumors expressing p.R175H, which indicates that p.R175C is not the driver mutation (**Fig. 8**).

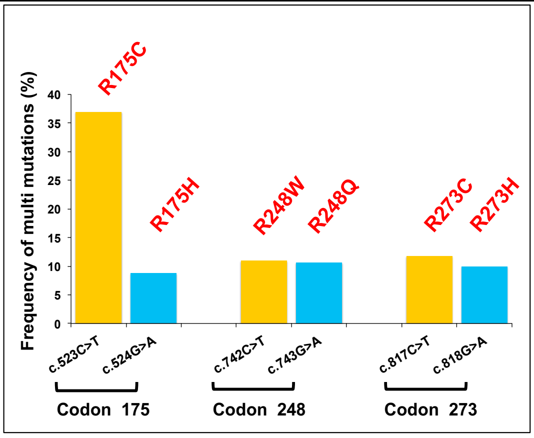

Fig. 8: Tumors expressing *TP53* variants p.R175C are more frequently associated with a second mutation compared with other TP53 variants.

Of note also, p.R175C has never been found as a germline variant in cancer-prone families, in contrast to the five other CpG variants at codons 175, 248 and 273. Moreover, transfection of the R175C mutant into p53-null human tumor Saos-2 cells essentially restores wild-type p53 cell-cycle arrest and proapoptotic functions, whereas these functions are lost upon introduction of large bulky side chains, as in the case of the R175W and R175Y mutations [1]. The p.R175C variant was however defective in transcription of the BAX promoter in a reporter gene assay [14]. It is also interesting to note that the R175A mutant is only moderately destabilized by 0.7 kcal/mol compared with 3.5 kcal/mol for the p.R175H mutant, suggesting that the stability loss of the p.R175H mutant is largely due to the introduction of the imidazole ring inducing steric clashes in the zinc-binding region, rather than the loss of interactions mediated by the guanidinium group [15, 16].

Weighing this range of information, we conclude that p.R175C is highly likely to be a passenger mutation co-selected during neoplastic transformation, and/or a very infrequent *de novo TP53* benign mutation.

Reference

1. Ory K, Legros Y, Auguin C, Soussi T Analysis of the most representative tumour-derived p53 mutants reveals that changes in protein conformation are not correlated with loss of transactivation or inhibition of cell proliferation. EMBO J.1994; 13:3496-3504
2. Bessard AC, Garay E, Lacronique V, Legros Y, Demarquay C, Houque A *et al.* Regulation of the specific DNA binding activity of Xenopus laevis p53: evidence for conserved regulation through the carboxy-terminus of the protein. Oncogene.1998; 16:883-890
3. Flaman JM, Frebourg T, Moreau V, Charbonnier F, Martin C, Chappuis P *et al.* A simple p53 functional assay for screening cell lines, blood, and tumors. Proc Natl Acad Sci U S A.1995; 92:3963-3967
4. Grochova D, Vankova J, Damborsky J, Ravcukova B, Smarda J, Vojtesek B *et al.* Analysis of transactivation capability and conformation of p53 temperature-dependent mutants and their reactivation by amifostine in yeast. Oncogene.2008; 27:1243-1252
5. Campomenosi P, Monti P, Aprile A, Abbondandolo A, Frebourg T, Gold B *et al.* p53 mutants can often transactivate promoters containing a p21 but not Bax or PIG3 responsive elements. Oncogene.2001; 20:3573-3579
6. Bienert S, Waterhouse A, de Beer TA, Tauriello G, Studer G, Bordoli L *et al.* The SWISS-MODEL Repository-new features and functionality. Nucleic Acids Res.2017; 45:D313-D319
7. Natan E, Baloglu C, Pagel K, Freund SM, Morgner N, Robinson CV *et al.* Interaction of the p53 DNA-Binding Domain with Its N-Terminal Extension Modulates the Stability of the p53 Tetramer. J Mol Biol.2011; 409:358-368
8. Pucci F, Bourgeas R, Rooman M Predicting protein thermal stability changes upon point mutations using statistical potentials: Introducing HoTMuSiC. Sci Rep.2016; 6:23257
9. Stacey SN, Sulem P, Jonasdottir A, Masson G, Gudmundsson J, Gudbjartsson DF *et al.* A germline variant in the TP53 polyadenylation signal confers cancer susceptibility. Nat Genet.2011; 43:1098-1103
10. Wang Y, Wu XS, He J, Ma T, Lei W, Shen ZY A novel TP53 variant (rs78378222 A > C) in the polyadenylation signal is associated with increased cancer susceptibility: evidence from a meta-analysis. Oncotarget.2016; 7:32854-32865
11. Ahuja HG, Testa MP, Cline MJ Variation in the protein coding region of the human p53 gene. Oncogene.1990; 5:1409-1410
12. Alexandrov LB, Stratton MR Mutational signatures: the patterns of somatic mutations hidden in cancer genomes. Curr Opin Genet Dev.2014; 24:52-60
13. Krawczak M, Smith-Sorensen B, Schmidtke J, Kakkar VV, Cooper DN, Hovig E Somatic spectrum of cancer-associated single basepair substitutions in the TP53 gene is determined mainly by endogenous mechanisms of mutation and by selection. Hum Mutat.1995; 5:48-57
14. Ryan KM, Vousden KH Characterization of structural p53 mutants which show selective defects in apoptosis but not cell cycle arrest. Mol Cell Biol.1998; 18:3692-3698
15. Bullock AN, Henckel J, Fersht AR Quantitative analysis of residual folding and DNA binding in mutant p53 core domain: definition of mutant states for rescue in cancer therapy. Oncogene.2000; 19:1245-1256
16. Joerger AC, Fersht AR Structure-function-rescue: the diverse nature of common p53 cancer mutants. Oncogene.2007; 26:2226-2242
