# Supplementary material for: Identification and functional characterization of new missense SNPs in the coding region of the TP53 gene
Source: Cell Death Differ. 2020 Nov 30;28(5):1477–92. doi: 10.1038/s41418-020-00672-0 (PMC8166836; doi:10.1038/s41418-020-00672-0)
Supplement: Supplementary file 2 — Supplementary Figures and Tables [file 41418_2020_672_MOESM2_ESM.pdf]

**Supplementary Figures and tables**

**Full Title: Identification and functional characterization of new missense SNPs in the coding region of the *TP53* gene**

Flora Doffe, Vincent Carbonnier, Manon Tissier, Bernard Leroy, Isabelle Martins, Johanna S.M. Mattsson, Patrick Micke, Sarka Pavlova, Sarka Pospisilova, Jana Smardova, Andreas C. Joerger, Klas G. Wiman, Guido Kroemer and Thierry Soussi

Corresponding author: Thierry Soussi
thierry.soussi@sorbonne-universite.fr

**This PDF file includes:**

**Figures S1 to S11**
**Tables S1 to S6**

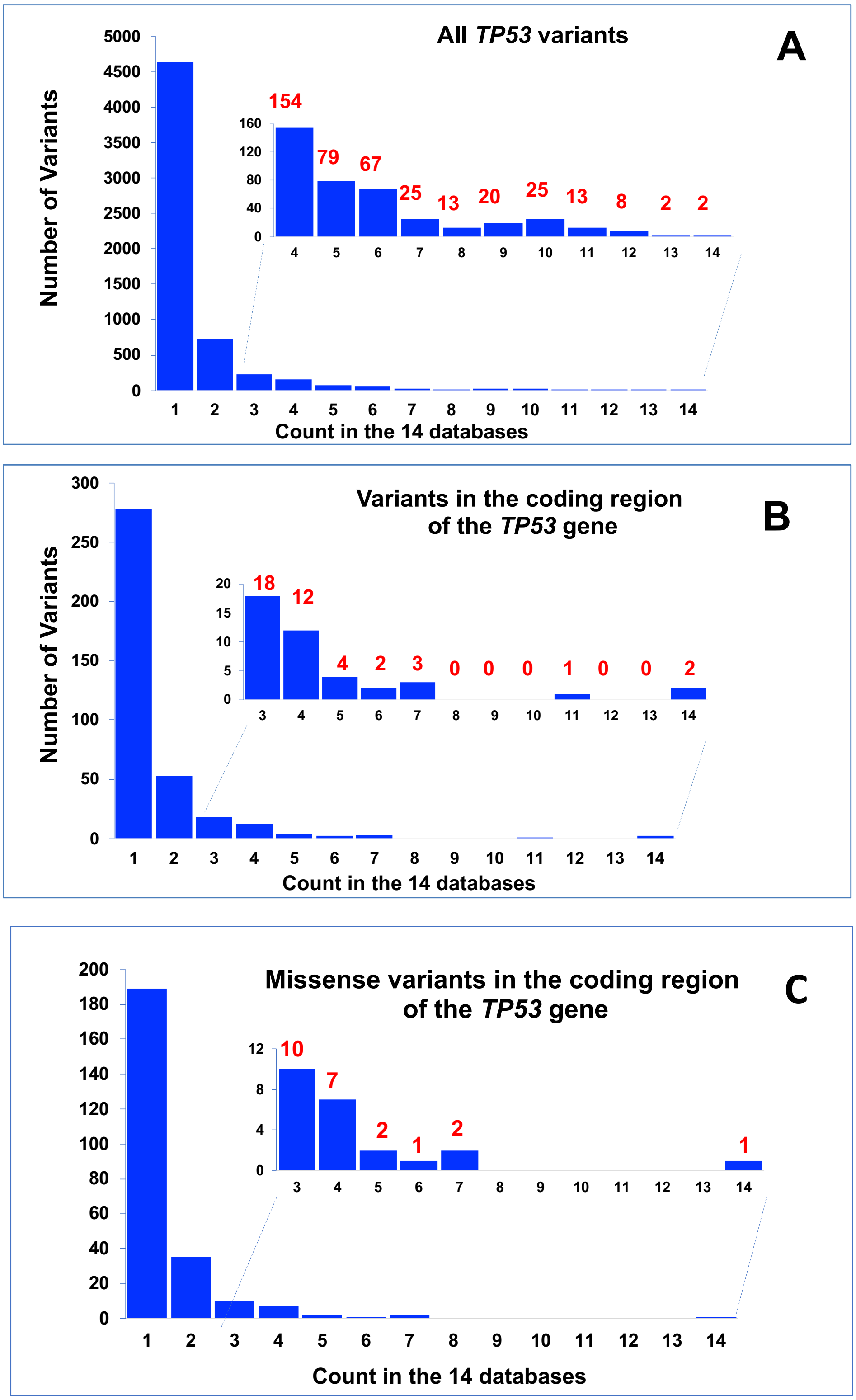


**Fig. S1 (part 1): Occurrence of *TP53* variants in the 14 datasets**

(a) *TP53* variants in the whole *TP53* gene (NG_017013.2). (b) *TP53* variants in the coding region of the *TP53* gene (NM_000546.5). (c) Missense *TP53* variants in the coding region of the *TP53* gene (NM_000546.5). Only two variants were found in all 14 datasets, i.e. rs1042522 (p.P72R) and rs1800370 (p.P36=).

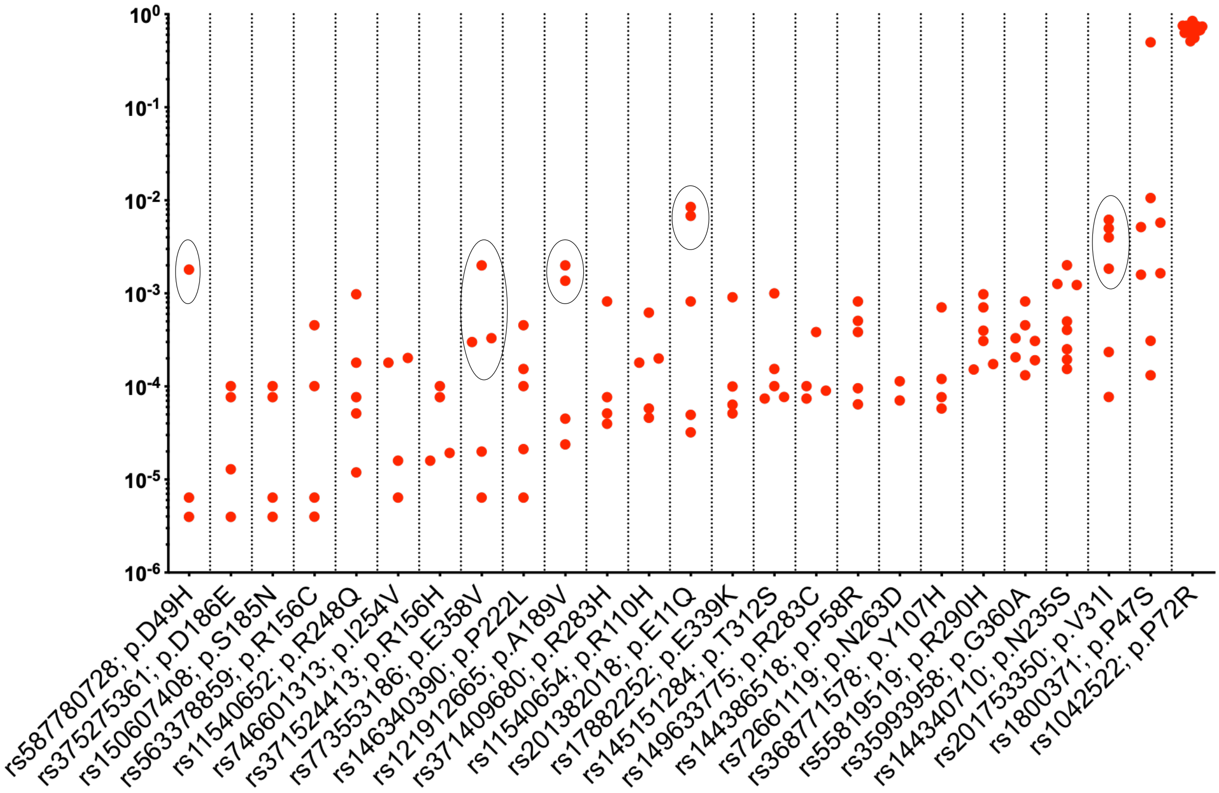


**Fig. S1 (part 2): Occurrence of *TP53* variants in the 14 datasets**

(d) Allele frequency of the most common TP53 variants found in the present study. For each variant found in four or more population datasets, the allele frequency for each dataset is shown. For five variants, the Asian populations have been circled to show the preferential origin of these variants.

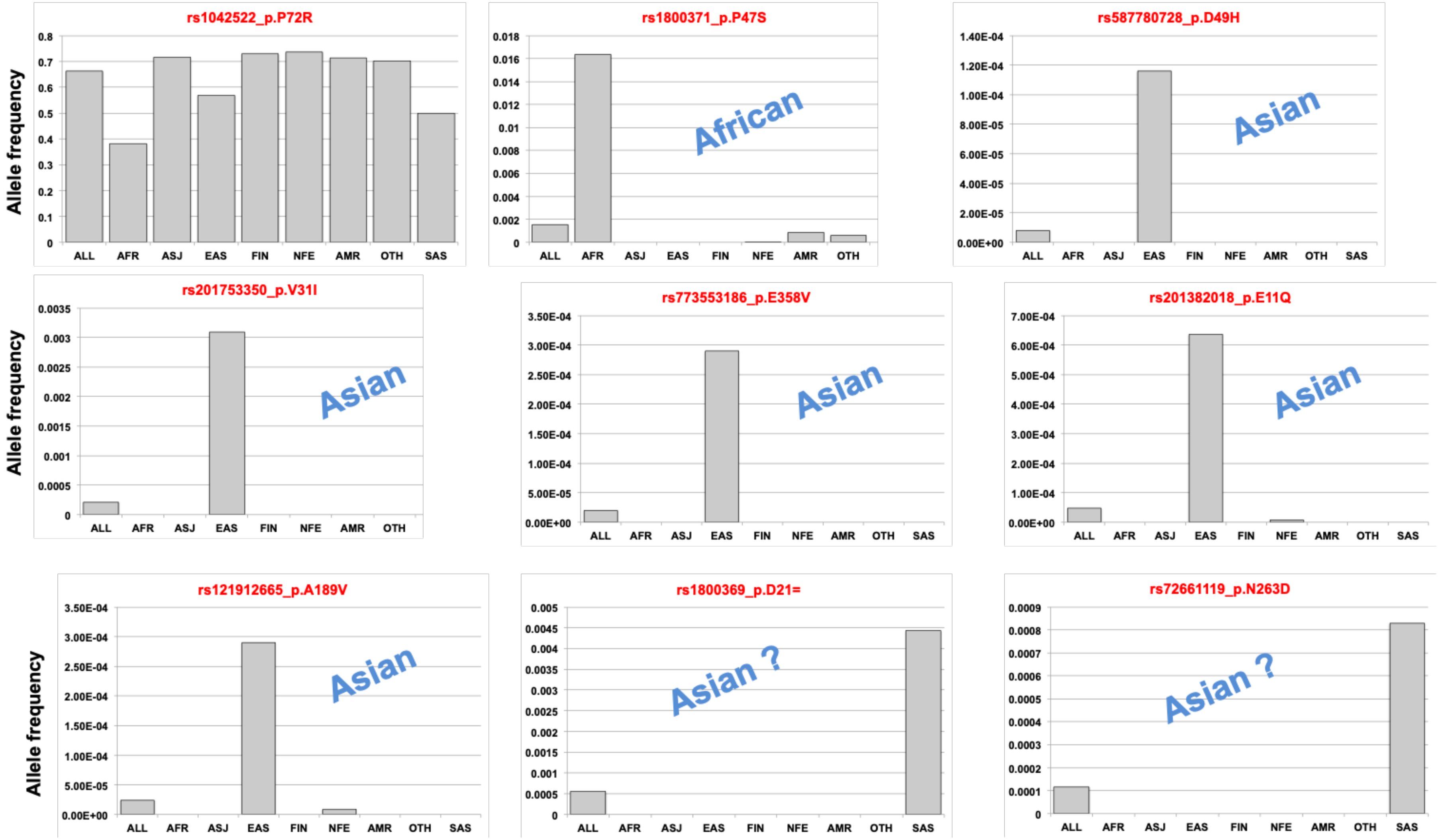


**Fig. S2 (part 1): Distribution of *TP53* SNPs in various subpopulations included in gnomAD**
See legend of Fig. S2 (part 3) for further details.

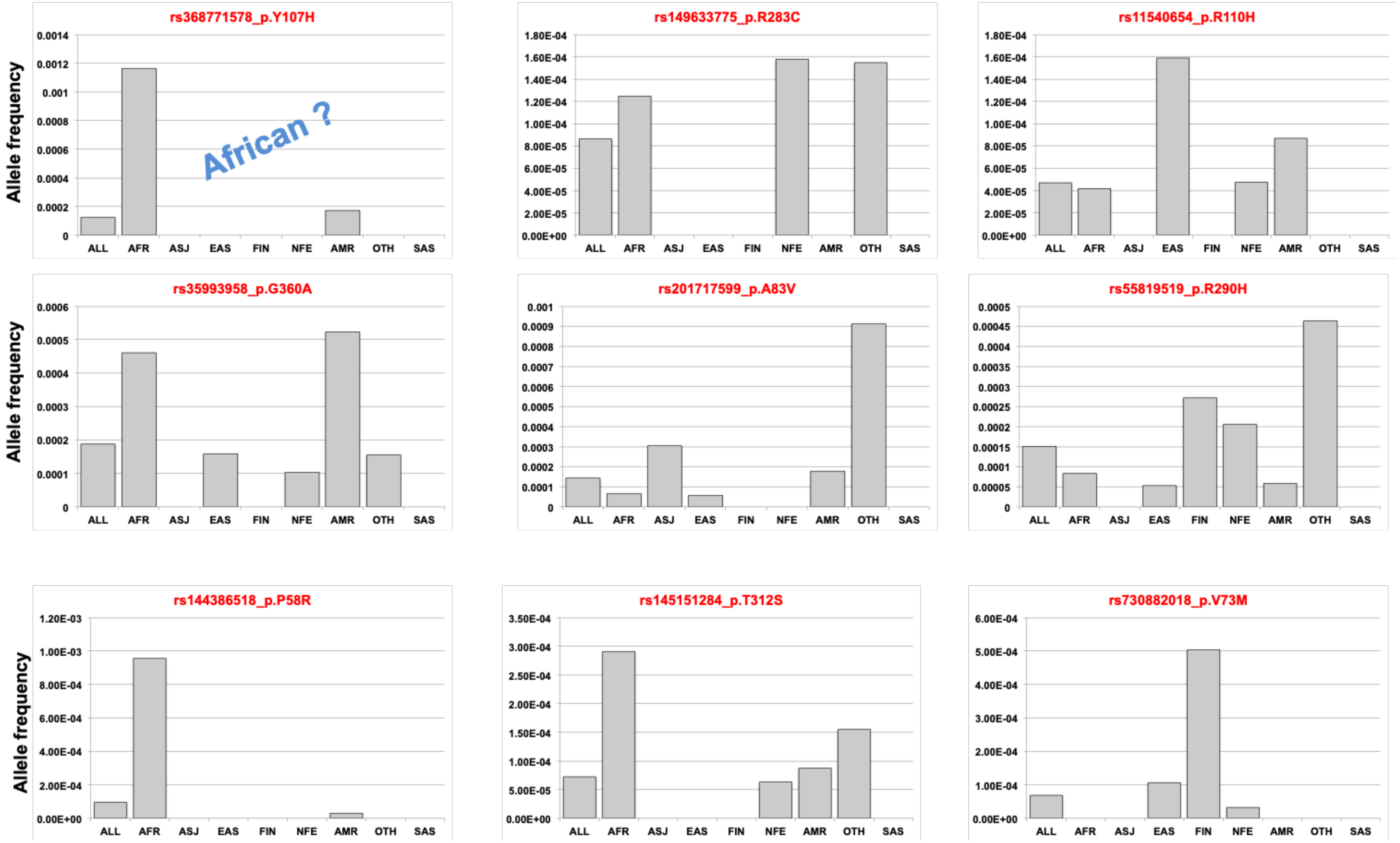


**Fig. S2 (part 2)**: **Distribution of *TP53* SNPs in various subpopulations included in gnomAD**
See legend of Fig. S2 (part 3) for further details.

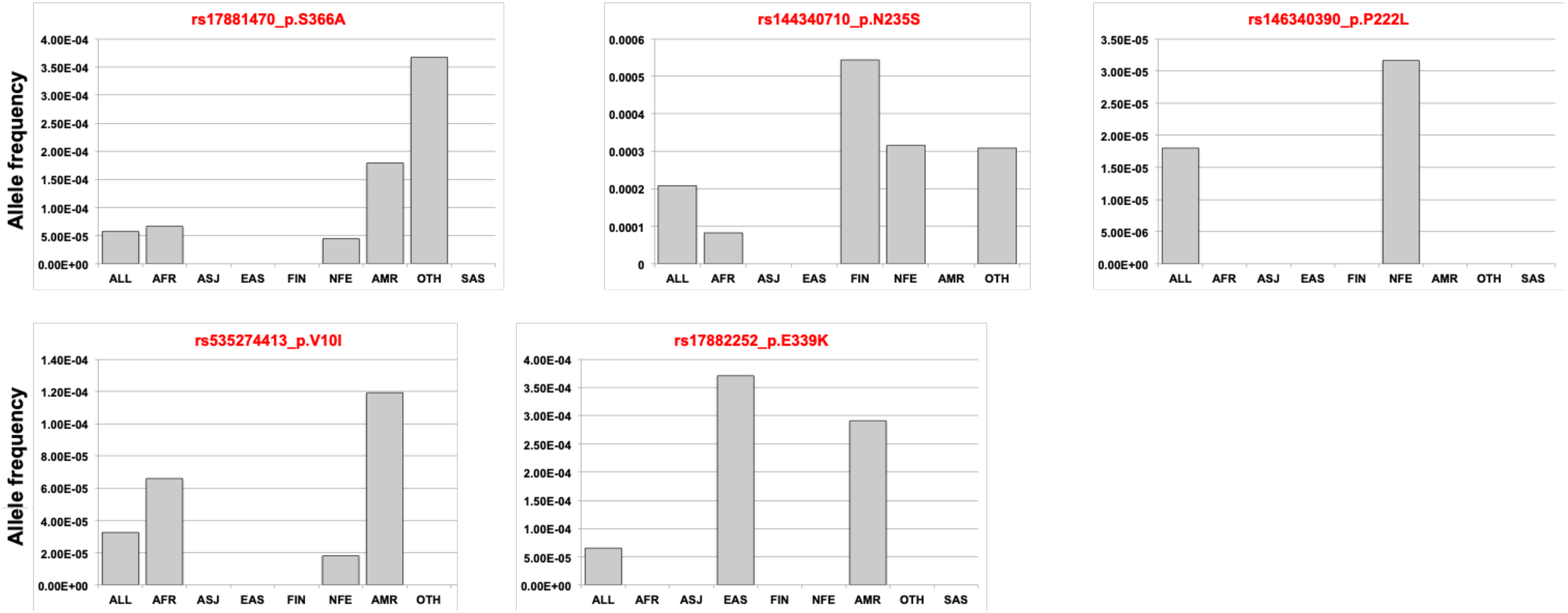


**Fig. S2 (part 3)**: **Distribution of *TP53* SNPs in various subpopulations included in gnomAD**

rs1042522 (NM_000546.5:c.215C>G; p.P72R) was found in all populations whereas rs1800371 (p.P47S) was specific to the African population. Five missense variants were detected only in Asian populations.

ALL: all populations; AFR: African/African American; AMR: admixed American; ASJ: Ashkenazi Jewish; EAS: East Asian; FIN: Finnish; NFE: Non-Finnish European; OTH: other unassigned populations; SAS: South Asian.

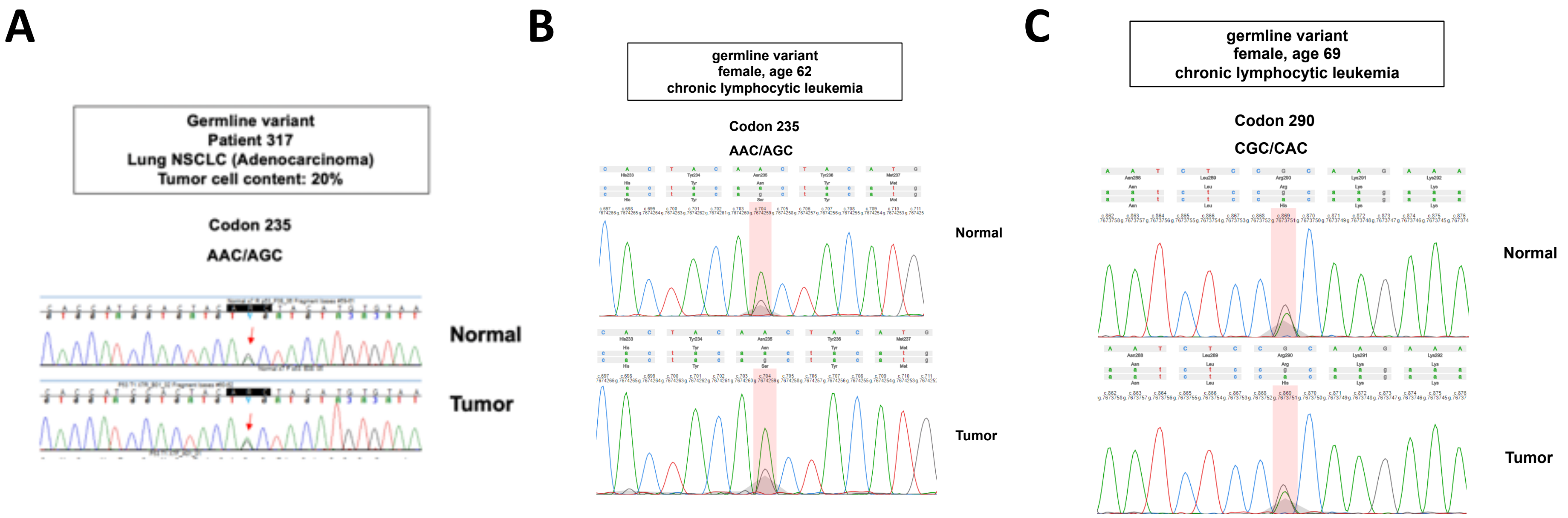


**Fig. S3 Germline origin of *TP53* variants (part 1)**

See legend of Fig. S3 (part 2) for further details.

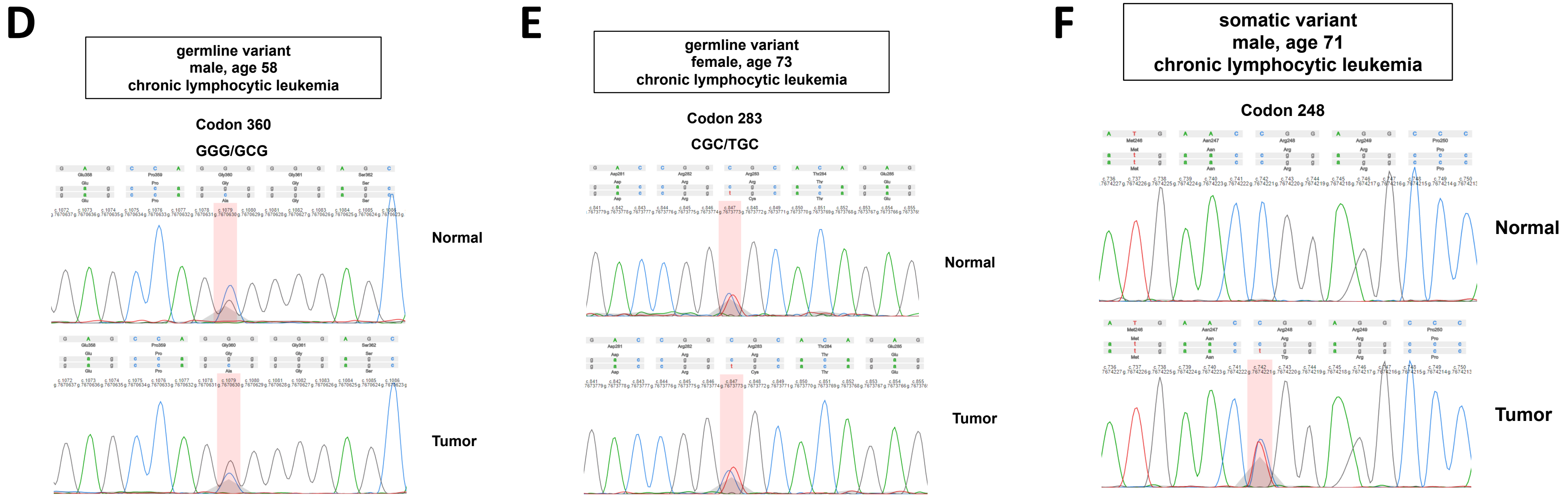


**Fig. S3 Germline origin of *TP53* variants (part 2)**

(a) Germline origin of *TP53* variant p.N235S in a lung adenocarcinoma patient. Patient 317 was part of a retrospective cohort of surgically-resected non-small cell lung cancer (NSCLC) patients who underwent surgery in Uppsala, Sweden, between 1995 and 2005 [1]. DNA from the normal tissue of patient 317 was extracted and sequenced. The results showed that this mutation, previously described as somatic, was indeed germline. The analysis was done in accordance with the Swedish Biobank Legislation and Ethical Review Act (#2006/325). Both tumoral (25% tumor cell content) and normal tissue from patient 317 were also used in the analysis.

(b-f) Germline origin of *TP53* variants p.R290H, p.R283C, p.G360A and p.N235S in patients with chronic lymphocytic leukemia (CLL). *TP53* analysis was performed as a part of routine clinical diagnostics in patients with CLL. Peripheral blood and buccal swab samples were taken after written informed consent in accordance with the Declaration of Helsinki under protocols approved by the Ethical Committee of the University Hospital Brno. Peripheral B lymphocytes were negatively separated using STEMCELL RosetteSep kits. The tumor cell fractions (CD5+CD19+) in separated samples exceeded 95% as verified by flow cytometry. The coding sequence of the *TP53* gene was analyzed by Sanger sequencing and NGS (data not shown). The hot-spot variant of somatic origin p.R248W is shown for comparison (panel f). Sequences were analyzed using the web-based Glass tool and GRCh38 as reference sequences [2].

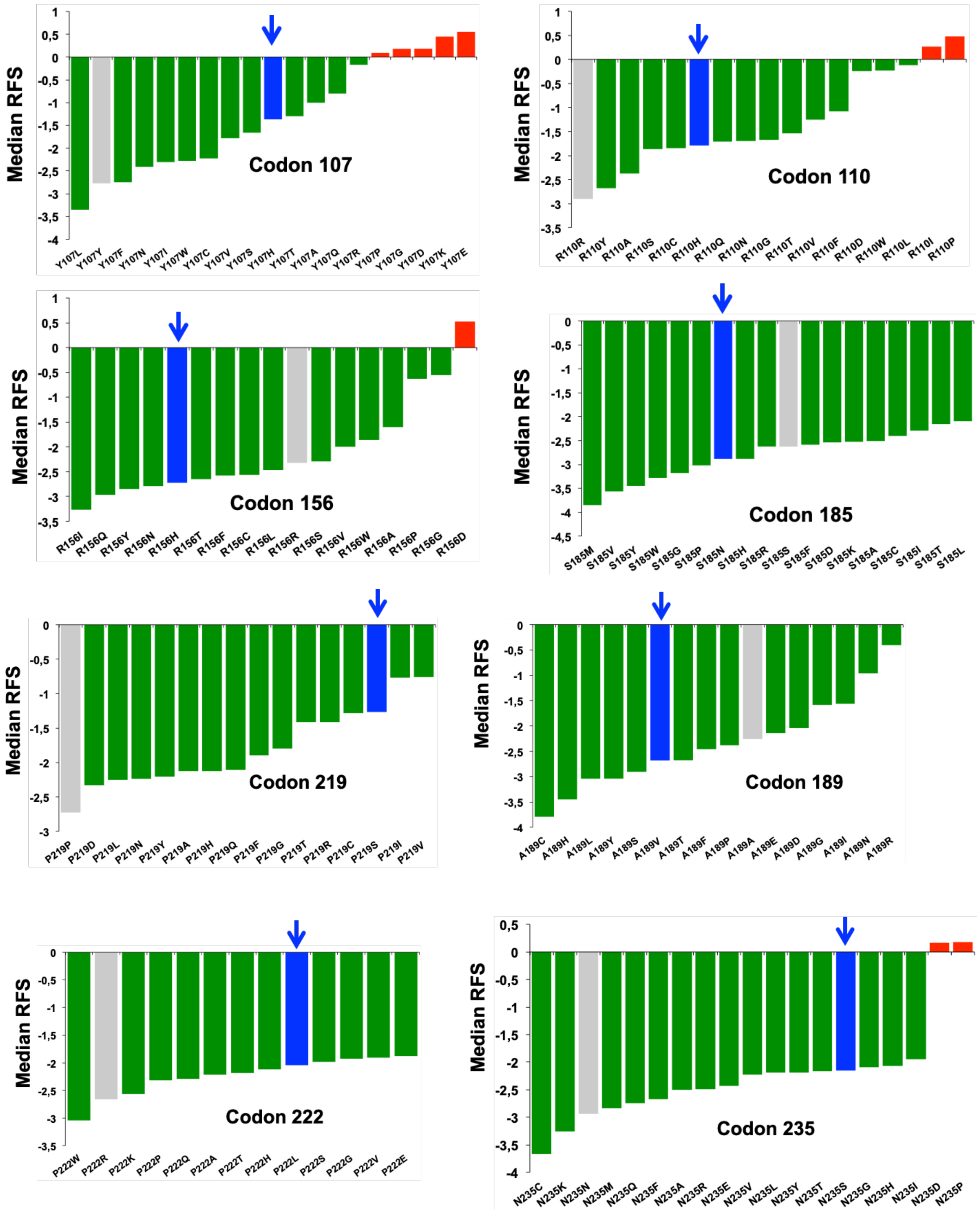


**Fig. S4: Recurrent *TP53* variants found in the human population are not impaired for growth suppression (part 1).**

See legend of Fig. S4 (part 2) for further details.

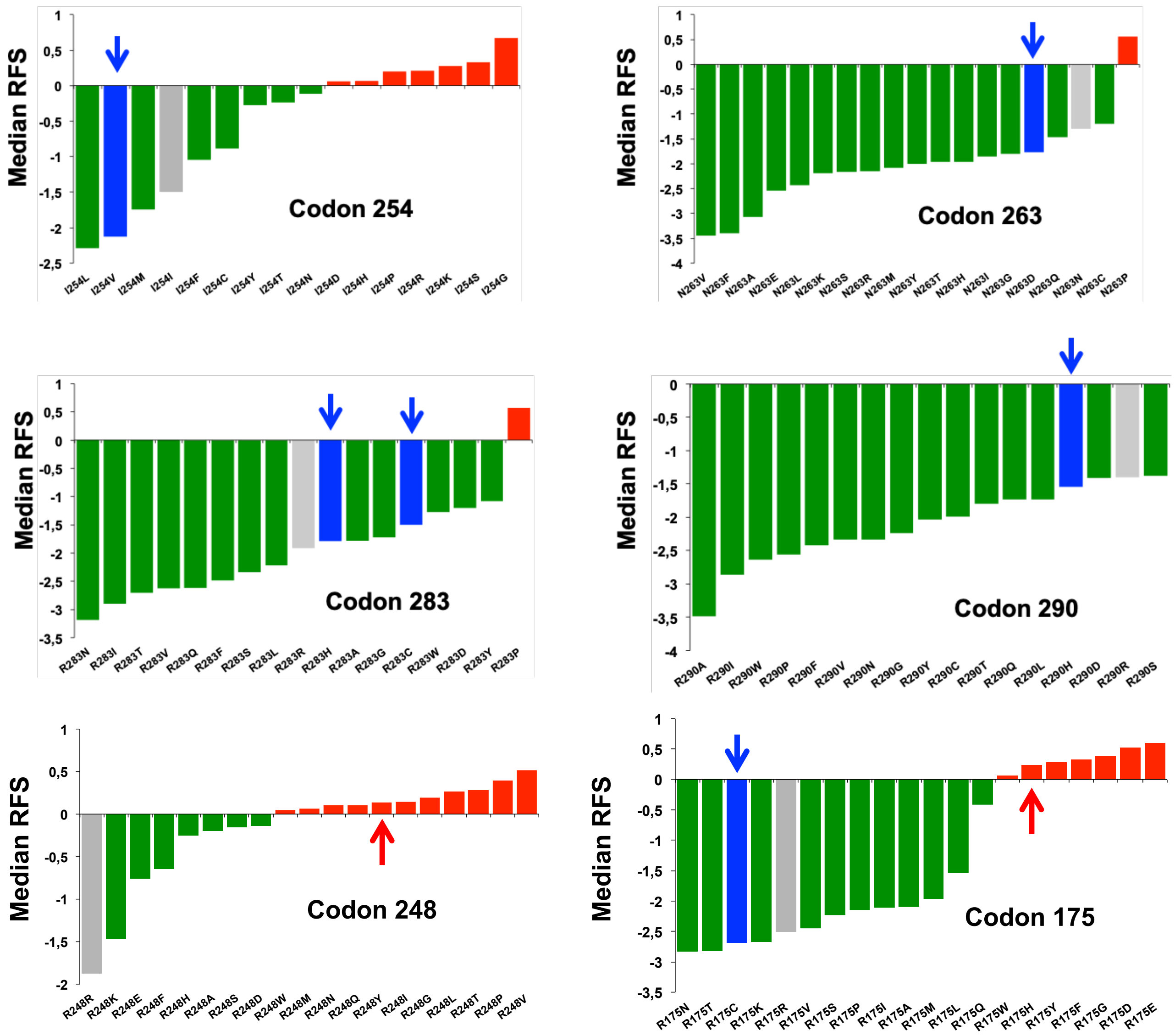


**Fig. S4: Recurrent *TP53* variants found in the human population are not impaired for growth suppression (part 2).**

The relative fitness score (RFS) for each *TP53* variant was extracted from the data of Kotler *et al*. and tabulated for different positions in the p53 protein [3]. Grey bars: synonymous variants; blue bars with arrow: *TP53* variants analyzed in the present study. Values below or over 0 represent *TP53* variants retaining or losing antiproliferative activity. All retrieved *TP53* variants retained wild-type behavior, with none of them displaying RFS values superior to -1.5. Hot-spot *TP53* variants found in human cancer such as p.R175H or p.R248Q (red arrows) had values superior to 0.

**Supplementary figure 5**

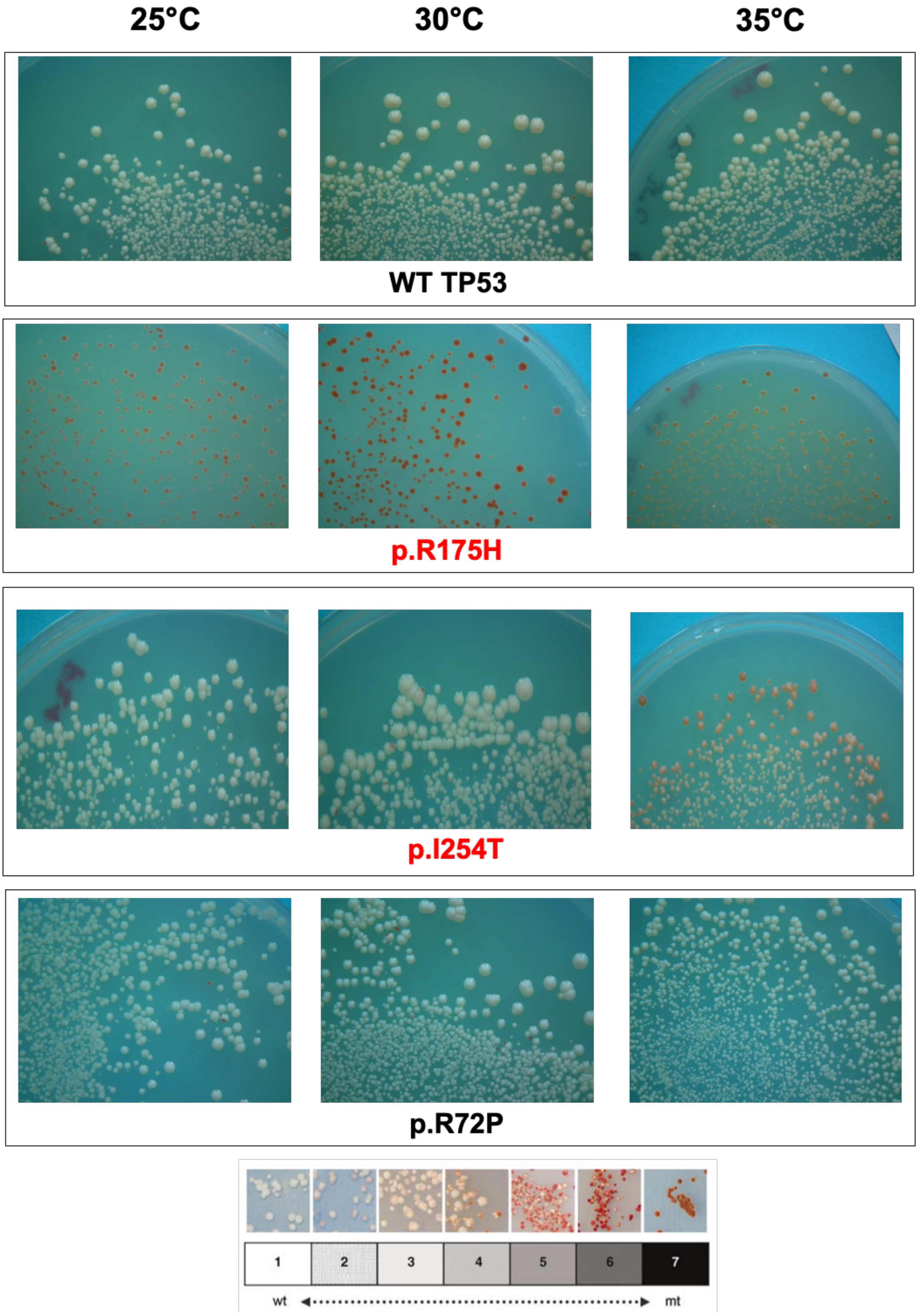


**Fig. S5 (part 1): Recurrent *TP53* variants found in the human population are transcriptionally competent in yeast**
See legend of Fig. S5 (part 4) for further details.

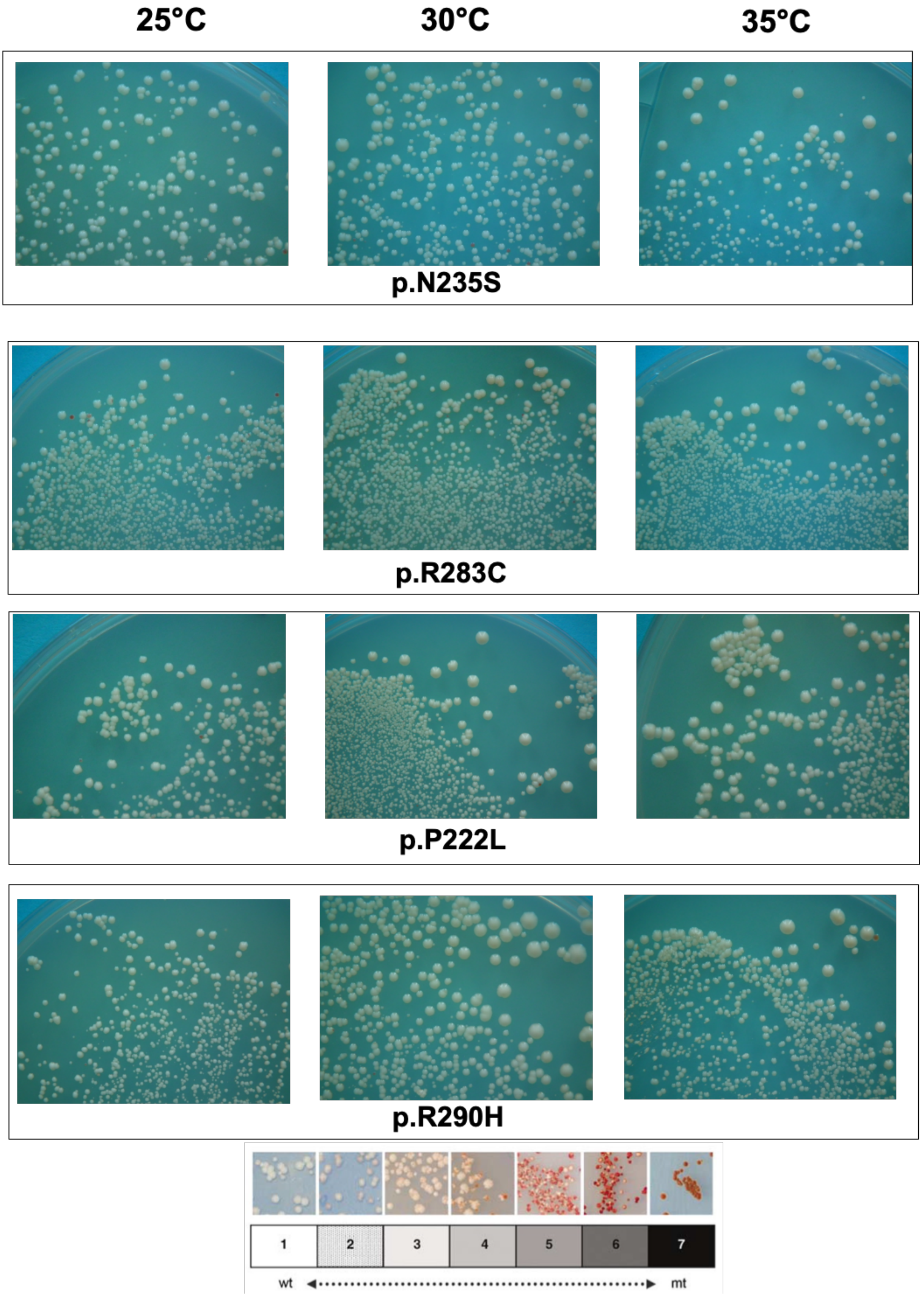


**Fig. S5 (part 2): Recurrent *TP53* variants found in the human population are transcriptionally competent in yeast using WAF1 promoter**
See legend of Fig. S5 (part 4) for further details.

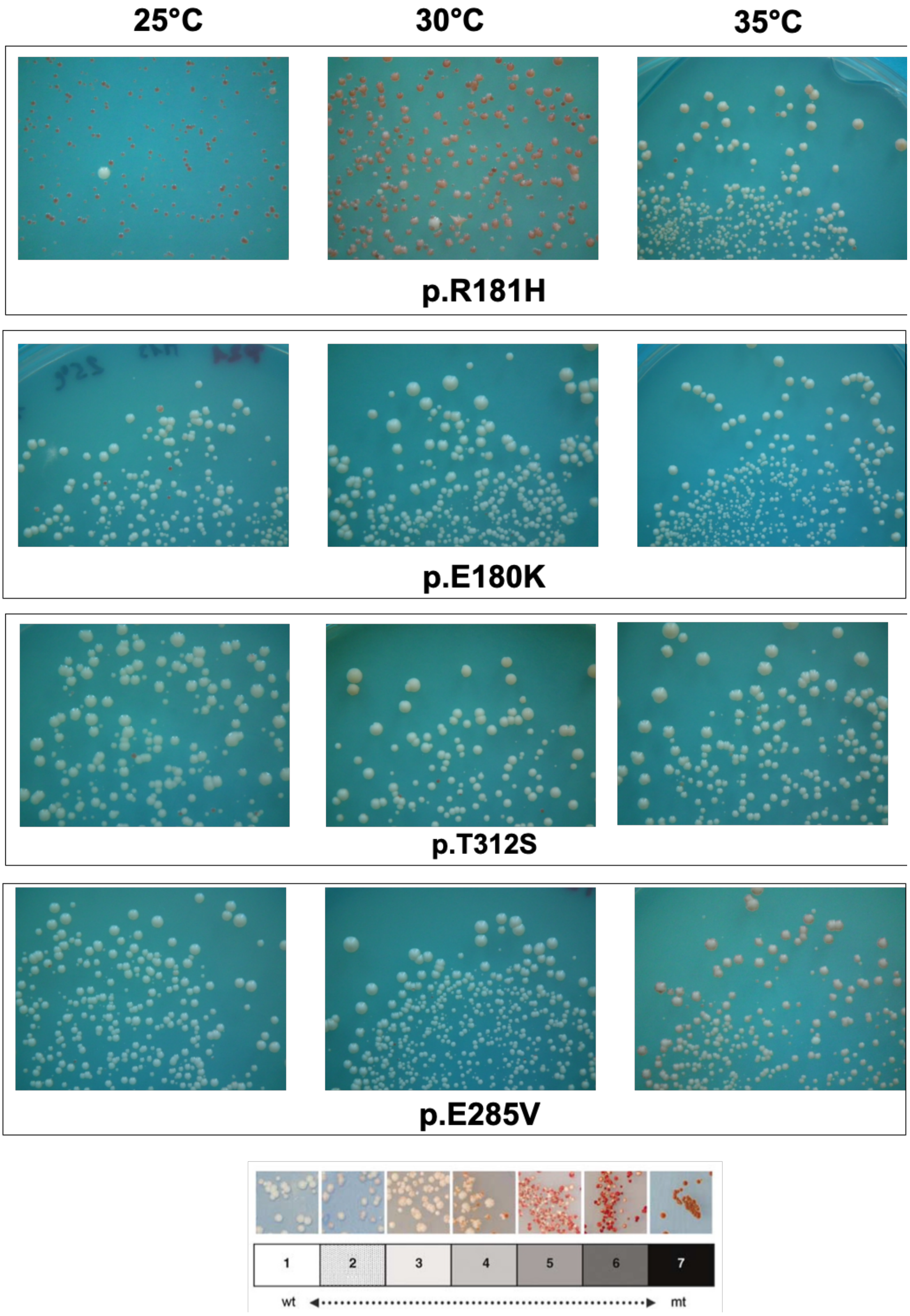


**Fig. S5 (part 3): Recurrent *TP53* variants found in the human population are transcriptionally competent in yeast**
See legend of Fig. S5 (part 4) for further details.

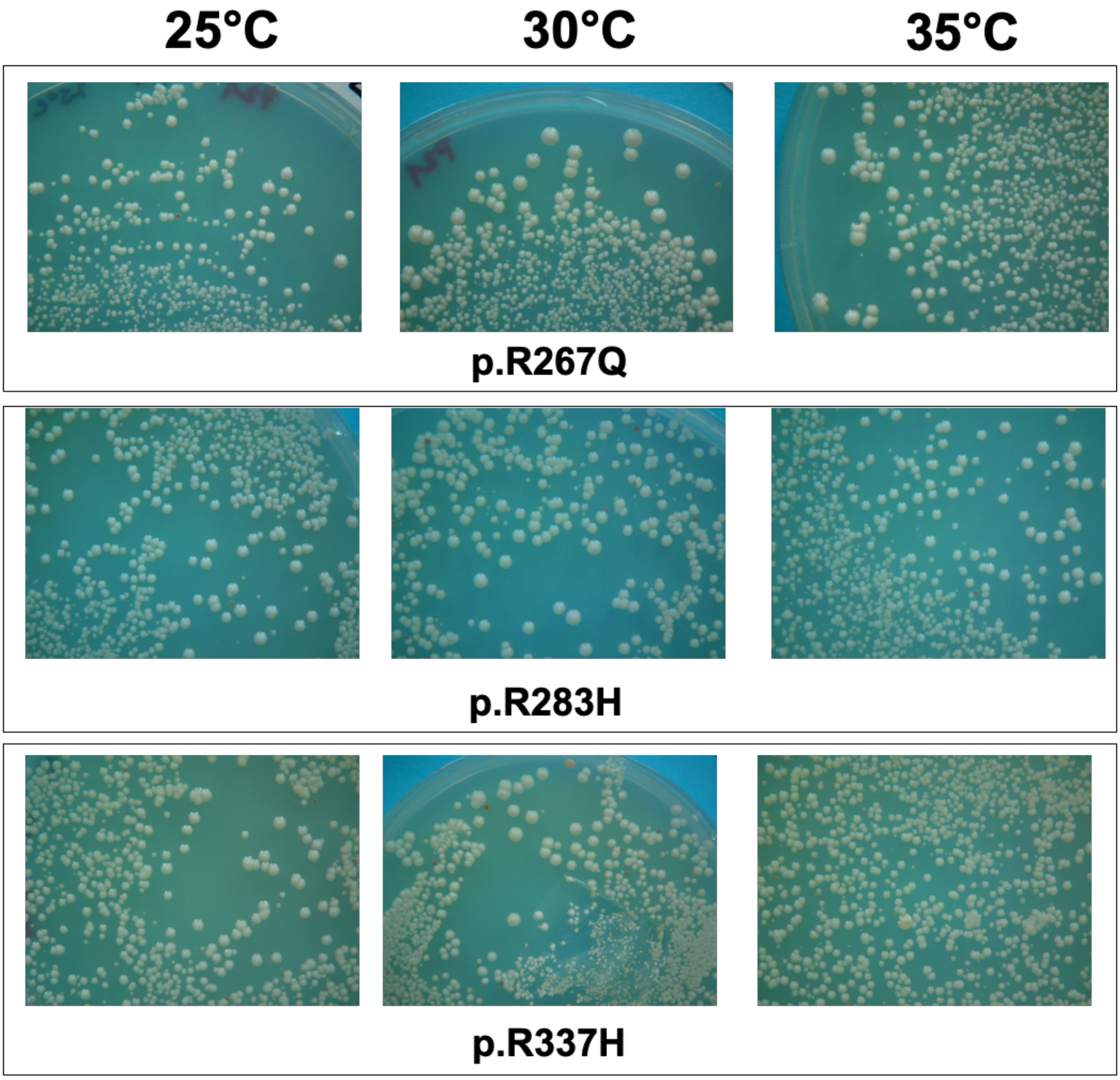


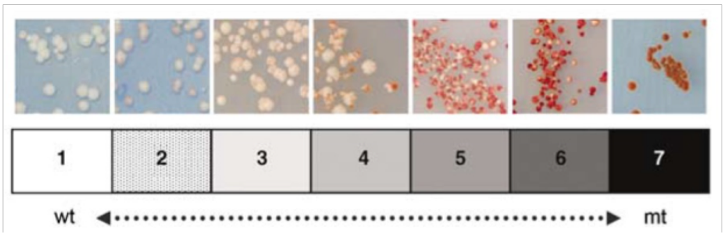


**Fig. S5 (part 4): Recurrent *TP53* variants found in the human population are transcriptionally competent in yeast**

White and red yeast colonies indicate respectively transcriptionally active and inactive *TP53* variants. Because FASAY can identify temperature-dependent variants, yeasts were plated at three different temperatures (25, 30 and 35 °C). Two cancer-derived variants were used as positive controls. These were variant p.R175H, which is fully inactive at all temperatures, and variant p.I254T, which is temperature sensitive and inactive only at 35 °C [4].

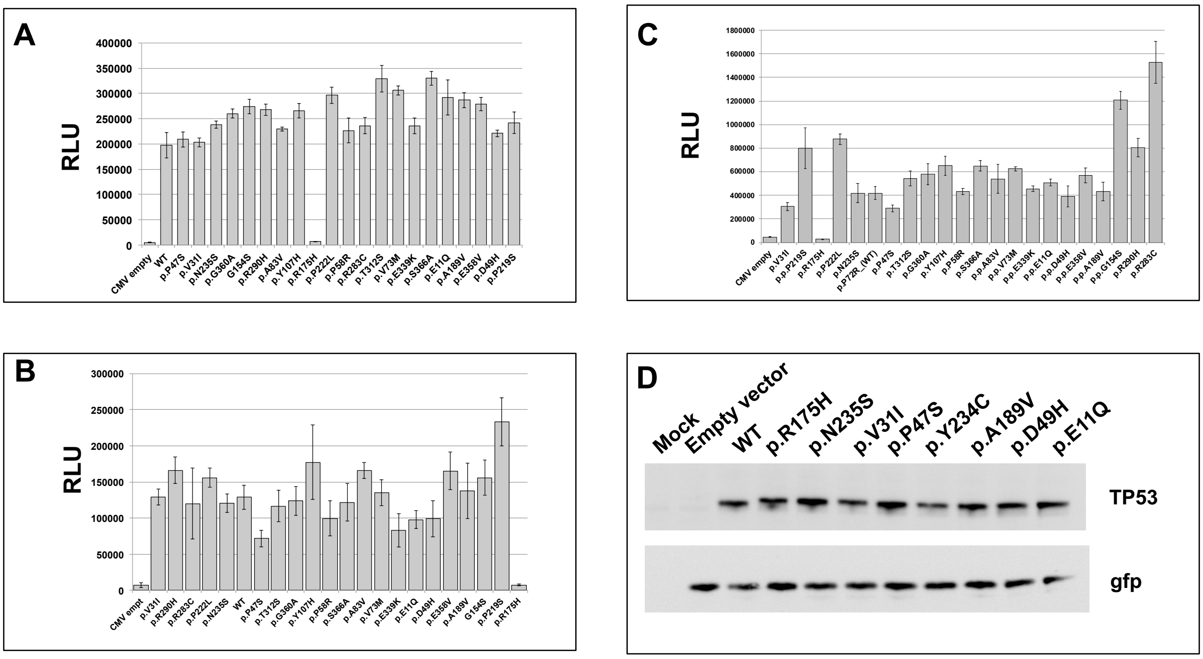


**Fig. S6: Recurrent *TP53* variants found in the human population are transcriptionally competent in mammalian cells**

MDM2 (a and b) or WAF1 (c) promoters upstream of the luciferase reporter were transiently transfected in H1299 cells with a range of *TP53* variants. Luciferase activity in the cell lysates was determined at 24 hours after transfection. (d) *TP53* and gfp expressing plasmids were cotransfected in H1299 cells. Western blot analysis with *TP53* and gfp antibodies showed equal expression for each *TP53* variant. Control experiments with wt TP53 in figure 6a, 6b and 6d were performed with TP53 Pro72 and with Arg72 in figure 6c. In our experimental conditions, we have never observed significant difference between the two alleles (T Soussi, unpublished observations). All variants were constructed with a Pro72 background).

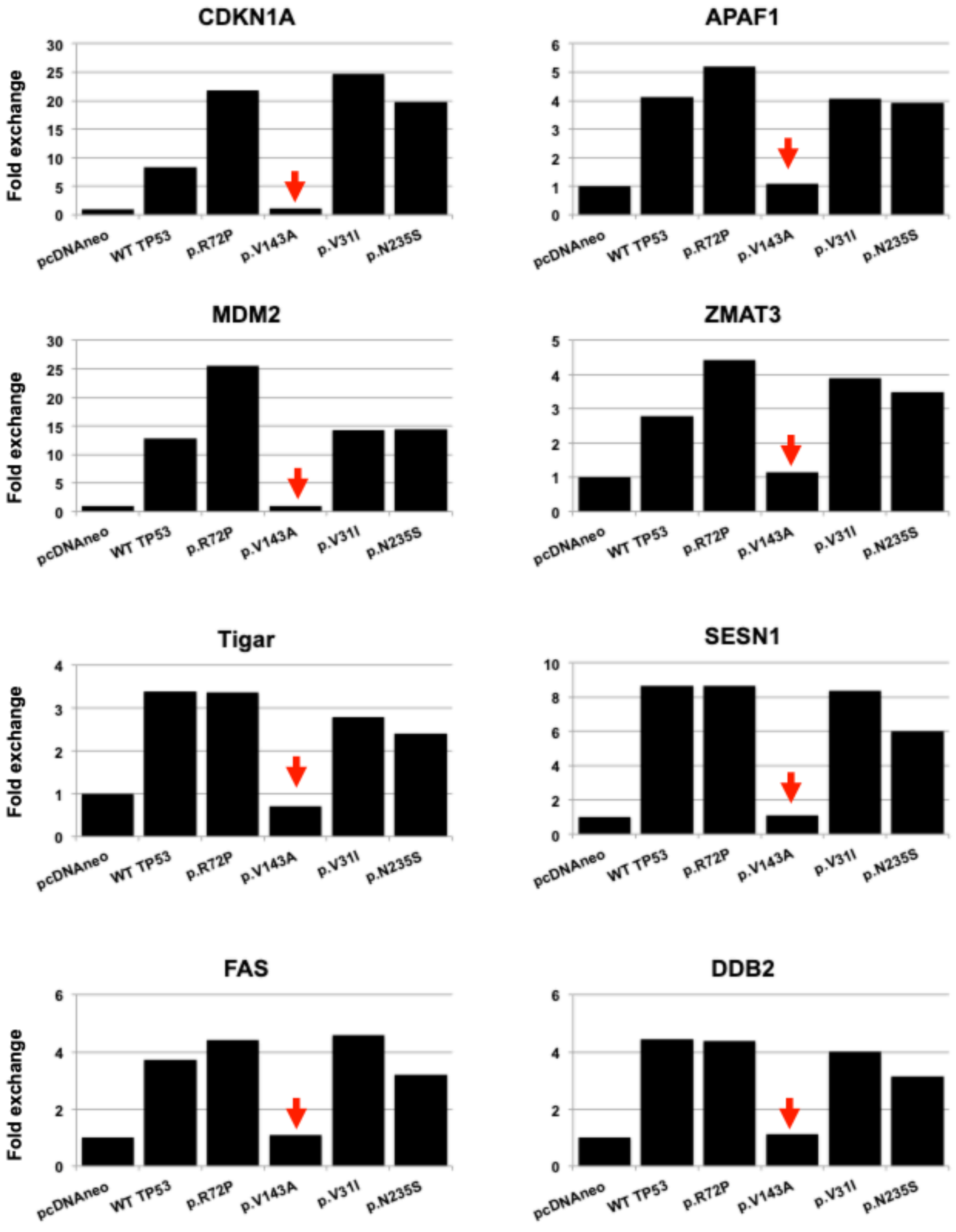


**Fig. S7 (part 1): Recurrent *TP53* variants found in the human population activate a wide panel of *TP53*-regulated genes**

qRT-PCR analysis of *TP53* target genes in H1299 cells 24 hours after transfection with a range of *TP53* variants. Red arrows: control with a *TP53*-defective variant.

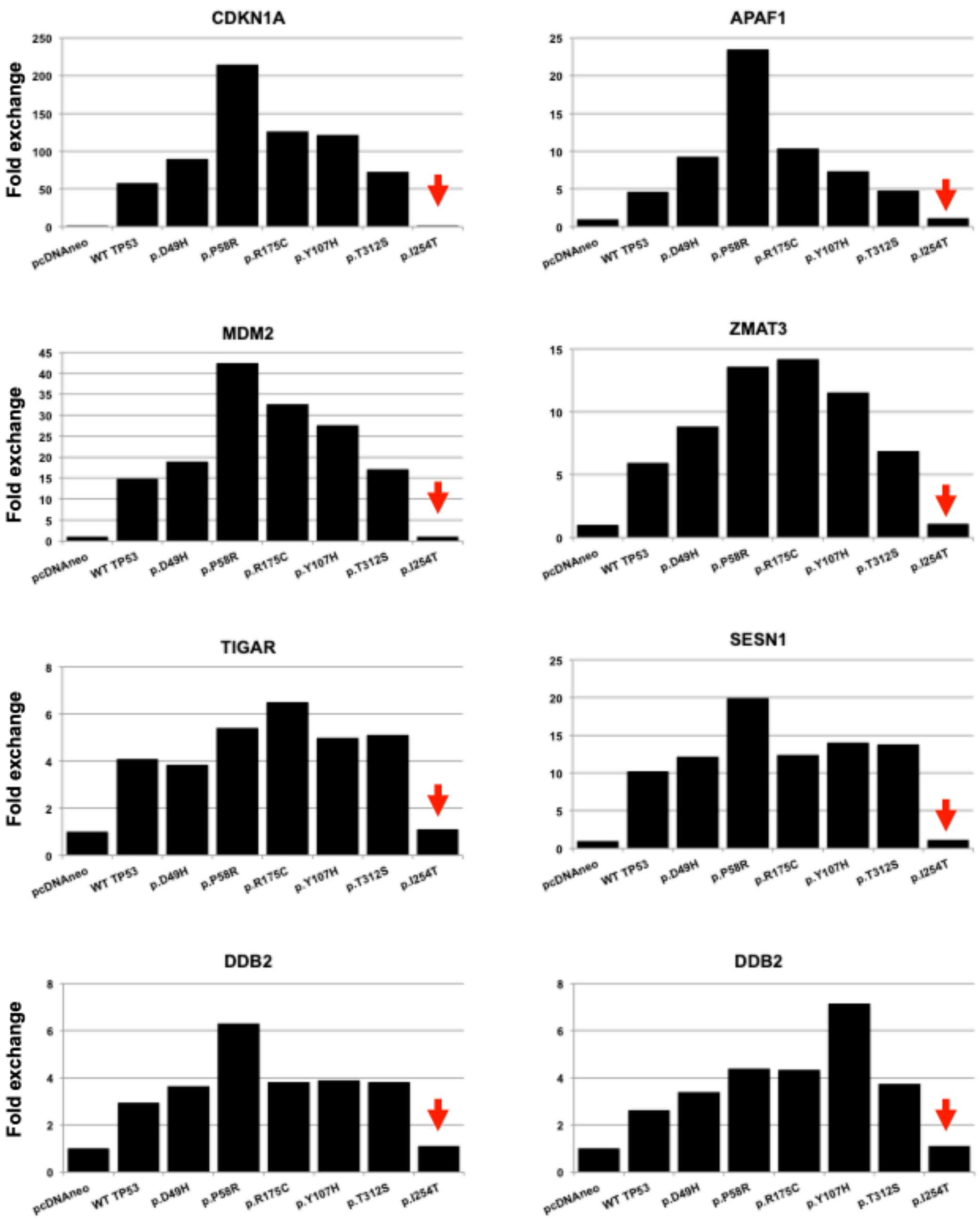


**Fig. S7 (part 2): Recurrent *TP53* variants found in the human population activate a wide panel of *TP53*-regulated genes**

See legend of Fig. S7 (part 1) for further details.

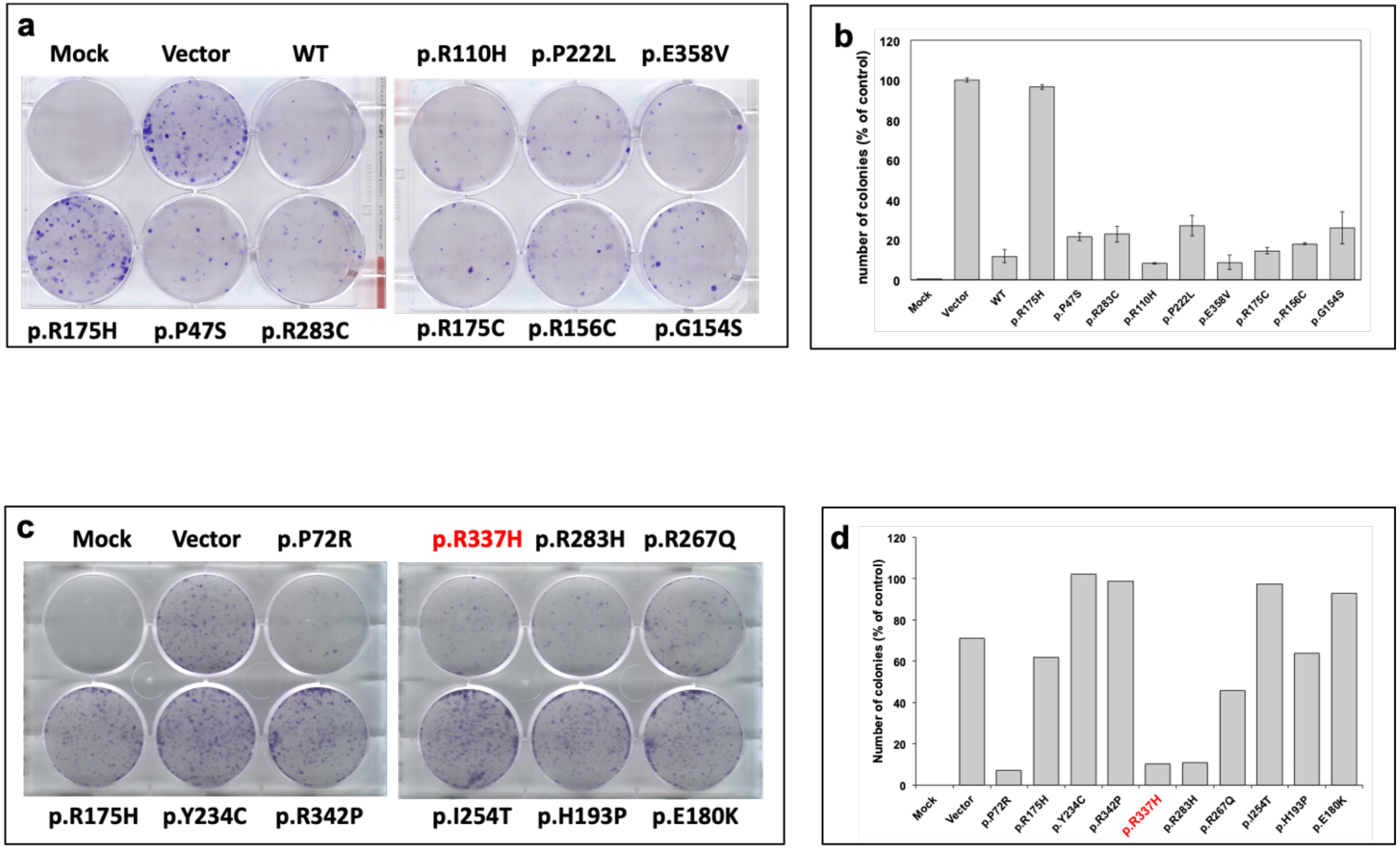


**Fig. S8: Recurrent *TP53* variants found in the human population inhibit cellular growth**

(a and c) H1299 cells were plated into 6-well plates and a range of *TP53* variants were transfected the following day. Twenty-four hours after transfection, cells were dissociated and plated at a density of 5,$10^3$ cells per well into two 6-well plates in selective media with G418 at a concentration of 1 mg/ml. Cells were then stained after 14 to 16 days with crystal violet. Colony counts of the plates are shown in b and d. The pathogenic variant p.R337H (known as the Brazilian mutation) does not impair cellular growth [5]. p.R175, p.Y234C p.R267Q, p.I254T, and p.R342P: Cancer associated TP53 variants.

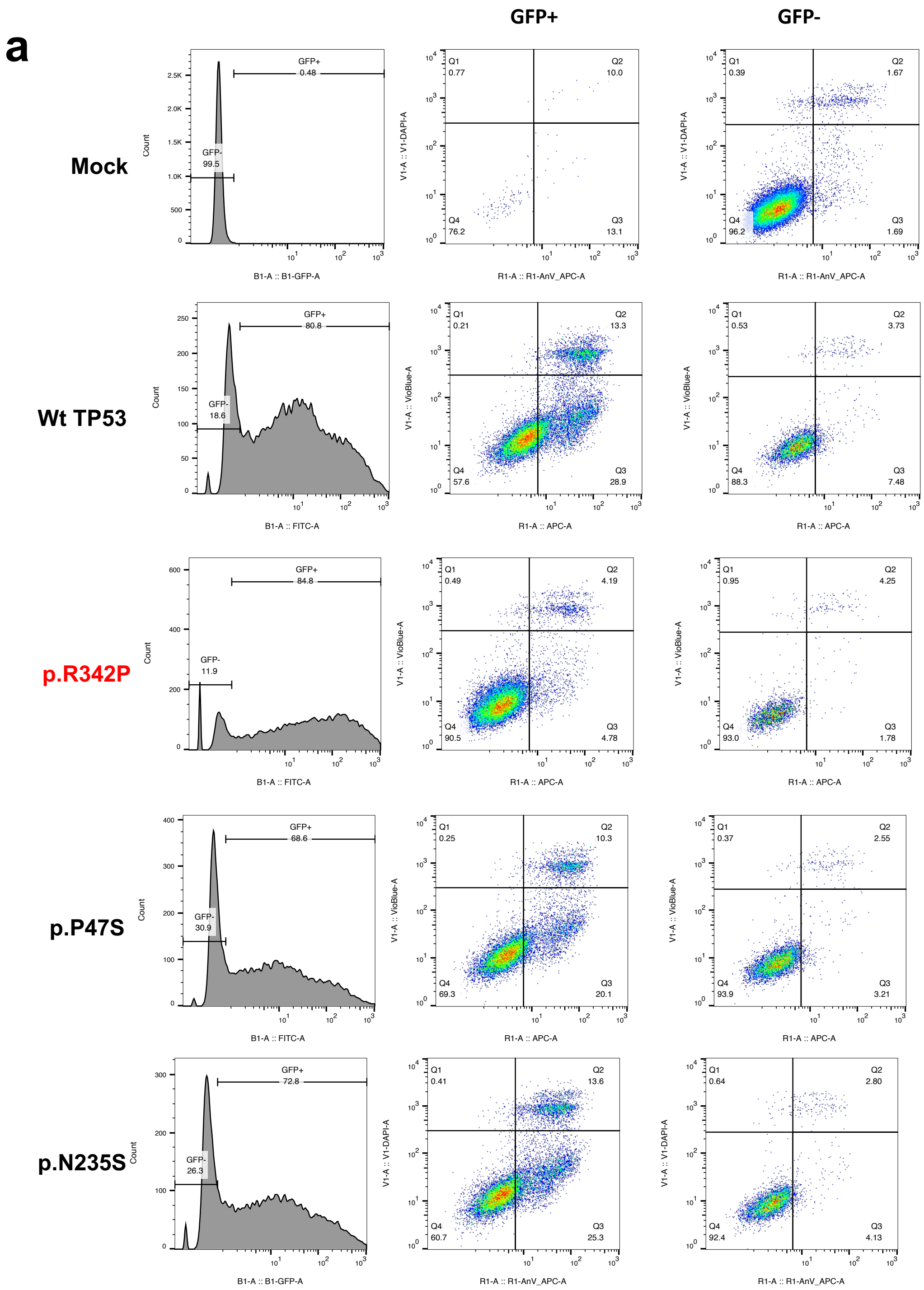


**Fig. S9: Recurrent *TP53* variants are proficient for apoptosis**

(a) H1299 cells were cotransfected with a range of *TP53* variants and a gfp-expressing vector. After 24 hours, the cells were stained with a combination of APC Annexin V and DAPI to assay for viable, early apoptotic, and late apoptotic or necrotic cells. Fluorescence intensities were measured by flow cytometry gating on GFP-negative (non-transfected) versus GFP-positive (transfected) cells. The values shown in the lower left, lower right, and upper right quadrants of each panel represent the percentage of viable, apoptotic, and late apoptotic or necrotic cells, respectively. This figure shows the results for a single experiment. Each *TP53* variant was tested at least three times.

(b) For this experiment, the protocol was identical to S9a except that no GFP was used to select transfected cells.

(c) Bar graph results from another experiment.

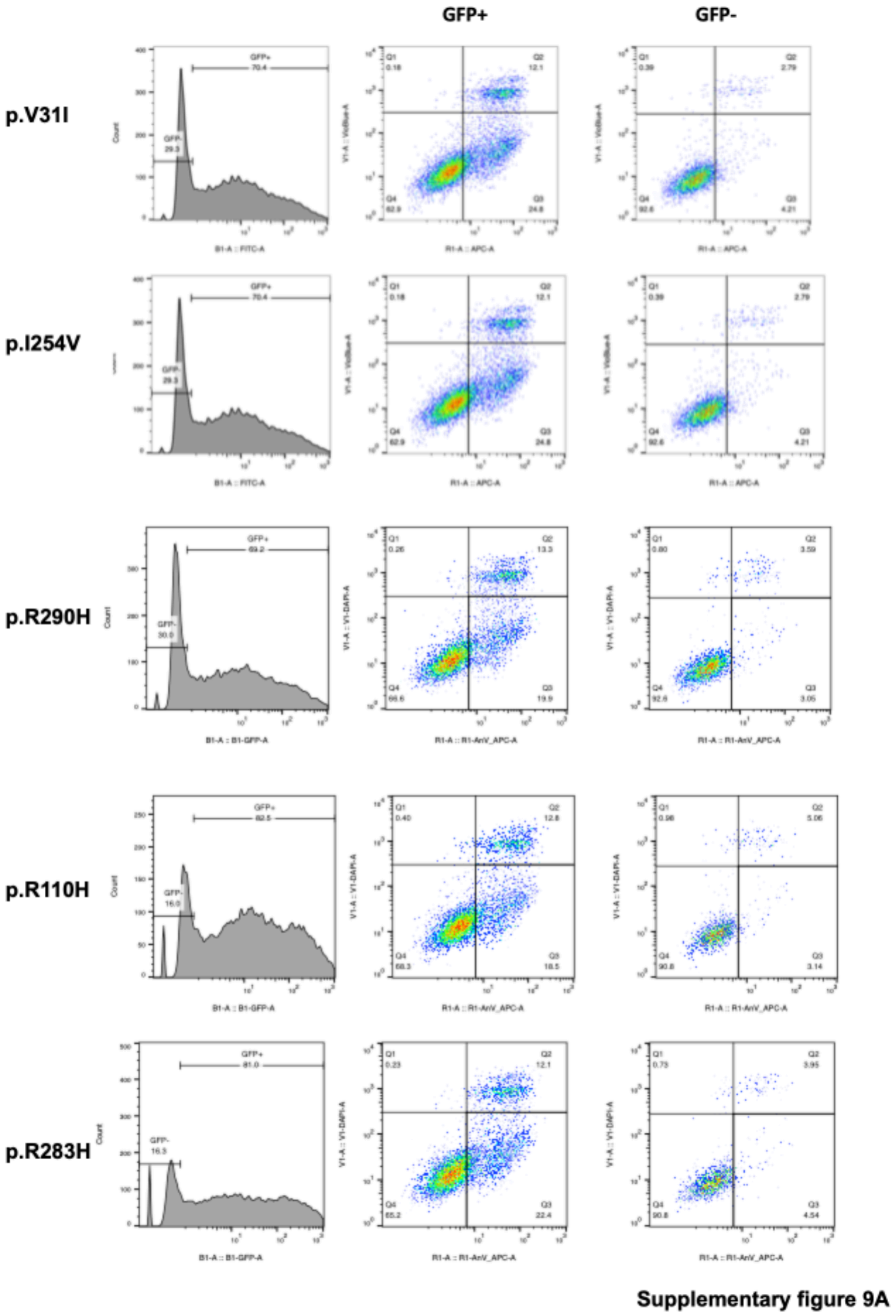


**Fig. S9 (part 2): Recurrent *TP53* variants found in the human population are proficient for apoptosis**
See legend of Fig. S9 (part 1) for further details.

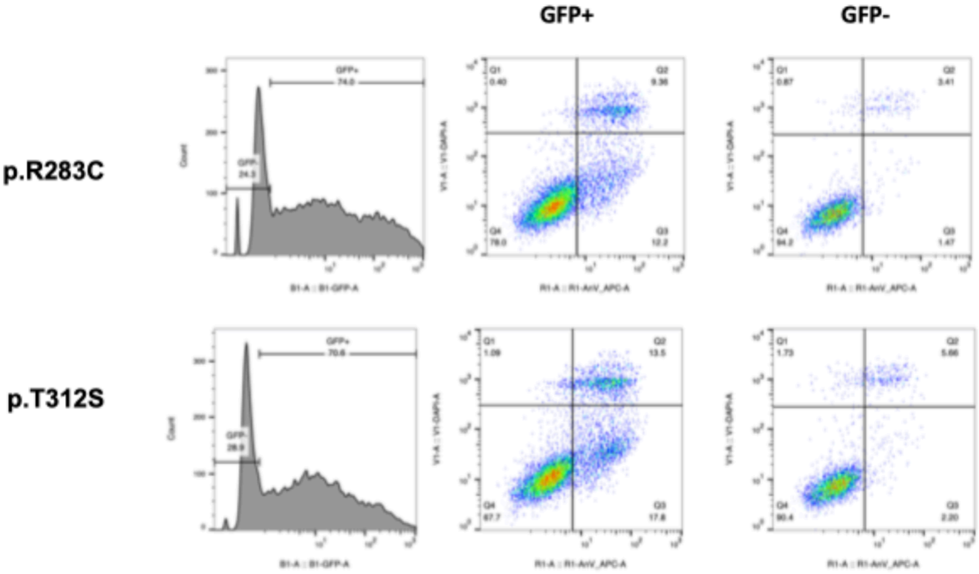


**Fig. S9 (part 3): Recurrent *TP53* variants found in the human population are proficient for apoptosis**
See legend of Fig. S9 (part 1) for further details.

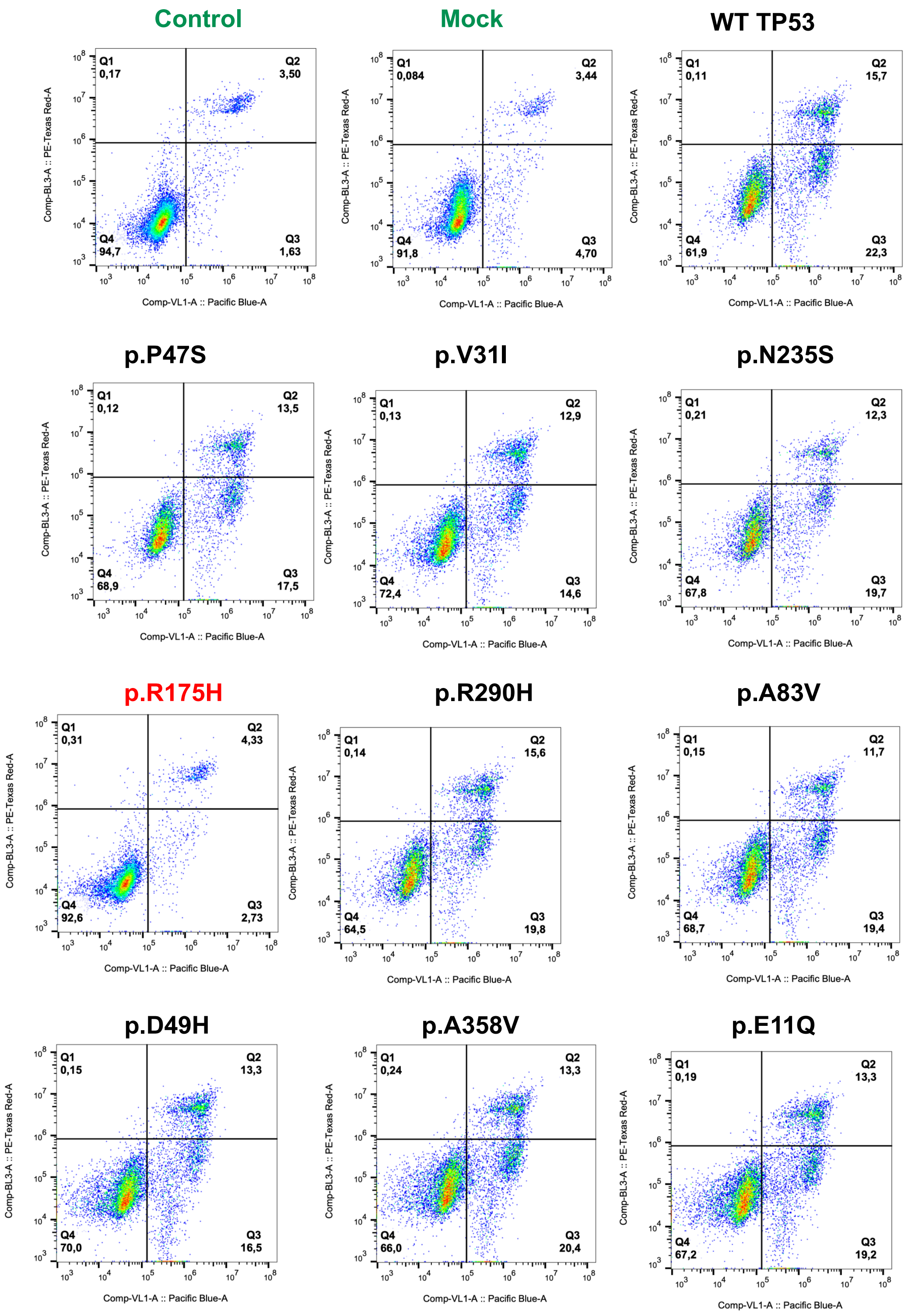


**Fig. S9 (part 4): Recurrent *TP53* variants found in the human population are proficient for apoptosis**

See legend of Fig. S9 (part 1) for further details.

c

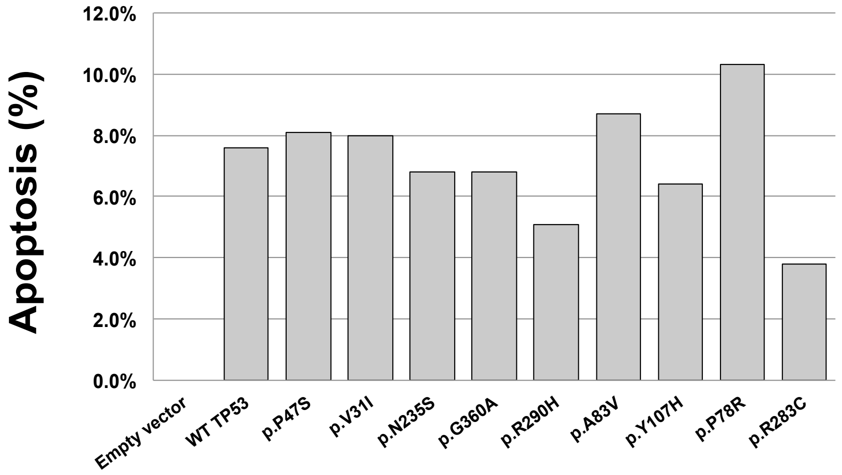


**Fig. S9 (part 5): Recurrent *TP53* variants found in the human population are proficient for apoptosis**
See legend of Fig. S9 (part 1) for further details.

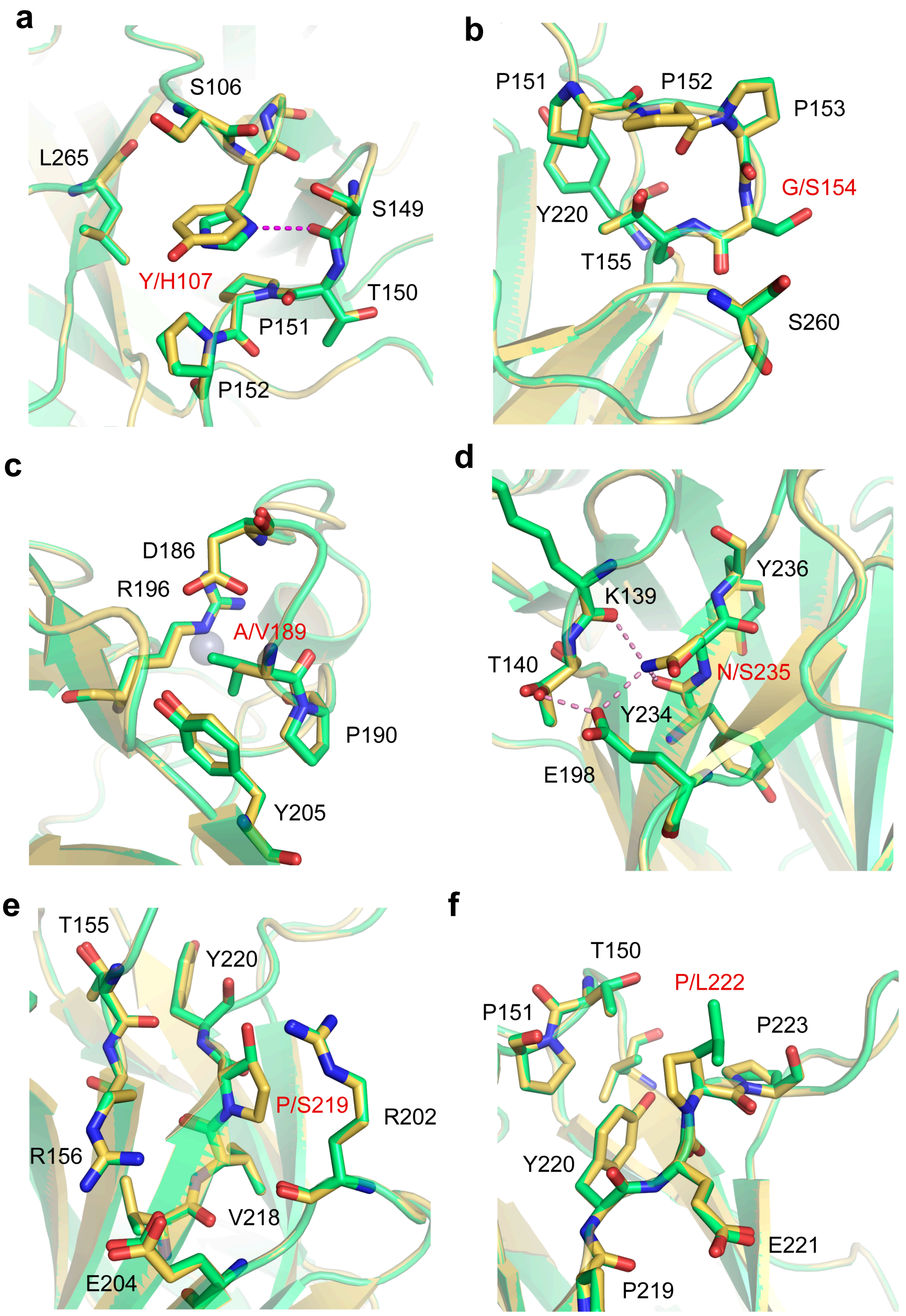


**Fig. S10**: **Structural models of p53 SNP-variant DBDs**

Structures of modelled SNP DBDs (green) are superimposed onto the structure of the wild-type DBD (PDB entry 2XWR, chain A; yellow); (a) Y107H, (b) G154S, (c) A189V, (d) N235S, (e) P219S and (f) P222L. SNP sites are shown as cartoon representations, with key residues highlighted as stick models. Selected side-chain-mediated hydrogen bonds are highlighted with dashed lines.

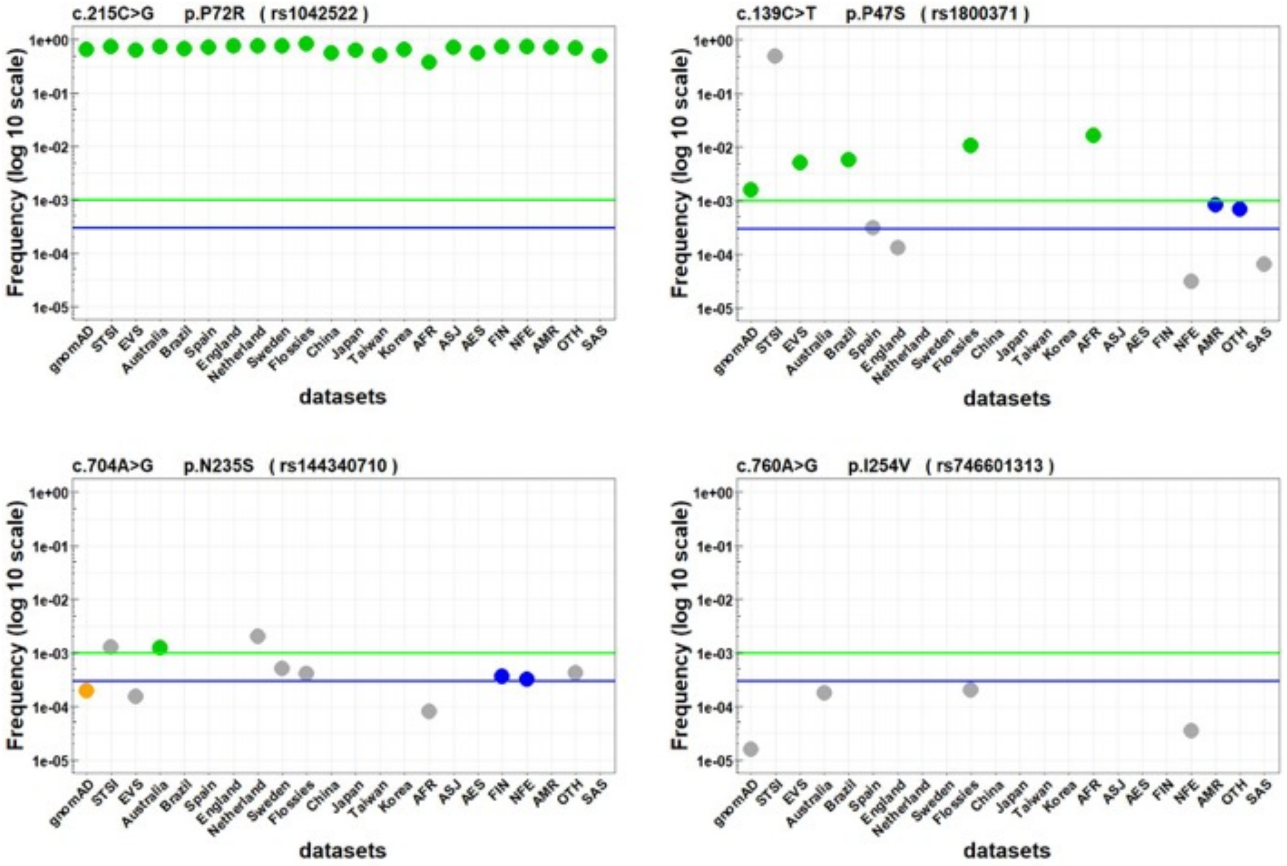


**Fig. S11 (part 1)**: **Allele frequency distribution of *TP53* variants in the included datasets and classification according to ACMG criteria**

For the 14 population datasets used in this study and the eight population-specific subsets of gnomAD, the frequency of each cp*TP53* variant is shown as a colored dot: green: BA1 variants (AF ≥0.001 and AC ≥5); blue: BS1 variants (AF ≥0.0003 and AC ≥5); Orange; variants with an allele count ≥5 but falling short of the BA1 or BS1 allele frequency limits of respectively 0.001 (green line) or 0.0003 (blue line).

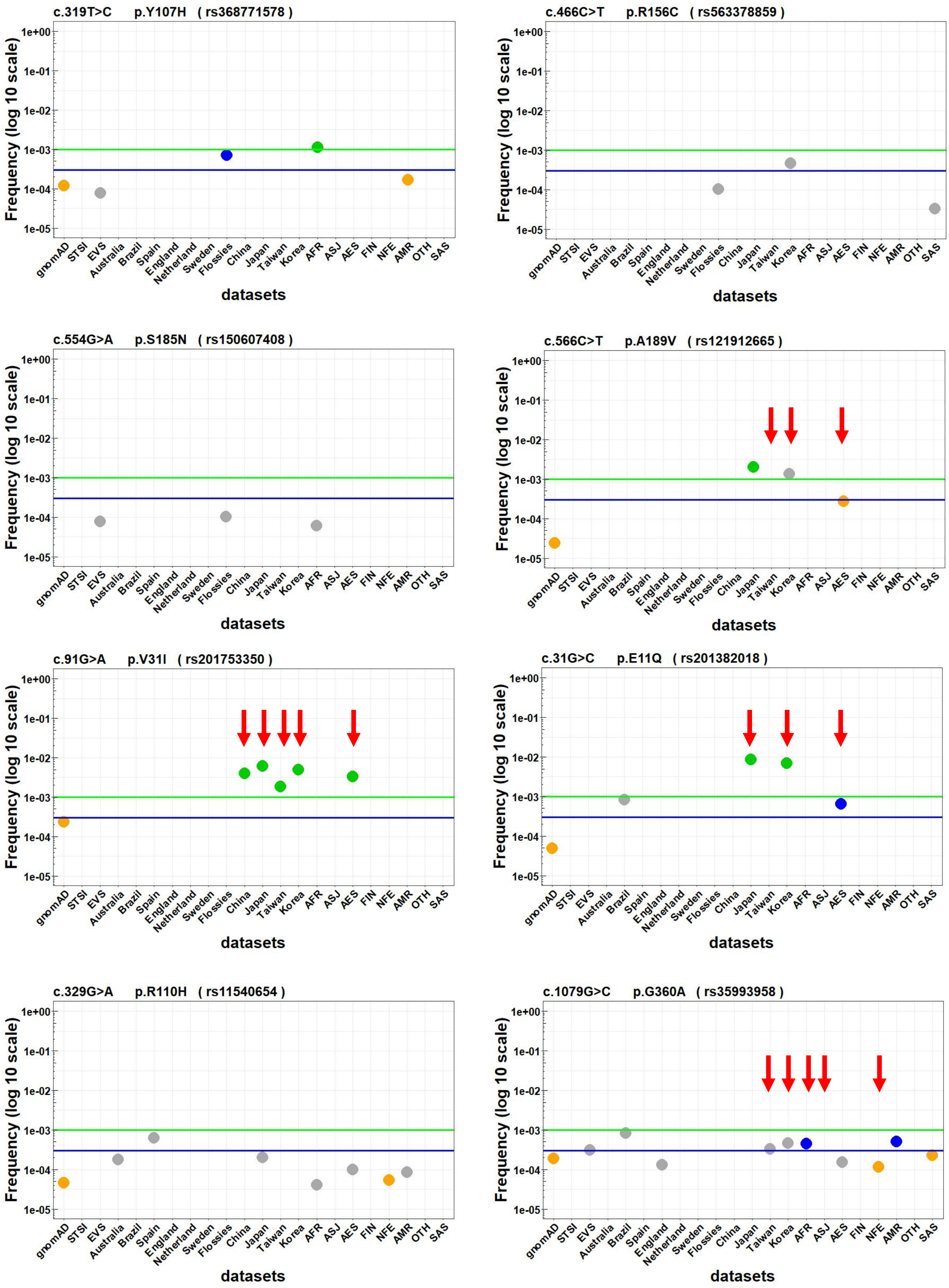


**Fig. S11 part 2**: **Allele frequency distribution of *TP53* variants in the included datasets and classification according to ACMG criteria**

See legend of Fig. S11 (part 1) for further details.

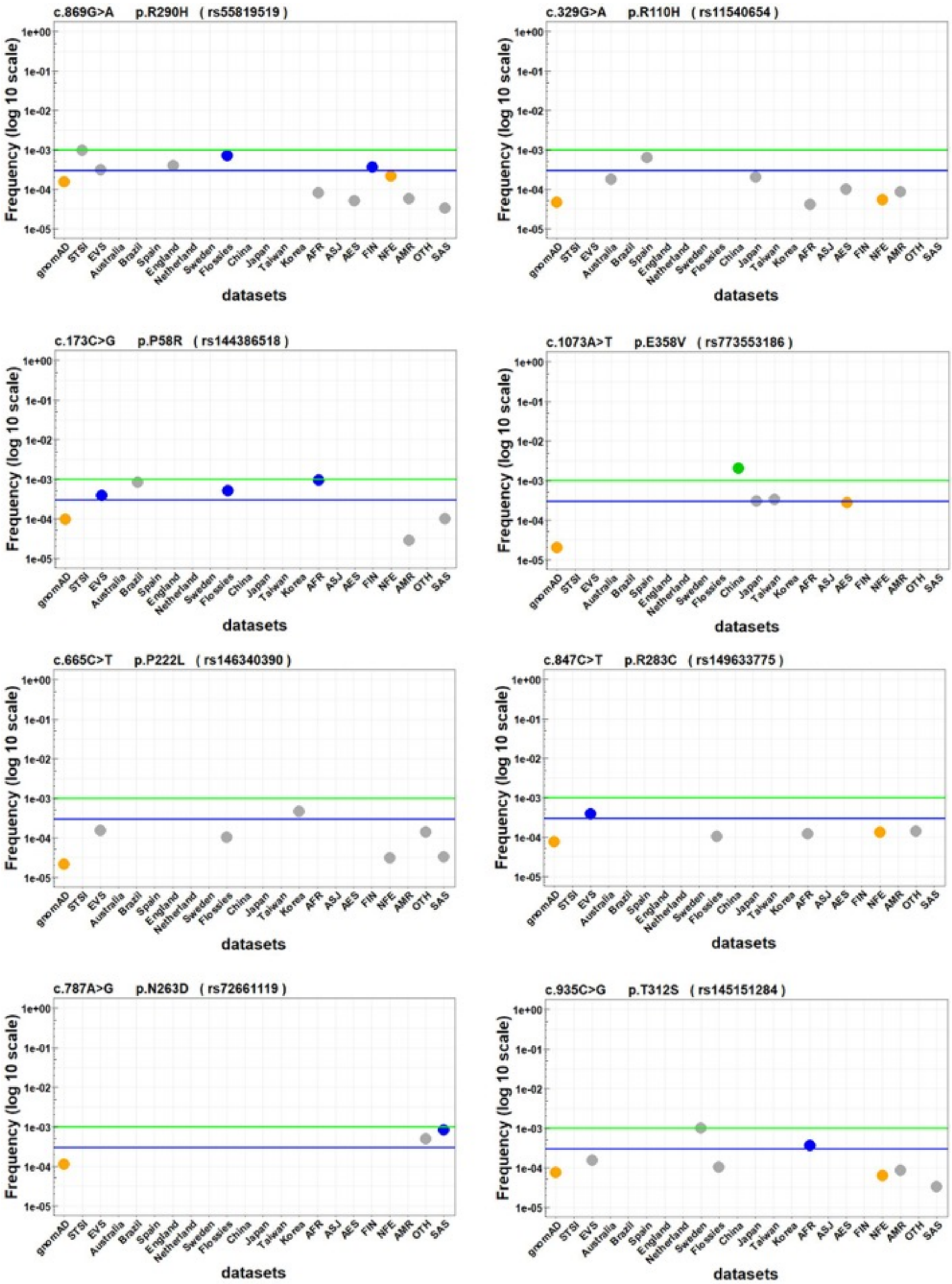


**Fig. S11 part 3**: **Allele frequency distribution of *TP53* variants in the included datasets and classification according to ACMG criteria**

See legend of Fig. S11 (part 1) for further details.

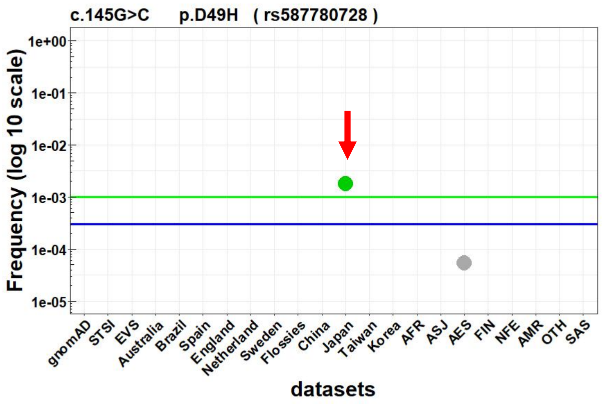


**Fig. S11 part 4**: **Allele frequency distribution of *TP53* variants in the included datasets and classification according to ACMG criteria**

See legend of Fig. S11 (part 1) for further details.

## Table S1: Data sets used for the identification of new constitutional *TP53* variants

| Datasets | URL | version | Number of individuals | reference |
|---|---|---|---|---|
| **Aggregated databases** | | | | |
| GnomAD | http://gnomad.broadinstitute.org/ | gnomAD r2.1.1[1] | 141,456 | [6] |
| STSI | https://genomics.scripps.edu/browser/ | no version last access 12/2017 | 511 | [7] |
| NHLBI GO ESP Exome Variant | https://evs.gs.washington.edu/EVS/ | May 2015 | 6500 | No reference |
| **National databases** | | | | |
| Japan | https://ijgvd.megabank.tohoku.ac.jp/ | 3.5KJPN dataset 28/Oct/2017 | 3,554 | [8] |
| Flossies | https://whi.color.com/ | no version last access 12/2017 | 10,000[2] | No reference |
| Finland | http://www.sisuproject.fi/ | September 16, 2016 [3] | 10,490 | [9] |
| GO_NL (Netherlands) | http://www.nlgenome.nl/ | GoNL SNPs and Indels release 5 | 767 | [10] |
| Spain | http://csvs.babelomics.org/ | no version last access 12/2017 | 1,643[4] | [11] |
| Korea | http://coda.nih.go.kr/coda/KRGDB/index.jsp | 2016 | 1,722 | No info |
| China | https://dx.doi.org/10.6084/m9.figshare.3840339 | no version last access 12/2017 | 11,670[2] | [12] |
| Sweden | https://swefreq.nbis.se/dataset/SweGen | Publication date: 2016-12-23 | 1,000 | [13] |
| UKT (England) | https://www.uk10k.org/data.html | Last edited: 20 Mar 2014 | 10,000 | none |
| Australia | https://sgc.garvan.org.au/initiatives | version last access 10/2019 | 2845 | |
| Taiwan | https://taiwanview.twbiobank.org.tw/ | no version last access 12/2018 | 1517 | |
| Brazil | http://abraom.ib.usp.br/ | 1.02 ; 02/2018 | 609 | [14] |
| **Other databases** | | | | |
| UMD_TP53_database | http://p53.fr/ | October 2017 (2017_R2) | 80,400 tumors, 6,870 different TP53 variants | [15] |
| dbSNSFP | https://sites.google.com/site/jpopgen/dbNSFP | dbNSFP 3.5a (January 2018) | Not relevant | [16] |
| ClinVar | https://www.ncbi.nlm.nih.gov/clinvar/ | no version last access 05/2019 | 1,454 | [17] |
| IARC TP53 database | http://p53.iarc.fr/ | R19, August 2018 | Not relevant | [18] |

[1] Non-cancer version of gnomAD
[2] Females only
[3] Sequencing Initiative Suomi project (SISu), Institute for Molecular Medicine Finland (FIMM), University of Helsinki, Finland (URL: http://sisuproject.fi) [SISu v4.1, date (month, year) accessed].
[4] Neoplasms have been removed

**Table S2: TP53 variants from the 14 datasets used for this study**

**Table S2a: list of the 6001 *TP53* variants identified in the 14 datasets**

**Table S2b: mutational type of *TP53* variants in each dataset**

**Table S2c: List of the 247 missense variants found in the compilation of the 14 population datasets**

<Excel file >

**Table S3: Database entry, publication and ethnicity information for variants found in Asian (5 variants) or Indian (1 variant) populations**

<Excel file >

**Table S4: Data used for the *in silico* predictive and functional analysis of *TP53* variants**

All data were normalized and ranked from 0 to 1 with the lowest score being the most deleterious.

| Predictor | Information | reference |
|---|---|---|
| **Functional data from large-scale analysis of *TP53* variants** | | |
| **WAF1** | Residual transcriptional activity of mutant p53 on the WAF1 promoter | [19] |
| **MDM2** | Residual transcriptional activity of mutant p53 on the MDM2 promoter | |
| **BAX** | Residual transcriptional activity of mutant p53 on the BAX promoter | |
| **14_3_3_σ** | Residual transcriptional activity of mutant p53 on the 14-3-3-σ promoter | |
| **AIP** | Residual transcriptional activity of mutant p53 on the AIP promoter | |
| **GADD45** | Residual transcriptional activity of mutant p53 on the GADD45 promoter | |
| **NOXA** | Residual transcriptional activity of mutant p53 on the NOXA promoter | |
| **p53R2** | Residual transcriptional activity of mutant p53 on the p52R2 promoter | |
| **Kotler et al.** | Functional data from the work of Kotler et al.<br>Only *TP53* variants from codon 100 to 300 were analyzed in this study.<br>Anti-proliferative activity of TP53 variants were tested in mammalian cells (H1299, p53 null). | [3] |
| **Giacomelli et al.** | Functional data from the work of Giacomelli et al.<br>Assay 1: LOF/DNE: Dominant negative activity of TP53 variants toward wild type TP53 in mammalian cells (A549, p53 wt)<br>Assay 2: LOF: Anti-proliferative activity of TP53 variants in mammalian cells (A549, p53 ko)<br>Assay 3: Eto: Cellular response of mammalian cell treated with etoposide (A549, p53 ko) | [20] |

| Predictive rank_score used from dbNSFP | | |
|---|---|---|
| **Sift_rankscore** | Predicted functional effect using SIFT algorithm<br>http://sift.jcvi.org/<br>SIFT (Sorting Intolerant From Tolerant) prediction is based on the degree of conservation of amino acid residues in sequence alignments derived from closely related sequences.<br><br>Ranges from 0 to 1. The amino acid substitution is predicted to be damaging when the score is ≤0.05, and tolerated when the score is >0.05. | [16] |
| **PolyPhen-2 (info)** | **PolyPhen-2** is an automatic tool for prediction of the possible impact of an amino acid substitution on the structure and function of a human protein. This prediction is based on a number of features comprising the sequence, phylogenetic and structural information characterizing the substitution.<br>http://genetics.bwh.harvard.edu/pph2/<br>**Polyphen-2 : prediction_HUMDiv versus prediction_HUMVar**<br>Two pairs of datasets were used to train and test PolyPhen-2 prediction models. The first pair, **HumDiv**, was compiled from all damaging alleles with known effects on the molecular function causing human Mendelian diseases present in the UniProtKB database, together with differences between human proteins and their closely related mammalian homologs, assumed to be non-damaging. The second pair, **HumVar**, consisted of all human disease-causing mutations from UniProtKB, together with common human nsSNPs (MAF>1%) without annotated involvement in disease, which were treated as non-damaging.<br>The user can choose between HumDiv- and HumVar-trained PolyPhen-2 models. More information is available on the PolyPhen web site: http://genetics.bwh.harvard.edu/pph2/dokuwiki/overview | |
| **Polyphen-2_HumVar_ _rankscore** | See above for more info. | |
| **Polyphen-2_HumDiv_ _rankscore** | See above for more info; this prediction is less accurate than HumVar for TP53. | |
| **LRT_converted_rankscore** | LRTori scores were first converted as LRTnew=1-LRTori*0.5 if Omega<1, or LRTnew=LRTori*0.5 if Omega ≥1. Then LRTnew scores were ranked among all LRTnew scores in dbNSFP. The rankscore is the ratio of the rank | |

| | | |
|---|---|---|
| | over the total number of the scores in dbNSFP. The scores range from 0.00166 to 0.85682. | |
| **MutationTaster_converted_rankscore** | The MTori scores were first converted: if the prediction is "A" or "D" MTnew=MTori; if the prediction is "N" or "P", MTnew=1-MTori. Then MTnew scores were ranked among all MTnew scores in dbNSFP. If there are multiple scores of a SNV, only the largest MTnew was used in ranking. The rankscore is the ratio of the rank of the score over the total number of MTnew scores in dbNSFP. The scores range from 0.08979 to 0.81033. | |
| **Mutassessor_rankscore:** | Predicted functional effect using MutAssessor algorithm<br>http://mutationassessor.org/<br>B. Reva, Y. Antipin, C. Sander, *Nucleic Acids Res* **39**, e118 (2011).<br>Functional impact combined score<br>The **default score cutoff** is currently set at -1.938 for classification (i.e. High or medium vs low or neutral). | |
| **VEST3_rankscore** | VEST3_rankscore: VEST3 scores were ranked among all VEST3 scores in dbNSFP. The rankscore is the ratio of the rank of the score over the total number of VEST3 scores in dbNSFP. When there are multiple scores for the same variant, the largest score (most damaging) is presented. The scores range from 0 to 1. VEST score is free for non-commercial use. For more details see http://wiki.chasmsoftware.org/index.php/SoftwareLicense. Commercial users should contact the Johns Hopkins Technology Transfer office. | |
| **Provean_rankscore** | Predicted functional effect using PROVEAN algorithm<br>http://provean.jcvi.org/index.php<br>**PROVEAN** (**Pro**tein **V**ariation **E**ffect **An**alyzer) is a software tool that predicts whether an amino acid substitution or indel has an impact on the biological function of a protein.<br><br>PROVEAN introduces a **delta alignment score** based on the reference and variant versions of a protein query sequence with respect to sequence homologues collected from the NCBI NR protein database through BLAST. | |

| | | |
|---|---|---|
| | For maximum separation of deleterious and neutral variants for all four classes of human protein variants, the **default score cutoff** is currently set at -2.5 for binary classification (i.e. deleterious vs neutral). | |
| **GERP_RS_rankscore** | GERP++ RS scores were ranked among all GERP++ RS scores in dbNSFP. The rankscore is the ratio of the rank of the score over the total number of GERP++ RS scores in dbNSFP. | |
| **SiPhy_29way_logOdds_rankscore** | SiPhy_29way_logOdds_rankscore: SiPhy_29way_logOdds scores were ranked among all SiPhy_29way_logOdds scores in dbNSFP. The rankscore is the ratio of the rank of the score over the total number of SiPhy_29way_logOdds scores in dbNSFP. | |
| **phyloP100way_vertebrate_rankscore** | phyloP100way_vertebrate scores were ranked among allphyloP100way_vertebrate scores in dbNSFP. The rankscore is the ratio of the rank of the score over the total number of phyloP100way_vertebrate scores in dbNSFP. | |
| **phastCons100way_vertebrate_rankscore** | phastCons100way_vertebrate_rankscore: phastCons100way_vertebrate scores were ranked among all phastCons100way_vertebrate scores in dbNSFP. The rankscore is the ratio of the rank of the score over the total number of phastCons100way_vertebrate scores in dbNSFP. | |
| **CADD_raw_rankscore** | CADD raw scores were ranked among all CADD raw scores in dbNSFP. The rankscore is the ratio of the rank of the score over the total number of CADD raw scores in dbNSFP. Please note the following copyright statement for CADD: "CADD scores (http://cadd.gs.washington.edu/) are Copyright 2013 University of Washington and Hudson-Alpha Institute for Biotechnology (all rights reserved) but are freely available for all academic, non-commercial applications. For commercial licensing information contact Jennifer McCullar (mccullaj@uw.edu). | |
| **DANN_rank_score** | DANN scores were ranked among all DANN scores in dbNSFP. The rankscore is the ratio of the rank of the score over the total number of DANN scores in dbNSFP. | |
| **Predictive data from various software** | | |
| **REVEL** | The Rare Exome Variant Ensemble Learner REVEL is an ensemble method for predicting the pathogenicity of missense variants. It integrates scores | [21] |

| | | |
|---|---|---|
| | from MutPred, FATHMM v2.3, VEST 3.0, PolyPhen-2, SIFT, PROVEAN, MutationAssessor, MutationTaster, LRT, GERP++, SiPhy, phyloP, and phastCons. | |
| **PON-P2** | Sorts amino acid substitutions into three categories: pathogenic, neutral or unknown tolerance. PON-P2 employs a machine learning method, grouping the variants into pathogenic, neutral and unknown classes, on the basis of a random forest probability score. It uses data on evolutionary conservation of sequences, physical and biochemical properties of amino acids, gene ontology (GO) annotations and, if available, functional annotations of variation sites. | [22] |
| **Envision** | Envision combines 21,026 variant effect measurements from nine large-scale experimental mutagenesis datasets, a hitherto untapped training resource, with a supervised, stochastic gradient boosting learning algorithm. | [23] |
| **MutPred2** | MutPred2 is a standalone and web application developed to classify amino acid substitutions as pathogenic or benign in humans. In addition, it predicts their impact on over 50 different protein properties and, thus, enables the inference of molecular mechanisms of pathogenicity. | [24] |
| **RF_Score** | MutPred is based upon SIFT and a gain/loss of 14 different structural and functional properties. For instance, gain of helical propensity or loss of a phosphorylation site. It was trained using the deleterious mutations from the Human Gene Mutation Database and neutral polymorphisms from Swiss-Prot. The current version of MutPred is 1.2. The update consists of replacing the SIFT score by a more stable version of code that calculates evolutionary conservation. | [25] |

**Table S5: Functional analysis and classification of *TP53* variant from set 21**

<Excel file >

**Table S6: Predicted stability of p53 DBD SNPs and oncogenic variants**

| DBD variant | $\Delta T_m$ (calc.)[1] (°C) | $\Delta T_m$ (exp.)[1] (°C) |
|---|---|---|
| p.Y107H | -1.7 | - |
| p.G154S | -3.8 | - |
| p.A189V | -0.9 | - |
| p.P219S | -3.8 | - |
| p.P222L | -1.6 | - |
| p.N235S | -1.8 | - |
| p.R283C | -1.6 | - |
| p.R290H | -1.1 | - |
| p.V143A | -7.5 | -6.9[2] |
| p.Y220H | -6.1 | -6.2[3] |
| p.Y220C | -5.6 | -7.8[3] |
| p.R249S | -5.4 | -4.9[2] |
| p.R273H | -0.9 | -0.1[2] |
| p.R282W | -0.6 | ~ -7[4] |

[1] $\Delta T_m = T_m$ (wild-type) - $T_m$ (mutant); either calculated with HoTMuSiC [26] (calc.) or determined experimentally (exp.).

[2] Based on the $T_m$ values in [27]

[3] Joerger AC, unpublished data.

[4] Estimated $T_m$ shift based on the fact that the mutant has the same thermodynamic stability as the V143A mutant upon urea denaturation [27].

## References


1. Edlund K, Larsson O, Ameur A, Bunikis I, Gyllensten U, Leroy B *et al.* Data-driven unbiased curation of the TP53 tumor suppressor gene mutation database and validation by ultradeep sequencing of human tumors. Proc Natl Acad Sci U S A.2012; 109:9551-9556
2. Pal K, Bystry V, Reigl T, Demko M, Krejci A, Touloumenidou T *et al.* GLASS: assisted and standardized assessment of gene variations from Sanger sequence trace data. Bioinformatics.2017; 33:3802-3804
3. Kotler E, Shani O, Goldfeld G, Lotan-Pompan M, Tarcic O, Gershoni A *et al.* A Systematic p53 Mutation Library Links Differential Functional Impact to Cancer Mutation Pattern and Evolutionary Conservation. Mol Cell.2018; 71:178-190.e8
4. Grochova D, Vankova J, Damborsky J, Ravcukova B, Smarda J, Vojtesek B *et al.* Analysis of transactivation capability and conformation of p53 temperature-dependent mutants and their reactivation by amifostine in yeast. Oncogene.2008; 27:1243-1252
5. Ribeiro RC, Sandrini F, Figueiredo B, Zambetti GP, Michalkiewicz E, Lafferty AR *et al.* An inherited p53 mutation that contributes in a tissue-specific manner to pediatric adrenal cortical carcinoma. Proc Natl Acad Sci U S A.2001; 98:9330-9335
6. Lek M, Karczewski KJ, Minikel EV, Samocha KE, Banks E, Fennell T *et al.* Analysis of protein-coding genetic variation in 60,706 humans. Nature.2016; 536:285-291
7. Erikson G, Bodian D, Rueda M, Molparia B, Scott E, Scott-Van Zeeland A *et al.* Whole-Genome Sequencing of a Healthy Aging Cohort. Cell.2016; 165:1002-1011
8. Nagasaki M, Yasuda J, Katsuoka F, Nariai N, Kojima K, Kawai Y *et al.* Rare variant discovery by deep whole-genome sequencing of 1,070 Japanese individuals. Nat Commun.2015; 6:8018
9. Lim ET, Würtz P, Havulinna AS, Palta P, Tukiainen T, Rehnström K *et al.* Distribution and medical impact of loss-of-function variants in the Finnish founder population. PLoS Genet.2014; 10:e1004494
10. Genome OTNC Whole-genome sequence variation, population structure and demographic history of the Dutch population. Nat Genet.2014; 46:818-825
11. Dopazo J, Amadoz A, Bleda M, Garcia-Alonso L, Alemán A, García-García F *et al.* 267 Spanish Exomes Reveal Population-Specific Differences in Disease-Related Genetic Variation. Mol Biol Evol.2016; 33:1205-1218
12. Cai N, Bigdeli TB, Kretzschmar WW, Li Y, Liang J, Hu J *et al.* 11,670 whole-genome sequences representative of the Han Chinese population from the CONVERGE project. Sci Data.2017; 4:170011
13. Ameur A, Dahlberg J, Olason P, Vezzi F, Karlsson R, Martin M *et al.* SweGen: a whole-genome data resource of genetic variability in a cross-section of the Swedish population. Eur J Hum Genet.2017; 25:1253-1260
14. Naslavsky MS, Yamamoto GL, de Almeida TF, Ezquina SAM, Sunaga DY, Pho N *et al.* Exomic variants of an elderly cohort of Brazilians in the ABraOM database. Hum Mutat.2017; 38:751-763
15. Leroy B, Ballinger ML, Baran-Marszak F, Bond GL, Braithwaite A, Concin N *et al.* Recommended Guidelines for Validation, Quality Control, and Reporting of TP53 Variants in Clinical Practice. Cancer Res.2017; 77:1250-1260

16. Liu X, Wu C, Li C, Boerwinkle E dbNSFP v3.0: A One-Stop Database of Functional Predictions and Annotations for Human Nonsynonymous and Splice-Site SNVs. Hum Mutat.2016; 37:235-241
17. Landrum MJ, Lee JM, Benson M, Brown GR, Chao C, Chitipiralla S *et al.* ClinVar: improving access to variant interpretations and supporting evidence. Nucleic Acids Res.2018; 46:D1062-D1067
18. Hainaut P, Pfeifer GP Somatic TP53 Mutations in the Era of Genome Sequencing. Cold Spring Harb Perspect Med.2016; 6
19. Kato S, Han SY, Liu W, Otsuka K, Shibata H, Kanamaru R *et al.* Understanding the function-structure and function-mutation relationships of p53 tumor suppressor protein by high-resolution missense mutation analysis. Proc Natl Acad Sci U S A.2003; 100:8424-8429
20. Giacomelli AO, Yang X, Lintner RE, McFarland JM, Duby M, Kim J *et al.* Mutational processes shape the landscape of TP53 mutations in human cancer. Nat Genet.2018; 50:1381-1387
21. Ioannidis NM, Rothstein JH, Pejaver V, Middha S, McDonnell SK, Baheti S *et al.* REVEL: An Ensemble Method for Predicting the Pathogenicity of Rare Missense Variants. Am J Hum Genet.2016; 99:877-885
22. Niroula A, Urolagin S, Vihinen M PON-P2: prediction method for fast and reliable identification of harmful variants. PLoS One.2015; 10:e0117380
23. Gray VE, Hause RJ, Luebeck J, Shendure J, Fowler DM Quantitative Missense Variant Effect Prediction Using Large-Scale Mutagenesis Data. Cell Syst.2018; 6:116-124.e3
24. Pejaver V, Urresti J, Lugo-Martinez J, Pagel KA, Lin GN, Nam H *et al.* MutPred2: inferring the molecular and phenotypic impact of amino acid variants. bioRxiv134981
25. Li B, Krishnan VG, Mort ME, Xin F, Kamati KK, Cooper DN *et al.* Automated inference of molecular mechanisms of disease from amino acid substitutions. Bioinformatics.2009; 25:2744-2750
26. Harris CC The 1995 Walter Hubert Lecture--molecular epidemiology of human cancer: insights from the mutational analysis of the p53 tumour-suppressor gene. Br J Cancer.1996; 73:261-269
27. Jeffrey PD, Gorina S, Pavletich NP Crystal structure of the tetramerization domain of the p53 tumor suppressor at 1.7 angstroms. Science.1995; 267:1498-1502
